# Comprehensive Review of Fungi on Coffee

**DOI:** 10.3390/pathogens11040411

**Published:** 2022-03-28

**Authors:** Li Lu, Saowaluck Tibpromma, Samantha C. Karunarathna, Ruvishika S. Jayawardena, Saisamorn Lumyong, Jianchu Xu, Kevin D. Hyde

**Affiliations:** 1 Innovative Institute of Plant Health, Zhong Kai University, Guangzhou 510550, China; 6371105004@lamduan.mfu.ac.th; 2 Center of Excellence in Fungal Research, Mae Fah Luang University, Chiang Rai 57100, Thailand; ruvishika.jay@mfu.ac.th; 3 School of Science, Mae Fah Luang University, Chiang Rai 57100, Thailand; 4 Center for Yunnan Plateau Biological Resources Protection and Utilization, College of Biological Resource and Food Engineering, Qujing Normal University, Qujing 655011, China; saowaluckfai@gmail.com (S.T.); samanthakarunarathna@gmail.com (S.C.K.); 5 Research Center of Microbial Diversity and Sustainable Utilization, Chiang Mai University, Chiang Mai 50200, Thailand; scboi009@gmail.com; 6 Department of Biology, Faculty of Science, Chiang Mai University, Chiang Mai 50200, Thailand; 7 Academy of Science, The Royal Society of Thailand, Bangkok 10300, Thailand; 8 Center for Mountain Futures, Kunming Institute of Botany, Chinese Academy of Sciences, Kunming 650201, China; jxu@mail.kib.ac.cn; 9 CIFOR-ICRAF China Country Program, World Agroforestry Centre, Kunming 650201, China

**Keywords:** endophytes, fungal diseases, fungal toxins, pathogens, postharvest diseases

## Abstract

Coffee is grown in more than 80 countries as a cash crop and consumed worldwide as a beverage and food additive. It is susceptible to fungal infection during growth, processing and storage. Fungal infections, in particular, can seriously affect the quality of coffee and threaten human health. The data for this comprehensive review were collected from the United States Department of Agriculture, Agricultural Research Service (USDA ARS) website and published papers. This review lists the fungal species reported on coffee based on taxonomy, life mode, host, affected plant part and region. Five major fungal diseases and mycotoxin-producing species (post-harvest diseases of coffee) are also discussed. Furthermore, we address why coffee yield and quality are affected by fungi and propose methods to control fungal infections to increase coffee yield and improve quality. Endophytic fungi and their potential as biological control agents of coffee disease are also discussed.

## 1. Introduction

Coffee has gained in popularity in modern times and is the second-best-selling beverage in the world [1]. As an important economic crop, it is central to the livelihoods of millions of people worldwide [2]; accordingly, more than 80 countries grow coffee and some countries use coffee as a major cash crop [3]. World coffee production for 2020/2021 is forecast to be 5.5 million kg higher than the previous year, reaching a record 176.1 million kg [4]. Brazil is the largest exporter of coffee, and its exports account for one-third of the global total [5]. The Agricultural Trade Office in Sao Paulo (ATO) forecasts the Brazilian coffee production for 2020/2021 at a record of 67.9 million kg, an increase of 15% over 2019 output. Finland is the largest per capita consumer of coffee, while China consumes the most coffee by volume [6]. *Coffea arabica* and *C. canephora* (robusta) are the two most-grown coffee species in the world [7], accounting for 60% and 40% of global production, respectively [8].

Throughout the tropics, coffee growers face many problems in agricultural production [9]. As a climate-sensitive plant, implications of climate change have altered coffee production, from decreasing crop yield and quality to increasing fungal diseases and invasive pests [10]. Coffee worldwide suffers from a range of pests and diseases, and fungal infections are also a major problem [11]. Coffee roots, stems, leaves and beans are often damaged by pests and pathogens [12].

Fungi on coffee occur in different life modes: endophytes, pathogens and saprobes [13,14,15]. The largest number of fungi have been recorded from *C. arabica* and *C. canephora* (Table S1). Endophytes usually live inside the host without causing injury or obvious symptoms, and this association can provide a better living environment for both the host and fungus [16]. There are also reports on their ability to aid in the defense of host plants [17,18]. Huang et al. [19] screened potential antagonistic endophytes that prevent and control post-harvest diseases. Coffee easily can be infected by pathogenic fungi when growing, during post-harvest handling and storage, and during processing [20]. One of the most virulent diseases is ‘coffee rust’ caused by *Hemileia vastatrix*, which wiped out coffee 150 years ago and continues to cause problems in coffee plantations worldwide [21,22]. Fungal diseases in coffee can be divided into two types: diseases in pre-harvest and diseases in post-harvest [23,24]. Many post-harvest coffee pathogens are infected shortly before harvest, are generally not found at harvest, and feature low activity; moreover, poor storage conditions during post-harvest favour their development [23]. Fungal invasions before harvest are mainly induced by the interaction between the plant host and other organisms (such as insects), while fungal infections after harvest are controlled by nutrient availability, temperature, humidity and biological factors (insects) [25]. Another pathway is that endophytic fungi in coffee beans change their life modes to saprobic/pathogenic after the beans are harvested, becoming postharvest pathogens [19,26]. Most postharvest fungi produce toxins as secondary metabolites viz ochratoxin-A, which is a mycotoxin mainly produced as a result of secondary metabolism of many species of *Aspergillus* and *Penicillium* and is the most common mycotoxin present in agricultural commodities [27]. Toxin-producing fungi can be isolated from coffee beans both pre-harvest and post-harvest, while the risk of fungal growth and mycotoxin production after harvest is higher in high temperature areas [28,29]. These toxins can cause host infections and reduce coffee bean quality [30] and can be carcinogenic, hepatotoxic, hematotoxic, nephrotoxic and neurotoxic for humans [31,32]. Silva et al. [33] isolated ochratoxin-A from damaged coffee beans, and ochratoxin-A was shown to cause coffee quality and yield losses. Studies have shown that the main toxigenic fungal genera comprise *Aspergillus*, *Penicillium* and *Fusarium*, which are natural coffee contaminants [34], and they can infect hosts in both farms and warehouses [35]. 

In our review, 966 fungal records belonging to 113 genera and 648 species found on coffee are reported (Table S1). The purpose of this review is to discuss the fungal taxa reported on coffee based on taxonomy, life mode, host, affected plant part and region, and also discuss the roles of endophytes. In addition, this review provides a comprehensive up-to-date list of coffee fungi found worldwide and proposes recommendations for preventing fungal pathogen infections. This review provides (1) preliminary information on coffee fungi, (2) summarizes the main factors of coffee loss, and (3) suggestions for improving coffee yield and quality.

## 2. Results

### 2.1. Records of Coffee Fungi

**Figure 1:**
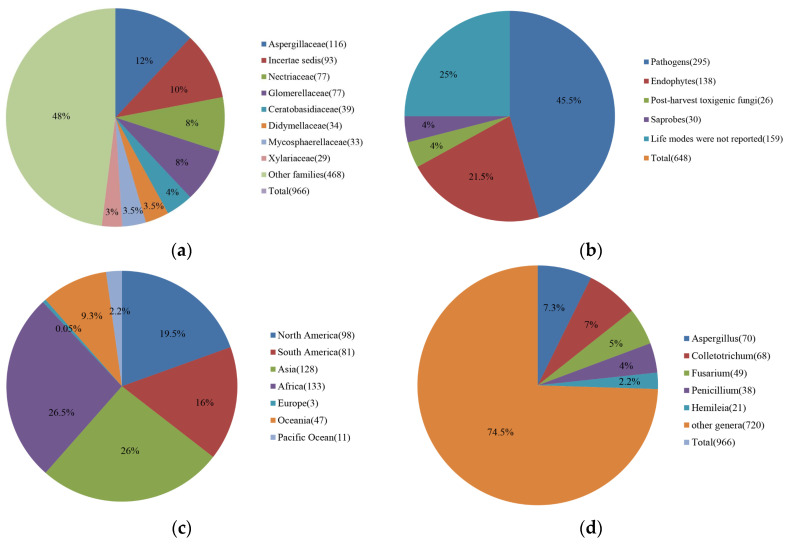
(**a**) Percentages of different fungal families reported on coffee; (**b**) Percentages of different life modes of fungi reported on coffee; (**c**) Percentages of coffee pathogens reported in different countries; (**d**) Percentages of most frequently reported fungal pathogenic and toxigenic fungal genera on coffee.

A total of 966 records of coffee fungi (Supplementary Table S1) were found in the literatures, belonging to 113 genera of which frequently found families and genera are shown in Figure 1a and Supplementary Table S2.

Out of 648 fungal species, 295 are pathogenic, 138 are endophytes, and 30 are saprobes, while the life modes of 159 species have not been confirmed and another 26 species are post-harvest disease causing agents that can produce mycotoxins in dried and green coffee beans (Figure 1a,b). It was not confirmed in reported publications whether most reported pathogens caused pre-harvest or post-harvest infections. Of the 295 species of pathogens, 212 species are true pathogens (TP), three species are post-harvest pathogens (PP) and four species are both true pathogens (TP) and post-harvest pathogens (PP), while the other 76 species are listed as unknown (UNK) as their disease symptoms have not been confirmed on coffee (Supplementary Table S4).

### 2.2. Endophyte Role in Coffee Plants

Endophytic fungi that can inhibit nematodes, coffee berry borers and pathogenic fungi have the potential to be used as biocontrol agents to control pest and pathogen infections of coffee [36,37,38]. Goates et al. [39] showed that some endophytic fungi reduce fungal diseases by producing volatile organic compounds that can kill or inhibit phytopathogens. Monteiro et al. [40] demonstrated that volatile organic compounds produced by the endophyte *Muscodor coffeanum* isolated from *C. arabica* produce fungicidal activity against *Aspergillus ochraceus*. Three records of *Muscodor* species belonging to induratiaceae [41] were found in coffee according to our data. De Almeida et al. [42] isolated *Aspergillus* sp., *A. westerdijkiae*, *A. niger*, *A. tamari,* and *A. fumigatus*, *Lichtheimia ramosa* and *Rhizopus oryzae* from coffee beans to test their abilities to inhibit the growth of *Aspergillus* species and ochratoxin-A production, and *A. niger* showed the best inhibitory ability of both growth and ochratoxin-A production. According to our results, Aspergillaceae is the most frequently found family in coffee. Furthermore, Eida et al. [43] isolated *Penicillium crustosum*, *Penicillium verruculosum*, *Trichoderma harzianum,* and *Hypocrea lixii* from coffee residue compost that can aid in the degradation of lignocellulose waste.

### 2.3. Pathogen Effect on Coffee and Coffee Disease

Among 648 species, fungal pathogens are the most common (295 species) as shown in Figure 1b. According to Supplementary Tables S1 and S3: Sixty-eight of *Colletotrichum* belonging to 35 species have been reported. Coffee production is often affected by Coffee Berry Disease, which is the main factor limiting the production of *C. arabica* in Kenya and other countries in East Africa, especially in high-altitude areas. It is potentially responsible for 50 to 80% of total crop losses [44]. Forty-nine *Fusarium* belonging to 23 species have been reported (Supplementary Tables S1 and S3). *Fusarium* species are one of the most important phytopathogenic and toxin-producing fungi [45]. Coffee Wilt Disease is a devastating disease in East and Central Africa [46]. Coffee Wilt Disease is a vascular disease, and due to its high transmission potential, Coffee Wilt Disease poses a threat to all coffee-producing regions. This disease can kill its host at all ages in a short time [47]. Moreover, after the infected trees and their roots are removed, the infested soil may remain infectious for several years [48]. Twenty-one *Hemileia* have been reported belonging to two species, and *Hemileia vastatrix* is an important phytopathogenic fungus that causes Coffee Leaf Rust. Coffee Leaf Rust, one of the major diseases of Arabica coffee is a major threat to coffee production worldwide and it has been reported to cause serious economic losses in more than 50 coffee-growing countries [49]. Brown Eye Spot or *Cercospora* Blotch caused by *Cercospora coffeicola*, has been reported on coffee. Besides, Andrade et al. [50] showed that isolates producing brown eye spot and black spot can also cause prompt alterations in the antioxidant metabolism of coffee leaves. *Armillaria* root rot caused by *Armillaria* sp., has been reported on coffee, and this disease leads to coffee plant rot and the eventual death of the plant. Since this fungus invades deeper into roots, symptoms are difficult to detect, thus it can last for several years before symptoms appear on the surface. This disease spreads to other plants with the transfer of soil [51]. Major coffee diseases that have a huge impact on coffee plantations worldwide are discussed in detail below [14].

#### 2.3.1. Detection and Identification of Diseases

Detection and identification of fungal diseases in crops can be done through direct and indirect methods. Direct detection of fungal diseases includes molecular and serological methods, while indirect methods identify the plant diseases through various parameters such as morphological change, temperature change, transpiration rate change, and volatile organic compounds released by infected plants [52]. Among different methods, fungal morphology is a commonly used method to identify coffee pathogens [11], while pathogenicity tests [53] and Polymerase Chain Reaction (PCR) [54] are also used. Generally, three main methods are used for the identification of coffee fungal pathogens.

Fungal morphological characteristics—Different fungal pathogens cause different symptoms on the host surface [14]. Firstly, the disease symptoms on the host are observed and recorded, and then the pure cultures grown on potato dextrose agar (PDA) are obtained according to the isolation method of Senanayake et al. [55]. Finally, colony size, colour of the conidial masses and zonation, size, and shape of conidia harvested from the cultures are recorded under the microscope [44]. 

Pathogenicity test (Koch’s postulates)—Spore suspensions of pathogenic fungi are obtained by pure cultures grown on PDA for 7 to 10 days, prepare healthy/disease-free hosts, then carry out the pathogenicity test [44]. After inoculation, the changes are recorded from 1 to 15 days of growth, compare the morphological characteristics of the lesions in the host with original disease lesions.

Polymerase Chain Reaction—Pure cultures of pathogenic fungi grown on PDA for 7 to 10 days are used to scrape mycelium from the culture surface. Then, the genomic DNA is extracted using a Genomic DNA Extraction Kit or CTAB. Finally, PCR amplification is done for the specific genes of interest [44].

#### 2.3.2. Coffee Leaf Rust

- Pathogen: *Hemileia vastatrix* has spread to all coffee cultivation areas worldwide. *Hemileia coffeicola* is restricted to central and western Africa, especially in higher and cooler regions [22,56].
- Hosts: *C. arabica* (arabica coffee) and *C. canephora* (robusta coffee), the two most important commercial coffee species [56].
- Symptoms and signs: Infection occurs on the leaves of coffee. The first observable symptoms are small, and light-yellow spots on the upper surface of leaves. As the diameter of these points gradually increases, a large number of orange urediniospores (=uredospores) appear under the leaf surface. The fungus forms spores through stomata instead of penetrating the epidermis like most rust-causing species, so it does not form many typical rust pustules. Powdery lesions under leaves appear orange-yellow to red-orange with a high degree of variance. Although disease spots can develop anywhere on the leaf, they tend to concentrate around the edges, where dew and raindrops gather. The center of the spot eventually dries out and turns brown, while edges of the lesions continue to expand and produce new spores. At the beginning of the season, the disease usually first appears on the lower branches, and infection progresses slowly up the tree. Infected leaves fall prematurely, leaving long branches without leaves [14,56,57,58,59].
- Pathogen biology: *Hemileia vastatrix* mainly exists in the form of dikaryotic, and nutrient-absorbing mycelium between cells in leaves of its coffee host. Short pedicels are clustered throughout stomata and below leaves, with dual-nucleated spores. Towards the end of the season, sometimes under cool, dry conditions, spores are produced from polyspores on older attached leaves. After nuclear division and meiosis, these sporozoites germinate to produce basidia, each of which forms four haploid sporozoites [14,56,57,58,59].
- Disease cycle: Urediniosporic life cycle as its most important source of inoculum, can cause infection and develop into lesions, producing more urediniospores. Spore adhesion to the host surface, germination of urediniospores, formation of an adhesion layer on stomata, penetration, and intercellular and intracellular colonization are various steps of the disease cycle. The disease cycle of Coffee Leaf Rust is discussed in Talhinhas et al. [22].
- Disease management:

1. Chemical control: Fungicide sprays (Epoxiconazole, Pyraclostrobin, Cyproconazole, Hexaconazole or Cupric Fungicides) [14,22].
2. Cultural practices: Agroforestry practices of tree-crop mixing, timely pruning, handling and de-suckering, regular change of crop cycle [49].
3. Biological practices: *Pichia membranifaciens* is a yeast strain isolated from soil that can reduce the *Hemileia vastatrix* spore viability [59].
4. Resistant varieties: Such as HDT (Hıbrido de Timor), Catimor and Sarchimor populations [22].

#### 2.3.3. Coffee Berry Disease

- Pathogen: *Colletotrichum kahawae* is a particularly devastating pathogen that affects developing berries, leading to berry rot and shedding before bean formation. *Colletotrichum kahawae* has not been reported outside Africa or in low altitudes. Coffee Berry Disease was first detected and identified by McDonald in Kenya in 1922 [14].
- Hosts: Mainly *C. arabica* (arabica coffee) [14].
- Symptoms and signs: Characteristic symptoms are progressive anthracnose of young and expanding coffee berries. Symptoms present as small water-soaked lesions that rapidly become dark and sunken. These lesions expand, causing rot of the entire berry under humid conditions, and pink spore masses become visible on the lesion surface. Berries are often shed from branches at an early stage of the disease. Lesions may also occur on young berry stalks, causing them to shed before lesions are evident on berries. Pale, corky lesions (scab lesions) also appear on young and mature berries that are resistant to infection. They may completely heal or remain dormant until berries ripen. This disease also affects ripening berries, causing a ‘brown blight’ phase as typical dark, sunken anthracnose lesions that envelop the red berries. *Colletotrichum kahawae* may also infect flowers under wet conditions, causing brown lesions on petals [14,58,60].
- Pathogen biology: The fungus settles in the mature bark of coffee buds and infects flowers, mature fruits, and leaves. Under high humidity and high temperature, conidia germinate and form germ tubes and appendages when contacted with susceptible tissues [61].
- Disease cycle: The Coffee Berry Disease cycle begins each year at the first rain event and is subsequently maintained by rain-splash dispersal and secondary inoculation of conidia onto healthy berries in the rainy season. The disease cycle of Coffee Berry Disease is discussed in De Silva et al. [62].
- Disease management:

1. Cultural practices: Shading with fruit trees and irrigation to induce early flowering to decrease the severity and all berries should be removed at the end of the planting season to prevent them from becoming a source of inoculation for new crops [60].
2. Biological control: Many components in the microbiota (fungi and bacteria) on coffee trees show very high antagonistic levels and have a strong antagonistic effect on *Colletotrichum kahawae*. However, these agents have not been developed into commercial biocontrol agents [60].
3. Chemical control: The most economical method is to use a mixture of copper fungicide (50% wettable copper chloride wettable powder 5 kg) and organic fungicide (75% chlorothalonil wettable powder 2 kg) [14,60].
4. Resistant varieties: Ruiru 11, Hibrido de Timor, Rume Sudan, K7, and several Catimors. In Ethiopia, 37 Coffee Berry Disease resistant coffee cultivars are used [14,60].

#### 2.3.4. Coffee Wilt Disease

- Pathogen: *Fusarium xylarioides* causes wilt in *Coffea excelsa* (*C. liberica*) (Steyaert 1948). This disease was first detected in 1927 in Oubangui-Chari (now the Central African Republic) and was initially thought to be caused by a root rot [14,47].
- Hosts: *C. arabica* (arabica coffee), *C. canephora* (robusta coffee), and *C. excelsa* [47].
- Symptoms and signs: First, leaves turn yellow before withering and developing brown necrotic lesions. Finally, leaves curl, dry, and fall. This process can start from any part of the plant, but eventually, symptoms spread to the rest of the plant. Symptoms first present on the coffee stem, where fungi colonize, and the host response blocks vascular bundles, resulting in blue-black stains [14,47,58,63].
- Pathogen biology: Conidia and ascospores are spread by wind, rain and through human activities (harvesting, pruning). Pathogens penetrate wounds, so any agent causing wounds aids the spread of the fungus. Krantz and Mogk in 1973 noted that most dying and dead trees had been wounded during weeding. Insects may also spread the disease from one tree to another tree [64].
- Disease cycle: Incubation period from first symptoms to death of tree varies, although most affected trees die 2–3 months after initial symptoms were observed. It usually quickly kills infected mature trees within just 6 months after the first external symptoms appear, resulting in a decline of total yield. Coffee quality may also be affected by premature berry ripening. The disease cycle of Coffee Wilt Disease is discussed in Alemu et al. [65].
- Disease management:

1. Cultural practices: Frequent inspection, along with burning infected material and spraying soil surfaces with 2.5% copper (II) sulphate. Replanting should not be done until 6 months after uprooting infected trees to allow the viability of soil inoculum to decline. It is recommended to grow cover crops such as *Desmodium* sp. and haricot bean, which are very efficient in suppressing weeds (so reducing the need for slashing) and as legumes, promote the growth of coffee trees [63].
2. Chemical control: Ridomil Gold (metalxyl 8% + Mancozeb 64%) 68% Wp 2.5 kg/ha, when disease on set, used at 7, 14, 21, 28 days. Pencase 80% WP (Mancozeb) at the rate of 2.5 kg/ha, when disease on set, used at 7, 14, 21 days [63].
3. Biological control: The strain of *Bacillus subtilis* (AUBB20) is the most antagonistic to this disease. *Tricoderma viride* and *Tricoderma harzianum* have shown good potential in inhibiting the mycelial growth of *Fusarium xylarioides*, but no effective methods of biological control are currently available [63].

#### 2.3.5. Brown Eye Spot or Cercospora Blotch

- Pathogen: *Cercospora coffeicola* is distributed throughout the tropics and subtropics and is prone to appear on coffee plants in areas with higher moisture and rainfall as well as on plants that are stressed [14,66].
- Hosts: *C. arabica* (arabica coffee), *C. canephora* (robusta coffee) [66].
- Symptoms and signs: on the leaves, small, round to irregular spots, and brown to light brown lesions first appear. The number and size of lesions then increase before eventually the entire leaf is affected. The edge of the lesion may appear dark purple or black, and it may be encircled by a yellow halo. Severely infected leaves turn yellow and fall off; lesions on green berries are initially brown, sunken, longitudinal, irregular or oval with a gray center. Infection can occur at any stage of berry growth; on the red cherries, first, large, sunken, and blackened areas cover with silvery fungal spores. Penetration into the seeds may cause the pulp to stick to parchment paper during processing, and damage the product. *Cercospora coffeicola* reduces productivity and lowers the beverage quality of coffee [14,57,67].
- Pathogen biology: Wind, splashing water and human activities cause spores (conidia) to be deposited on leaves and petioles, beginning the disease cycle. Conidia germinate at moderate to warm (20–28 °C) temperatures [68].
- Disease cycle: In warm and humid periods, new infections and sporulation occur every 7 to 10 days. Pathogen easily spreads in fields via wind, rain, and irrigation water. It survives as a pathogen in weeds and infested crop fragments, where it is capable of re-infecting grown plants. The disease cycle of *Cercospora* Blotch is discussed in Souza et al. [69].
- Disease management:

1. Biological control: No biological control measures have been developed [68].
2. Cultural practices: Elimination of crop debris, weed hosts and provide 35–65% shade. In order to maintain adequate plant nutrition, nitrogen fertilizers are used. Plant only high-quality seeds, and destroy infected crops in time after the final harvest and before replanting. Select a reasonable planting density (10 ft × 10 ft for robusta while 8 ft × 8 ft for arabica). Avoid planting coffee transplants too deep in soils [68].
3. Chemical control: Fungicide sprays are necessary for disease control in wet conditions, but proper fungicides, rates, and fungicide rotations such as Chlorothalonil and Chlorothalonil Mixtures, Strobilurins and Strobilurin Mixtures should be followed [68].

#### 2.3.6. Armillaria Root Rot

- Pathogen: *Armillaria* root rot is caused by several species of *Armillaria* [14].
- Hosts: *C. arabica* (arabica coffee), *C. canephora* (robusta coffee) [14].
- Symptoms and signs: Common symptoms of *Armillaria* infections include tissue death, wilting and the yellowing of tree-tops, and resin exudation, as well as the underside of bark, is easily attacked by white mycelium [14,58].
- Pathogen biology: This fungus usually exists in soil, and when coffee trees grow in unfavorable conditions, it infects the tree. For example, drought, soil compaction, root injury, and nutrient deficiency may induce it to infect the coffee tree. The fungus produces filaments on the ground, and these filaments can invade healthy roots, move to the root collar, and spread throughout the trunk. The infection causes sapwood in the affected area to rot and eventually kill the tree [70].
- Disease cycle: *Armillaria* spreads in two ways. The first method of transmission is through airborne sexual spores, which can sometimes lead to the creation of new infection centers. The second method of spread is through the growth of pathogens from infected trees to neighboring trees via mycelial transfer at the location where the diseased roots come into contact with each other or via rhizomes that grow through the ground. Depending on the climate, stump size, and other factors, *Armillaria* can live up to 50 years or more. The disease cycle of *Armillaria* root rot is discussed in Jayawardena et al. [71].
- Disease management: The affected trees are incurable. However, if the disease is detected early enough, host trees can be preserved. It is important to prevent this disease by avoiding conditions that cause the tree’s vitality to decline. As *Armillaria* root rot can last for many years, avoid replanting where the diseased trees have been removed [14,58,70].

1. Biological control: Use antagonistic fungi to preemptively settle or eliminate *Armillaria* species in coffee plants.
2. Soil barrier: Creating a barrier in the soil to root and rhizomorph growth may be a practical way to limit the pathogen’s spread, and this is called trenching. This is done by digging a trench down to 1 m (about 3 ft), lining it with plastic, and backfilling.
3. Chemical control: Usually after stump removal and before planting, fumigants such as chloropicrin, carbon disulphide, and methyl bromide are used in orchards to eradicate inocula from the soil.

### 2.4. Distribution of Pathogenic Fungi on Coffee Worldwide

In total, 295 pathogens have been recorded across more than 90 countries (Supplementary Table S1, Figure 1c). The main pathogens are concentrated in Asia and Africa. Brazil has the highest number of pathogens in our statistics.

### 2.5. Pathogenic and Toxigenic Fungi on Coffee

Three pathogenic and two toxigenic fungal genera are reported frequently (Figure 1d). *Aspergillus* and *Penicillium* are the main toxin-producing fungal genera, while *Colletotrichum*, *Hemileia,* and *Fusarium* are the main fungal pathogenic genera on coffee. According to our statistics, *Fusarium* is a pathogen of coffee and also some species in this genus have been reported as mycotoxin producers (Supplementary Table S5) [72].

Twenty-six of the taxa reported on coffee produce toxins viz: 20 *Aspergillus*, two *Penicillium,* and one species from *Byssochlamys*, *Fusarium*, *Mucor,* and *Rhizopus* (Supplementary Table S5). *Fusarium* species are mainly pathogenic affecting coffee causing disease and producing toxins. The main fungal toxins reported on coffee are ochratoxin-A/ochratoxin-B, while Aflatoxin B1/B2 and other mycotoxins were reported less than Ochratoxins [73]. Once the coffee is contaminated with ochratoxin-A, it is difficult to eliminate by cooking due to its thermostability and small size molecules [74]. The accumulation of mycotoxins in plant tissues is related to the development of plant disease symptoms [25]. Based on Supplementary Table S5, the toxin-producing species mainly comes from Aspergillaceae. One hundred and forty-eight records of Aspergillaceae have been reported on coffee worldwide, but there are likely more toxin-producing taxa in this family.

## 3. Discussion and Conclusions

Since the diversity of plant pathogenic fungi is higher than that of plant pathogenic bacteria and viruses, fungal diseases are responsible for the largest coffee losses worldwide [75]. Microbial diseases cause 16% of crop (including coffee) losses worldwide, and 70–80% of these losses are caused by fungi [76]. Cerda et al. [77] showed that pests and diseases cause high primary yield losses (26%) and even higher secondary yield losses (38%). For instance, Coffee berry borer affects coffee yield while increasing the risk of toxigenic fungal infections [18]. 

Fungal infection is a serious problem affecting coffee production and quality [78]. The most serious fungal diseases of coffee reported are Coffee Leaf Rust, Coffee Berry Disease and Coffee Wilt Disease, Brown Eye Spot, and root rot disease [14,58,79]. Coffee Leaf Rust causes loss of physiological activities and leaves to fall off, coffee serves as an obligate host for Coffee Leaf Rust, and this disease devastates susceptible coffee plantations. In severe cases, this disease can cause branches to wither completely, weaken the plant, and hinder or even stop its development; it can cause damage leading to severe yield losses up to 75% [49]. Usually, seriously ill and fragile coffee trees cannot survive Coffee Leaf Rust. Coffee Berry Disease mainly occurs in *C. arabica* and is capable of destroying almost all berries on the coffee tree, but it does not cause any effect on branches and leaves. This disease spreads rapidly above 1500 m altitude under cool and humid conditions and can cause losses as high as 80%. Coffee Wilt Disease is the most serious coffee disease on the African continent. According to Rutherford et al. [47], since 2001, farms in the Democratic Republic of the Congo, Tanzania, and Uganda have suffered from the disease, and the average yield loss was reported at 70%. Brown Eye Spot or *Cercospora* “Berry Blotch” can attack coffee at any stage, and infected plants lose most of their leaves, or even all of them in the same cases, and berries can also be infected. This disease may result in more than 30% production loss and can also reduce quality. *Armillaria* cause rots in various hosts across the world, and coffee is one of the main known hosts. Between 5–20% of all tree deaths can be attributed to this disease in plantations. Some diseases only manifest when plants are accidentally injured by farming tools, especially when wounds are created at the bottom of tree trunks, and in turn, wounds should be treated appropriately with antiseptic healing creams in order to prevent the invasion of diseases such as *Fusarium* wilt and cankers [14,47,80,81]. 

Other less serious pathogens of coffee include Leaf Spot (*Alternaria* sp., and *Ascochyta* sp.) and damping-off (*Pythium* sp.) that are managed by both chemical control and cultural practices [82]; and Coffee Bark Disease which only appears to affect *C. arabica* and can be controlled by adjusting soil pH, nutrient content and maintaining good water management practices [11]. As coffee grows in the tropics and subtropics, leaves remain attached to the tree throughout the year, and it is often covered in microbial endophytes that are important for plant health and plant protection [13,83].

As society pays more attention to food safety and environmental health, it is in our collective interest to reduce the use of pesticides in coffee plantations, and finding beneficial microorganisms and microbial-derived compounds have become a popular and important research field [84]. Fungicides are effective against the disease, but continuous and repeated use of fungicides will promote the emergence of resistant populations, [85]. Moreover, much of the existing literature indicate that fungicides are harmful to the ecosystem, and conventional use will also bring the threat of environmental impact [86]. Additionally, the application of fungicides may affect crop physiology through various disturbances, such as reduced growth, disturbance of the development of reproductive organs, changes in nitrogen and/or carbon metabolism, resulting in reduced nutrient utilization for plant growth [87]. In addition, organic coffee production does not use pesticides. Through the conducted field surveys in coffee plantations in Yunnan Province, China in 2020, we observed that fungal pathogens and pests are still serious factors affecting coffee production and quality.

Different fungi play different roles in the ecosystem. Fungal endophytes and their secondary metabolites may play important roles in the prevention and treatment of coffee diseases. Regarding toxigenic fungi, De Almeida et al. [42] showed that endophytes from coffee beans can inhibit fungal growth and ochratoxin-A production. Regarding Coffee Wilt Disease, the non-pathogenic isolates of *Fusarium* are similar to the pathogenic isolates that cause Coffee Wilt Disease, so they can be studied as effective biocontrol agents. If these non-pathogenic fungal isolates produce secondary metabolites compatible with commonly used fungicides, those secondary metabolites can reduce the severity of fungal diseases [88]. It is known that endophytic fungi can produce metal nanoparticles that have high activity against several microbial pathogens of humans and plants [89]. The direct application of nanoparticles to seeds, leaves or roots can prevent microbial pathogens from invading plants, but the long-term impact of nano-formulations on human health and environmental quality when applied to crop protection is still uncertain [90]. This method of inhibiting pathogens is comparable to that of chemical pesticides, but applications of high amounts of fungicides have led to the emergence of anti-*Plasmodium digitatum* strains [91]. Therefore, nano-formulation is a potential application for controlling diseases. Regarding Coffee Berry Disease, there are almost no chemicals available to control the pathogenic *Colletotrichum* species [92], and its resistance is controlled by three genes, so current research on disease-resistant breeding will likely result in methods to control the disease, but disease-resistant breeding can take up to 30 years [93]. Regarding Coffee Leaf Rust disease is the most serious fungal pathogen responsible for causing leaf diseases [81]. From an environmental and economic point of view, cultivating rust-resistant species is considered the best method. As *Hemileia vastatrix* complex resistance factors and excellent traits cannot be maintained, it has not been studied as extensively as other rust fungi. The resistant varieties in the wild *C*. *arabica* population are worth doing while, there is little hope of success in finding a new source of resistance from wild *C. arabica* [22,94,95]. Jackson et al. [96] showed that the entomopathogenic and mycoparasitic fungus *Lecanicillium lecanii* has potential as an effective biological control against *Hemileia vastatrix* by reducing spore viability and disease severity. Therefore, the most effective method for controlling fungal pathogens on coffee is via the use of endophytic fungi from coffee as bio-controllers.

Mycotoxins on coffee are considered an important food safety issue. Ochratoxins-A is one of the most important mycotoxin pollutants in agricultural products (cereals, wine, coffee, dried fruits, beer, and animal feed). Due to its toxicity and incidence, its harm and impact on humans and animals continue to cause global concern [97]. *Penicillium* and *Aspergillus* species produce ochratoxins and exist in the forms denoted as A, B, and C; ochratoxin-A is the most ubiquitous and most toxic [98]. Culliao et al. [72] isolated *Fusarium*, *Mucor*, and *Rhizopus* from coffee beans that can produce ochratoxin-A. This mycotoxin is usually found in coffee beans and grains, and it cannot be completely removed by food processing methods because of it is light molecular weight and resistance to heat treatments [31,32]. The production of mycotoxins depends on the degree of fungal growth [99]. Coffee berry borer is responsible for increasing fungal contamination and the content of ochratoxin-A [18], while green coffee beans can be contaminated by fungal pathogens in storage [100]. Suárez-Quiroz et al. (2004) showed that different levels of ochratoxin-A are produced during different processing stages; the percentage of infection in green coffee is usually lower than that observed in parchment paper and dry coffee berries, and almost no ochratoxin-A producing fungi are found in green coffee [101]. The Commission of the European Communities has established the internationally acceptable amount or safe amount of ochratoxin-A in food (including coffee) [102]. According to the Food and Agriculture Organization (FAO), 77 countries have formulated guidelines and regulations on mycotoxins in food (including coffee) and feed to control the levels of mycotoxins, but there are still 13 countries (including Africa) that do not have specific regulations for food safety [103].

A range of approaches for ridding mycotoxins in food is currently in the pipeline due to the acute and chronic toxic effects of food contamination by mycotoxins on humans and animals [104], as detailed below:

Regarding biological control, coffee endophytes *Beauveria bassiana* and *Clonostachys rosea* isolated by Vega et al. [36] can control coffee berry borer, the most destructive coffee pest in the world. This plant defense mediated by endophytes can be developed as a new control mechanism. Screening for endophytic fungi that can inhibit the production of ochratoxin-A by post-harvest pathogens and reduce the content of ochratoxin-A in coffee beans should be the future research directions. The use of the non-toxic *Aspergillus niger* strains for the biological control of toxin-producing black aspergilli has also been successfully applied to grapes [20]. The targeted application of biological control methods on coffee plants can potentially increase the quality of coffee beans and ensure the safety of consumers [73]. Application of fungal growth inhibitors to coffee beans, such as the polypeptide bacillomycin D isolated from *Bacillus cereus*, can inhibit the growth of *Fusarium* [105]. 

Regarding physical control, it is known that toxins are produced by a certain level of physical water activity (aw), nutrition, and temperature in micelle. High temperature and humidity are conducive to the production of toxins, all of which are factors controlling the ochratoxin-A production [73]. Various technical methods such as sun drying, infrared and ultrasonic treatments should be used before coffee beans are stored to keep the moisture content below 12%, as this reduces the risk of fungal growth and contaminations [106]. Since molds that produce mycotoxins can usually only colonize damaged parts of plants, crops must be protected from damage caused by mechanical processes or insects. In order to reduce pre-harvest pollution, minimize weeds and reduce the pressure of the crop itself. There can be an appropriate use of fungicide and pesticide treatments [20]. Gil-Serna et al. [107] shows that optimal conditions of growth and produce ochratoxin-A in *Aspegillus steynii* and *A*. *westerdijkiae* can be seen at warm temperatures (28–32 °C). Therefore, coffee bean storage containers should be kept clean in a cool and dry place.

Regarding chemical control, antioxidants are a new strategy that is emerging, such as vanillic acid or 4-hydroxybenzoic acid [108]. Essential oils extracted from plants, such as *Thymus vulgaris, Aframomum danielli* [109,110], cinnamon, clove leaf [111], thyme, marigold, spearmint, basil, quyssum, caraway, anise, oregano, mint, basil, sage, and coriander [112] have been found to inhibit the growth of ochretoxin producing fungi. Additionally, alkaloids produced by Piper longum, and components of sesame oil and turmeric have also been found to suppress both fungal growth and ochratoxin production in a number of ochratoxin-A producing aspergilli [113]. In addition, flavonoids, carotenoids, and saponins can be used as a substitute for synthetic fungicides to control fungal development and ochratoxin-A production in coffee beans [114]. On the other hand, Gómez et al. [115] showed that engineered silver nanoparticles can inhibit the growth of aflatoxigenic and ochratoxigenic fungi. A natural component of rice bran gamma-oryzanol demonstrates antifungal activities against *Fusarium graminearum* [116]. The activated chemicals in cold plasma treatment technology can cause fungal cell damage and death, as well as mycotoxin decomposition. Studies demonstrated that this treatment may eventually provide a sustainable means for processing a large amount of food and animal feed, including coffee [117]. 

To sum up, the use of antagonistic endophytes as biological control agents has attracted special attention for the management of plant diseases and has minimal impact on the environment [118]. Endophytic fungi isolated from coffee, which produce volatile organic compounds, are promising sources of biotechnological potential. The use of microorganisms as biocontrol agents can reduce the huge use of chemicals, and improve the quality and sustainability of coffee, but biocontrol is still not fully utilized in the coffee industry [119]. This comprehensive review of the fungi reported on coffee based on taxonomy, phylogeny, life mode, host, affected plant part and region is useful for mycologists, pathologists, and farmers. Five major fungal diseases and mycotoxin-producing taxa are also discussed in detail in this review. In addition, we discuss why the yield and quality of coffee are affected by fungi and propose methods to control or eliminate infections to increase yield and improve quality. Furthermore, endophytic fungi and their biological control potential for coffee diseases are also discussed.

## 4. Materials and Methods

### 4.1. Fungal Taxa Reported on Coffee

Information on fungal taxa reported on coffee hosts was gathered via Google Scholar, Cyberliber, theses, books, and online literature access sites, and reported fungi were retrieved from the USDA website [120]. In addition, different life modes, hosts, and locations as well as fungi that produce toxins were collected from published articles.

### 4.2. Tables of Coffee Fungi

Fungal species, genera, and higher ranks were checked and updated using Index Fungorum [121] and MycoBank [122]. Five different tables were made in order to clearly understand the different life modes of fungi reported on coffee. All records of coffee fungi are listed in Supplementary Table S1. Frequently found fungal families and genera on coffee are mentioned in Supplementary Table S2. Different life modes of coffee fungi are listed in Supplementary Table S3. True pathogens, postharvest pathogens, and other fungi reported on coffee are mentioned in Supplementary Table S4. Post-harvest fungal diseases reported on coffee are listed in Supplementary Table S5.

### 4.3. Graphs of Coffee Fungi

Microsoft Word’s pie graph option was used to make the Figure 1. Percentages of different fungal families reported on coffee are shown in Figure 1a. Percentages of different life modes of fungi reported on coffee are displayed in Figure 1b. Percentages of coffee pathogens reported in different countries are shown in Figure 1c. Percentages of most frequently reported fungal pathogenic and toxigenic fungal genera on coffee are displayed in Figure 1d.

## Data Availability

Main reference website https://www.ars.usda.gov/ (accessed on 16 February 2022) for the list of coffee fungi.

## Supplementary Materials

The following are available online at https://www.mdpi.com/article/10.3390/pathogens11040411/s1, Table S1: Records of coffee fungi. Table S2: Frequently found fungal families and genera on coffee. Table S3: Different life modes of coffee fungi. Table S4: True pathogens, postharvest pathogens and other fungi reported on coffee. Table S5: Post-harvest fungal diseases reported on coffee. References [123,124,125,126,127,128,129,130,131,132,133,134,135,136,137,138,139,140,141,142,143] are citied in the Supplementary Materials.
