# Supplementary material for: Comprehensive Review of Fungi on Coffee
Source: Pathogens. 2022 Mar 28;11(4):411. doi: 10.3390/pathogens11040411 (PMC9024902; doi:10.3390/pathogens11040411)
Supplement: Supplementary file 1 [file pathogens-11-00411-s001.zip › pathogens-1622050-supplementary.pdf]

Table S1. Records of coffee fungi.

Genera	Species	Host	Substrate	Location	Reference
Acaulosporaceae (1)	*Acaulospora flava*	*Coffea arabica*	Soil	Peru	USDA
Amphisphaeriaceae (11)	*Pestalotia*	*Coffea arabica*	Leaves	Puerto Rico	[1]
	Pestalotia albomaculans	*Coffea liberica*	/	Brazil	USDA
	Pestalotia coffeae	*Coffea arabica*	/	Indonesia, Mexico	USDA
	Pestalotia coffeae	*Coffea robusta*	/	New Caledonia	USDA
	Pestalotia coffeicola	*Coffea abeokutae*	/	Cote d'Ivoire	USDA
	Pestalotia coffeicola	*Coffea arabica*	Plant	Brazil, Taiwan	USDA
	Pestalotia coffeicola	*Coffea liberica*	/	Cote d'Ivoire	USDA
	Pestalotia elasticae	*Coffea arabica*	/	Congo, Democratic Republic	USDA
	Pestalotia sp.	*Coffea arabica*	Plant	Cuba, Mexico	USDA
	Pestalotia sp.	*Coffea liberica*	Plant	Ghana	USDA
	Pestalozzia coffeae – (*Pestalotia coffeae*)	*Coffea liberica*	Plant	Sierra Leone	USDA
Apiosporaceae (4)	*Arthrinium arundinis*	*Coffea arabica*	/	Malawi	USDA
	Arthrinium phaeospermum	*Coffea arabica*	/	Japan	USDA
	Endocalyx melanoxanthus	*Coffea arabica*	Plant	Venezuela	USDA
	Spegazzinia meliolae	*Coffea stenophylla*	Plant	Ghana	USDA
Aspergillaceae (116)	*Aspergillus tamarii*	*Coffea arabica*	beans	Brazil	[2]
	Aspergillus tamarii	*Coffea* sp.	beans	Minas Gerais, Brazil (Cerrado and Sul de Minas)	[3]
	Aspergillus tubingensis	*Coffea* sp.	beans	Minas Gerais, Brazil (Cerrado and Sul de Minas)	[3]
	Aspergillus tubingensis	*Coffea arabica/robusta*	beans	Chiang Mai, Chumphon	[4]
	Aspergillus tubingensis	*Coffea arabica*	beans	Colombia, Kenya	[5]
	Aspergillus aculeatinu	*Coffea arabica/robusta*	beans	Chiang Mai, Chumphon	[6]
	Aspergillus auricomus	*Coffea arabica*	beans	Brazil, Minas Gerais	[7]
	Aspergillus awamori	Arabica parchment coffee /green coffee	beans	Chiang Mai, Thailand	[8]

Aspergillus brasiliensis	*Coffea* sp.	beans	Minas Gerais, Brazil (Cerrado and Sul de Minas)	[3]
Aspergillus caespitosus	*Coffea arabica*	beans	Brazil	[9]
Aspergillus candidus	Arabica parchment coffee/green coffee	beans	Chiang Mai, Thailand	[8]
Aspergillus carbonarius	*Coffea arabica*	beans	Minas Gerais	[7]
Aspergillus carbonarius	*Coffea robusta*	beans	Chiang Mai, Thailand	[4]
Aspergillus carbonarius	Arabica/robusta/liberica/excelsea	beans	Philippines	[10]
Aspergillus elegans	*Coffea arabica*	beans	Brazil	[9]
Aspergillus Flavus	*Coffea arabica*	beans	Saudi Arabia, Brazil	[9,11]
Aspergillus Flavus	*Coffea* sp.	beans	Minas Gerais, Brazil (Cerrado and Sul de Minas)	[3]
Aspergillus flavus	Arabica/robusta/liberica/excelsea	beans	Philippines	[10]
Aspergillus foetidus	*Coffea arabica*	beans	Brazil, Chiang Mai, Chumphon	[9]
Aspergillus fumigatus	*Coffea arabica*	beans	Brazil, India, Viet Nam	[2]
Aspergillus fumigatus	Gorilla's Coffee	coffee powder	Abuja	[12]
Aspergillus fumigatus	*Coffea* sp.	Coffee residue compost	Hiroshima prefecture	[13]
Aspergillus fumigatus	Arabica/robusta/liberica/excelsea	beans	Philippines	[10]
Aspergillus glaucus	*Coffea arabica*	/	Ethiopia	USDA
Aspergillus granulosus	*Coffea arabica*	beans	Brazil	[9]
Aspergillus insulicola	*Coffea arabica*	beans	Brazil, Minas Gerais	[7,9]
Aspergillus japonicus	*Coffea arabica*	beans	Minas Gerais	[7]
Aspergillus japonicus	Arabica/robusta/liberica/excelsea	beans	Philippines	[10]
Aspergillus lanosus	*Coffea* sp.	beans	Minas Gerais, Brazil (Cerrado and Sul de Minas)	[3]
Aspergillus lanosus	*Coffea arabica*	beans	Brazil	[9]
Aspergillus melleus	*Coffea arabica*	beans	Brazil, Minas Gerais, Chiang Mai, Chumphon	[4,7,9,14]
Aspergillus niger	*Coffea arabica*	beans	Brazil, Kenya, Viet Nam, Local markets in Nayarit and Mexico, Lavras, Minas Gerais	[2,15]
Aspergillus niger	*Coffea* sp.	beans	Minas Gerais, Brazil (Cerrado and Sul de Minas), Malaysia	[3]
Aspergillus niger	*Coffea arabica/robusta*	beans	Chiang Mai, Chumphon	[4]
Aspergillus niger	*Coffea canephora*	/	Cote d'Ivoire	USDA
Aspergillus niger	*Coffea abeokutae*	/	Cote d'Ivoire	USDA
Aspergillus niger	*Coffea liberica*	Plant	Cote d'Ivoire, Ghana	USDA
Aspergillus niger	Arabica/robusta/liberica/excelsea	Plant	Philippines	[10]
Aspergillus niger	*Coffea arabica*	beans	Minas Gerais	[7]

aggregate				
Aspergillus niger agregado	*Coffea* sp.	beans	Minas Gerais, Brazil (Cerrado and Sul de Minas)	[3]
Aspergillus niger var. awamor	*Coffea arabica*	beans	Brazil	[9]
Aspergillus niger var. niger	*Coffea arabica*	beans	Brazil	[9]
Aspergillus ochraceus	Arabica parchment coffee/green coffee	beans	Chiang Mai, Thailand	[8]
Aspergillus ochraceus	*Coffea* sp.	beans	Minas Gerais, Brazil (Cerrado and Sul de Minas)	[3]
Aspergillus ochraceus	*Coffea arabica*	beans	Lavras, Minas Gerais, Brazil, Saudi Arabia	[11,14]
Aspergillus ochraceus	Catuai variety	corticous roots tissues	Piumhi, Brazil	[6]
Aspergillus ochraceus	Arabica/robusta/liberica/excelsea	beans	Philippines	[10]
Aspergillus oryzae	*Coffea* sp.	beans	Minas Gerais, Brazil (Cerrado and Sul de Minas)	[3]
Aspergillus oryzae	*Coffea arabica*	Leaves	Chinchiná, Caldas, Colombia	[16]
Aspergillus ostianus	Arabica parchment coffee/green coffee	beans	Chiang Mai, Thailand	[8]
Aspergillus ostianus	*Coffea* sp.	beans	Minas Gerais, Brazil (Cerrado and Sul de Minas)	[3]
Aspergillus ostianus	*Coffea arabica*	beans	Brazil	[9]
Aspergillus parasiticus	Arabica parchment coffee/green coffee	beans	Chiang Mai, Thailand, Saudi Arabia	[8,11]
Aspergillus petrakii	*Coffea arabica*	beans	Brazil	[9]
Aspergillus pseudodeflectus	*Coffea arabica*	beans	Papua New Guinea, Viet Nam	[5]
Aspergillus sclerotiicarbonarius	*Coffea robusta*	beans	Chiang Mai, Chumphon	[4]
Aspergillus sclerotiorum	Arabica parchment coffee/green coffee	beans	Chiang Mai, Thailand, Brazil, Chiang Mai, Chumphon	[8,9]
Aspergillus sclerotiorum	Arabica/robusta/liberica/excelsea	beans	Philippines	[10]
Aspergillus sp. *seção Circumdati*	*Coffea* sp.	beans	Minas Gerais, Brazil (Cerrado and Sul de Minas)	[3]
Aspergillus sp.	*Coffea arabica*	beans	Saudi Arabia, Brazil	[2,11]
Aspergillus steynii	*Coffea arabica*	beans	Chiang Mai, Chumphon	[4]
Aspergillus sulphureus	*Coffea* sp.	beans	Minas Gerais, Brazil (Cerrado and Sul de	[3]

			Minas)	
Aspergillus sulphureus	*Coffea arabica*	beans	Brazil, Minas Gerais	[7]
Aspergillus sumatrense	*Coffea arabica*	beans	Puerto Rico	[5]
Aspergillus sydowii	*Coffea arabica*	beans	Brazil	[9]
Aspergillus terreus	Arabica/robusta/liberica/excelsea	beans	Philippines, Chiang Mai, Thailand	[8,10]
Aspergillus versicolor	*Coffea arabica*	beans/roasted coffee	Local markets in Nayarit and Mexico, Brazil	[15]
Aspergillus westerdijkiae	Arabica/robusta/liberica/excelsea	beans	Thailand, Philippines	[2,4,10]
Byssochlamys Spectabilis	*Coffea arabica*	Roasted coffee	Local markets in Nayarit and Mexico	[15]
Eurotium amstelodami	*Coffea* sp.	beans	Minas Gerais, Brazil (Cerrado and Sul de Minas)	[3]
Eurotium amstelodami	*Coffea arabica*	beans	Brazil	[9]
Eurotium chevalieri	*Coffea arabica*	beans	Brazil	[9]
Eurotium ruber	*Coffea arabica*	beans	India	[5]
Paecilomyces cf. fumosoroseus	*Coffea arabica*	crown	PUERTO RICO	[5]
Paecilomyces cf. javanicus	*Coffea arabica*	Peduncle	COLOMBIA, Caldas, Chinchina,	[5]
Paecilomyces sp.	*Coffea arabica*	Epicarp, seedling, Root	MEXICO, Chiapas, Cacahoatan, Rancho El Paraı so, USA. Hawaii, Kona Experimental Station	[5]
Penicillium coffeae	*Coffea arabica*	plant	Hawaii	USDA
Penicillium crustosum	*Coffea arabica*	beans	Guatemala	[5]
Penicillium digitatum	*Coffea arabica*	plant	Brazil	USDA
Penicillium digitatum	Arabica/robusta/liberica/excelsea	beans	Philippines	[10]
Penicillium olsonii	*Coffea arabica*	beans	Colombia	[5]
Penicillium olsonii	*Coffea congensis*	plant	Hawaii	[5]
Penicillium olsonii	*Coffea dewevrei*	plant	Hawaii	[5]
Penicillium olsonii	*Coffea liberica*	plant	Hawaii	[5]
Penicillium brevicompactum	*Coffea* sp.	beans	Minas Gerais, Brazil (Cerrado and Sul de Minas)	[3]
Penicillium chrysogenum	*Coffea arabica*	beans	Saudi Arabia, Brazil	[9,11]
Penicillium citrinum	*Coffea* sp.	beans	Minas Gerais, Brazil (Cerrado and Sul de Minas)	[3]
Penicillium citrinum	*Coffea arabica*	beans	Brazil	[9]
Penicillium citrinum	Arabica/robusta/liberica/excelsea	beans	Philippines	[10]
Penicillium commune	*Coffea* sp.	beans	Minas Gerais, Brazil (Cerrado and Sul de Minas)	[3]
Penicillium corylophylum	*Coffea arabica*	beans	Saudi Arabia	[11]

	Penicillium corylophylum	Arabica/robusta/liberica/excelsea	beans	Philippines	[10]
	Penicillium funiculosum	*Coffea arabica*	beans	Lavras, Minas Gerais.	[14]
	Penicillium italicum	*Coffea* sp.	beans	Minas Gerais, Brazil (Cerrado and Sul de Minas)	[3]
	Penicillium glabrum	*Coffea arabica*	beans	Brazil	[9]
	Penicillium Rugulosum	*Coffea arabica*	beans	Lavras, Minas Gerais.	[14]
	Penicillium verruculosum	*Coffea* sp.	coffee residue compost	Hiroshima prefecture	[13]
	Penicillium verruculosum	Arabica/robusta/liberica/excelsea	beans	Philippines	[10]
	Penicillium ubiquetum	*Coffea arabica*	Coffee soil	Colombian andisols	[17]
	Penicillium Variable	*Coffea arabica*	beans	Lavras, Minas Gerais.	[14]
	Penicillium aurantiogriseum	*Coffea arabica*	beans	Brazil	[9]
	Penicillium brevecompactum	*Coffea arabica*	beans	Brazil	[9]
	Penicillium corylophilum	*Coffea arabica*	beans	Brazil	[9]
	Penicillium expansum	*Coffea arabica*	beans	Brazil	[9]
	Penicillium jantinelum	*Coffea arabica*	Coffee soil	Colombian andisols	[17]
	Penicillium solitum	*Coffea arabica*	beans	Brazil	[9]
	Penicillium sp.	*Coffea arabica*	beans, coffee soil	Brazil, Saudi Arabia, India, Colombian andisols	USDA, [5,11,17]
	Penicillium sp.	Catuai variety	corticous root tissues	Brazil, Piumhi	[6]
	Aspergillus alliaceus	Arabica/robusta/liberica/excelsea	beans	Philippines	[10]
	Aspergillus clavatus	Arabica/robusta/liberica/excelsea	beans	Philippines	[10]
	Penicillium implicatum	Arabica/robusta/liberica/excelsea	beans	Philippines	[10]
	Penicillium montanense	Arabica/robusta/liberica/excelsea	beans	Philippines	[10]
	Penicillium decumbens	Arabica/robusta/liberica/excelsea	beans	Philippines	[10]
	Penicillium pelutatum	Arabica/robusta/liberica/excelsea	beans	Philippines	[10]
	Penicillium oxalicum	Arabica/robusta/liberica/excelsea	beans	Philippines	[10]
	Penicillium waksmanii	Arabica/robusta/liberica/excelsea	beans	Philippines	[10]
Asterinaceae (2)	*Asterina coffeicola*	*Coffea arabica*	/	Jamaica	USDA
	Dimerosporium coronatum	*Coffea arabica*	/	Costa Rica	USDA
Atheliaceae (1)	*Athelia rolfsii*	*Coffea arabica*	leaf	Brazil	USDA
Auriculariaceae (3)	*Auricularia delicata* – (*Laschia delicata*)	*Coffea arabica*	/	Puerto Rico, Virgin Islands	USDA
	Auricularia polytricha –	*Coffea arabica*	/	Philippines	USDA

	(Auricularia cornea)				
	Hirneola polytricha – *(Auricularia cornea)*	*Coffea arabica*	plant	Cuba	USDA
Beltraniaceae (1)	*Beltrania rhombica*	*Coffea arabica*	plant	Venezuela	USDA
Bionectriaceae (1)	*Clonostachys rosea*	*Coffea arabica*	Leaves	COLOMBIA, Caldas, Chinchina	[5]
Boliniaceae (1)	*Ceratostomella fimbriata* – *(Ceratocystis fimbriata)*	*Coffea arabica*	plant	Mexico	USDA
Botryosphaeriaceae (17)	*Botryosphaeria* sp.	*Coffea arabica*	Leaves	Puerto Rico	[1]
	Diplodia coffeicola	*Coffea abeokutae*	/	Cote d'Ivoire	USDA
	Diplodia coffeicola	*Coffea arabica*	plant	Brazil	USDA
	Diplodia sp.	*Coffea* sp., *Coffea arabica*	Leaves	Mexico, Papua New Guinea	USDA
	Diplodina sp.	*Coffea* sp.	/	Haiti	USDA
	Fusicoccum sp.	*Coffea arabica*	plant	Mexico	USDA
	Lasiodiplodia pseudotheobromae	*Coffea arabica*	plant	Brazil, Thailand	USDA
	Lasiodiplodia pseudotheobromae	*Coffea* sp.	plant	Congo, Democratic Republic	USDA
	Lasiodiplodia theobromae	*Coffea arabica*	plant	Cuba, Fiji, Venezuela	USDA
	Lasiodiplodia theobromae	*Coffea canephora*	plant	Fiji	USDA
	Macrophoma coffeae var. macrospora	*Coffea arabica*	plant	Brazil	USDA
	Macrophoma corchori – *(Macrophomina phaseolina)*	*Coffea arabica*	plant	Thailand	USDA
	Macrophomina phaseoli – *(Macrophomina phaseolina)*	*Coffea arabica*	plant	Kenya, Tanzania, Thailand, Uganda	USDA
	Macrophomina phaseoli – *(Macrophomina phaseolina)*	*Coffea canephora var. robusta*	plant	Sri Lanka, Uganda	USDA
	Macrophomina phaseolina	*Coffea arabica*	root	Kenya	USDA
	Microdiplodia sp.	*Coffea robusta*	plant	Malaysia	USDA
	Neofusicoccum parvum	*Coffea arabica*	/	Colombia	USDA
Capnodiaceae (24)	*Aithaloderma longisetum*	*Coffea* sp.	plant	Philippines	USDA
	Aithaloderma longisetum	*Coffea arabica*	/	Belgium, Colombia	USDA
	Aithaloderma setosum	*Coffea* sp.	plant	Indonesia	USDA

	Aithaloderma setosum	*Coffea arabica*	/	Belgium	USDA
	Aithaloderma setosum	*Coffea excelsa*	/	Philippines	USDA
	Aithaloderma setosum	*Coffea liberica*	plant	Brunei Darussalam, Philippines	USDA
	Aithaloderma setosum	*Coffea robusta*	plant	Brunei Darussalam	USDA
	Capnodium brasiliense	*Coffea arabica*	plant	Brazil, Kenya	USDA
	Capnodium brasiliense	*Coffea liberica*	plant	Ghana	USDA
	Capnodium brasiliense	*Coffea* sp.	/	Brazil, Colombia	USDA
	Capnodium citri – (*Aithaloderma citri*)	*Coffea arabica*	plant	Brazil	USDA
	Capnodium coffeae	*Coffea arabica*	plant	Costa Rica, El Salvador, Guatemala, Honduras, Mexico, Nicaragua, Panama	USDA
	Capnodium coffeae	*Coffea robusta*	plant	Congo Democratic Republic	USDA
	Capnodium coffeicola	*Coffea* sp.	leaves	Chiang Rai Thailand	[18]
	Capnodium sp.	*Coffea arabica*	plant	Cuba, Haiti, Mexico, Panama, Sudan, Tanzania, Zimbabwe	USDA
	Capnodium sp.	*Coffea cassia*	plant	Cuba	USDA
	Capnodium sp.	*Coffea* sp.	plant	Fiji, Hawaii	USDA
	Limacinia sp.	*Coffea arabica*	plant	Mexico	USDA
	Paracapnodium brasilense	*Coffea arabica*	leaves	Colombia	USDA
	Paraconiothyrium brasiliense	*Coffea arabica*	fruit	Brazil	USDA
	Scorias communis	*Coffea liberica*	plant	China, Taiwan	USDA
	Scorias sp.	*Coffea canephora*	/	Papua New Guinea	USDA
	Tripospermum gardneri	*Coffea arabica*	plant	Malay Peninsula	USDA
	Tripospermum gardneri	*Coffea liberica*	plant	Malay Peninsula	USDA
Caryosporaceae (1)	*Caryospora coffeae*	*Coffea* sp.	/	Venezuela	USDA
Ceratobasidiaceae (39)	*Koleroga noxia* – (*Rhizoctonia noxia*)	*Coffea arabica*	plant	Cuba, Papua New Guinea	USDA
	Koleroga noxia – (*Rhizoctonia noxia*)	*Coffea canephora*	/	Papua New Guinea	USDA
	Koleroga noxia – (*Rhizoctonia noxia*)	*Coffea* sp.	/	Papua New Guinea	USDA
	Pellicularia filamentosa – (*Rhizoctonia solani*)	*Coffea arabica*	roots, stems	El Salvador, Jamaica, Tanzania	USDA
	Pellicularia isabellina – (*Botryohypochnus isabellinus*)	*Coffea arabica*	plant	Brazil	USDA

Pellicularia koleroga – (*Rhizoctonia noxia*)	*Coffea abeokutae*	/	Puerto Rico, Virgin Islands	USDA
Pellicularia koleroga – (*Rhizoctonia noxia*)	*Coffea arabica*	branches, leaf	Bolivia, Brazil, Colombia, Costa Rica, Cote d'Ivoire, Cuba, Dominican Republic	USDA
Pellicularia koleroga – (*Rhizoctonia noxia*)	*Coffea canephora*	plant	Fiji	USDA
Pellicularia koleroga – (*Rhizoctonia noxia*)	*Coffea* sp.	plant	Samoa	USDA
Pellicularia koleroga – (*Rhizoctonia noxia*)	*Coffea stenophylla*	/	Puerto Rico, Virgin Islands	USDA
Pellicularia sp.	*Coffea arabica*	plant	Cambodia	USDA
Rhizoctonia bataticola – (*Macrophomina phaseolina*)	*Coffea arabica*	plant	Uganda	USDA
Rhizoctonia bataticola – (*Macrophomina phaseolina*)	*Coffea robusta*	plant	Sri Lanka, Uganda	USDA
Rhizoctonia choussii	*Coffea arabica*	plant	Cuba, El Salvador	USDA
Rhizoctonia lamellifera – (*Macrophomina phaseolina*)	*Coffea arabica*	/	Kenya	USDA
Rhizoctonia solani	*Coffea arabica*	seedlings	Brazil, China, Cuba, Florida, Mexico, Zimbabwe	USDA
Rhizoctonia solani	*Coffea liberica*	plant	Cuba	USDA
Rhizoctonia sp.	*Coffea arabica*	seedlings, plant	China, Costa Rica, El Salvador, Haiti, Mexico, Panama	USDA
Rhizoctonia sp.	*Coffea canephora*	plant	Fiji	USDA
Rhizoctonia sp.	*Coffea* sp.	plant	Malawi, Philippines	USDA
Thanatephorus cucumeris – (*Rhizoctonia solani*)	*Coffea arabica*	plant	Taiwan	USDA
Thanatephorus cucumeris – (*Rhizoctonia solani*)	*Coffea* sp.	/	Papua New Guinea	USDA
Ambrosiella xylebori	*Coffea canephora*	/	Africa, Cote d'Ivoire, India, Sri Lanka, Taiwan, United States	USDA
Ceratocystis colombiana	*Coffea arabica*	wood	Colombia	USDA
Ceratocystis colombiana	*Coffea* sp.	wood	Colombia	USDA

	Ceratocystis fimbriata	*Coffea arabica*	plant	Brazil, Colombia, Costa Rica, El Salvador, Guatemala, Nicaragua, Panama, Puerto Rico, Venezuela, Virgin Islands	USDA
	Ceratocystis fimbriata	*Coffea arabica*	trunks, branches	Bourbon Guatemala	USDA
	Ceratocystis fimbriata	*Coffea* sp.	soil, plant	Colombia	USDA
	Ceratocystis papillata	*Coffea arabica*	plant	Colombia	USDA
	Ceratocystis papillata	*Coffea* sp.	wood	Colombia	USDA
	Ceratocystis paradoxa – (*Thielaviopsis paradoxa*)	*Coffea abeokutae*	leaves, stem	Cote d'Ivoire	USDA
	Ceratocystis paradoxa – (*Thielaviopsis paradoxa*)	*Coffea canephora*	leaves, stem	Cote d'Ivoire	USDA
	Ceratocystis paradoxa – (*Thielaviopsis paradoxa*)	*Coffea liberica*	leaves, stem	Cote d'Ivoire	USDA
	Davidsoniella neocaledoniae	*Coffea robusta*	/	New Caledonia	USDA
	Thielaviopsis neocaledoniae – (*Davidsoniella neocaledoniae*)	*Coffea robusta*	wood	United States	USDA
	Thielaviopsis paradoxa	*Coffea abeokutae*	/	Cote d'Ivoire	USDA
	Thielaviopsis paradoxa	*Coffea arabica*	plant	Brazil	USDA
	Thielaviopsis paradoxa	*Coffea canephora*	/	Cote d'Ivoire	USDA
	Thielaviopsis paradoxa	*Coffea liberica*	/	Cote d'Ivoire	USDA
Chaetomiaceae (1)	*Chaetomium globosum*	*Coffea* sp.	beans	Minas Gerais, Brazil (Cerrado and Sul de Minas)	[3]
Chaetosphaeriaceae (1)	*Melanochaeta hemipsila* – (*Sporoschisma hemipsilum*)	*Coffea robusta*	/	Central African Republic	USDA
Chaetothyriaceae (13)	*Ceramothyrium coffeanum*	*Coffea robusta*	/	New Guinea	USDA
	Ceratobasidium noxium – (*Rhizoctonia noxia*)	*Coffea arabica*	leaves	India	USDA
	Ceratobasidium noxium – (*Rhizoctonia noxia*)	*Coffea* sp.	/	Colombia, India, Puerto Rico, Trinidad and Tobago	USDA
	Ceratobasidium sp.	*Coffea arabica*	plant	Fiji	USDA
	Ceratobasidium sp.	*Coffea canephora*	plant	Fiji	USDA

	Ceratobasidium sp.	*Coffea* sp.	plant	Fiji, Samoa	USDA
	Chaetothyrium boedijnii	*Coffea arabica*	/	Papua New Guinea	USDA
	Chaetothyrium setosum	*Coffea arabica*	/	Colombia	USDA
	Phaeosaccardinula coffeicola	*Coffea arabica*	leaves	Chiang Mai Thailand.	[19]
	Phaeosaccardinula javanica – (*Limacinula javanica*)	*Coffea arabica*	plant	China, Taiwan	USDA
	Phaeosaccardinula javanica – (*Limacinula javanica*)	*Coffea liberica*	plant	China, Taiwan	USDA
	Phaeosaccardinula javanica – (*Limacinula javanica*)	*Coffea robusta*	plant	China, Taiwan	USDA
	Phaeosaccardinula sp.	*Coffea arabica*	/	Eritrea	USDA
Chionosphaeraceae (2)	*Stilbum flavidum* – (*Mycena citricolor*)	*Coffea arabica*	plant	Mexico	USDA
	Stilbum flavidum – (*Mycena citricolor*)	*Coffea* sp.	/	Venezuela	USDA
Choanephoraceae (2)	*Chalara neocaledoniae* – (*Davidsoniella neocaledoniae*)	*Coffea robusta*	leaf	New Caledonia	USDA
	Choanephora conjuncta – (*Choanephora infundibulifera*)	*Coffea arabica*	plant	Japan	USDA
Cladosporiaceae (17)	*Cladosporium cf. sphaerospermum*	*Coffea arabica*	leaves	USA, Maryland, Beltsville	[5]
	Cladosporium sp.	*Coffea arabica*	plant, peduncle, beans	Australia, Brazil, Cuba, USA, Hawaii, Kona, Lavras, Minas Gerais.	USDA, [5,14]
	Cladosporium sp.	*Coffea aurantii*	/	Cyprus	USDA
	Cladosporium sp.	*Coffea robusta*	plant	Malawi	USDA
	Cladosporium sp.	*Coffea* sp.	beans	Minas Gerais, Brazil (Cerrado and Sul de Minas)	[3]
	Cladosporium sp.	*Coffea arabica*	leaves	Brazil	[20]
	Cladosporium cf.cladosporioides	*Coffea arabica*	leaves	Puerto Rico, USA, Hawaii, Kona	[5]

	Cladosporium cladosporioides	*Coffea arabica*	leaves	Papua New Guinea	USDA
	Cladosporium cladosporioides	*Coffea* sp.	leaf	Uganda	USDA
	Cladosporium hemileiae	*Coffea robusta*	/	Congo, Democratic Republic	USDA
	Cladosporium herbarum	*Coffea arabica*	/	Mozambique	USDA
	Cladosporium herbarum	*Coffea canephora var. robusta*	leaves, young seedlings	Indonesia	USDA
	Cladosporium herbarum	*Coffea* sp.	/	Southern Africa	USDA
	Cladosporium oxysporum	*Coffea robusta*	plant	Brunei Darussalam	USDA
	Cladosporium cladosporioides complex	*Coffea arabica*	Mature and healthy leaves	Brazil	[21]
	Cladosporium tenuissimum	*Coffea arabica*	Mature and healthy leaves	Brazil	[21]
	Cladosporium pseudocladosporioides	*Coffea* sp.	plant	Uganda, United States	USDA
Clavicipitaceae (6)	*Aschersonia coffeae* – (*Moelleriella javanica*)	*Coffea abeokutae*	/	Cote d'Ivoire	USDA
	Aschersonia sp.	*Coffea arabica*	/	Papua New Guinea	USDA
	Aschersonia sp.	*Coffea robusta*	plant	Malaysia	USDA
	Clavicipitaceae sp.	*Coffea arabica*	green coffee seeds	Puerto Rico	[5]
	Hypocrella scutata – (*Hypocrella olivacea*)	*Coffea canephora*	/	Papua New Guinea	USDA
	Hypocrella sp.	*Coffea arabica*	/	Papua New Guinea	USDA
Coccodiniaceae (5)	*Deslandesia ficina*	*Coffea liberica*	/	Indonesia	USDA
	Deslandesia javanica – (*Limacinula javanica*)	*Coffea* sp.	/	Indonesia	USDA
	Microxiphium coffeanum	*Coffea arabica*	plant	Brazil	USDA
	Microxyphium sp.	*Coffea arabica*	plant	Cuba, Sudan	USDA
	Microxyphium sp.	*Coffea canephora*	/	Papua New Guinea	USDA
Coniochaetaceae (2)	*Coniochaeta ligniaria*	*Coffea* sp.	Coffee residue compost	Hiroshima prefecture	[13]
	Coniochaeta velutina	*Coffea* sp.	Coffee residue compost	Hiroshima prefecture	[13]
Coniothyriaceae (7)	*Coniothyrium coffeae*	*Coffea arabica*	/	Brazil, Haiti, Philippines	USDA
	Coniothyrium coffeae	*Coffea liberica*	/	India	USDA
	Coniothyrium coffeae	*Coffea robusta*	plant	Brunei Darussalam	USDA
	Coniothyrium coffeae	*Coffea* sp.	plant	Philippines	USDA
	Coniothyrium fuckelii –	*Coffea arabica*	plant	Venezuela	USDA

	(Paraconiothyrium fuckelii)				
	Coniothyrium sp.	*Coffea liberica*	plant	Malaysia	USDA
	Coniothyrium sp.	*Coffea robusta*	plant	Malaysia	USDA
Coniosporiaceae (1)	*Coniosporium sp.*	*Coffea arabica*	leaves	Mexico	[22]
Cordycipitaceae (6)	*Akanthomyces johnsonii*	*Coffea* sp.	decaying leaves	Ohio	USDA
	Beauveria bassiana	*Coffea arabica*	green coffee fruits	Puerto Rico	[22]
	Beauveria bassiana	*Coffea arabica*	Seed, Epicarp, Peduncle, Crown	Colombia, Caldas, Chinchina	[5]
	Beauveria brongniartii	*Coffea arabica*	leaves	Mexico	[22]
	Beauveria globulifera	*Coffea arabica*	/	Puerto Rico, Virgin Islands	USDA
	Simplicillium sp.	*Coffea arabica*	stem	Viçosa municipality, Minas Gerais, Brazil	[23]
Corticiaceae (9)	*Corticium koleroga – (Rhizoctonia noxia)*	*Coffea abeokutae*	/	Cote d'Ivoire	USDA
	Corticium koleroga –(Rhizoctonia noxia)	*Coffea arabica*	plant	Cuba, Dominican Republic, Jamaica, Mexico, Panama, Trinidad, Tobago, Venezuela	USDA
	Corticium koleroga –(Rhizoctonia noxia)	*Coffea liberica*	/	Cote d'Ivoire	USDA
	Corticium salmonicolor–(Erythricium salmonicolor)	*Coffea arabica*	plant	CostaRica, El Salvador, Guatemala, Honduras, Kenya, Mexico, Nicaragua, Panama, Puerto Rico, Taiwan, Virgin Islands	USDA
	Corticium salmonicolor –(Erythricium salmonicolor)	*Coffea canephora var. robusta*	/	Guinea	USDA
	Corticium salmonicolor –(Erythricium salmonicolor)	*Coffea* sp.	plant	Malay Peninsula	USDA
	Corticium solani – (Rhizoctonia solani)	*Coffea arabica*	plant	Jamaica, Kenya, Malay Peninsula, Malaysia, Tanzania	USDA
	Corticium sp.	*Coffea arabica*	/	Papua New Guinea	USDA
	Erythricium salmonicolor	*Coffea arabica*	plant	Brazil, Sri Lanka	USDA
Corynesporascaceae (1)	*Corynespora cassiicola*	*Coffea canephora*	leaf, berry	Brazil	[24]
Cucurbitariaceae (2)	*Pyrenochaetopsis microspora*	*Coffea arabica*	leaf	Brazil	USDA
	Pyrenochaetopsis setosissima	*Coffea arabica*	leaf	Brazil	USDA
Dacampiaceae (1)	*Aaosphaeria arxii*	*Coffea excelsa*	/	Central African Republic	USDA

Dacrymycetaceae (2)	*Dacryopinax spathularia*	*Coffea arabica*	plant	Venezuela	USDA.
	Dactylaria haptospora	*Coffea arabica*	/	Australia	USDA
Diaporthaceae (11)	*Diaporthe acutispora*	*Coffea* sp.	/	China	USDA
	Diaporthe sp.	*Coffea* sp.	leaves	China	USDA
	Diaporthe sp.	*Coffea arabica*	/	Thailand	USDA
	Diaporthe liquidambaris	*Coffea arabica*	Mature and healthy leaves	Brazil	[21]
	Diaporthe phaseoli	*Coffea arabica*	Mature and healthy leaves	Brazil	[21]
	Diaporthe yunnanensis	*Coffea* sp.	healthy leaves	China	USDA
	Phomopsis arnoldiae	*Coffea arabica*	leaves	Mexico	[22]
	Phomopsis coffeae	*Coffea arabica*	/	India	USDA
	Phomopsis heveicola	*Coffea arabica*	leaves	China	USDA
	Phomopsis sp.	*Coffea arabica*	leaves	Florida, Hawaii, Mexico,Tanzania, Venezuela	USDA
	Phomopsis stipata	*Coffea arabica*	leaves	/	[25]
Diatrypaceae (5)	*Anthostoma* sp.	*Coffea robusta*	plant	Brunei Darussalam	USDA
	Eutypella coffeicola	*Coffea canephora*	/	Cote d'Ivoire	USDA
	Eutypella coffeicola	*Coffea liberica*	/	Cote d'Ivoire	USDA
	Peroneutypa multistromata	*Coffea canephora*	/	Cote d'Ivoire	USDA
	Libertella sp.	*Coffea arabica*	leaves	/	[25]
Didymellaceae (34)	*Allophoma nicaraguensis*	*Coffea* sp.	twig	Nicaragua	USDA
	Allophoma nicaraguensis	*Coffea arabica*	/	Nicaragua	USDA
	Ascochyta coffeae – (*Boeremia exigua var. coffeae*)	*Coffea arabica*	leaves	Brazil	USDA
	Ascochyta sp.	*Coffea arabica*	plant	Brazil, Eritrea	USDA
	Ascochyta sp.	*Coffea canephora var. robusta*	/	Guinea	USDA.
	Ascochyta sp.	*Coffea robusta*	plant	Brunei Darussalam	USDA
	Ascochyta tarda (*Boeremia exigua var. coffeae*)	*Coffea arabica*	leaves, branches	Ethiopia, Malawi	USDA
	Boeremia exigua var. coffeae	*Coffea arabica*	leaves	Brazil, Cameroon	USDA
	Boeremia exigua var. heteromorpha	*Coffea arabica*	/	Brazil	USDA
	Didymella coffeae-arabicae	*Coffea arabica*	/	Ethiopia	USDA
	Didymosphaeria sp.	*Coffea canephora*	/	Papua New Guinea	USDA

	Didymosphaeria sp.	*Coffea robusta*	plant	Malaysia	USDA
	Didymostilbe coffeae	*Coffea arabica*	/	Indonesia	USDA
	Didymostilbe coffeae	*Coffea* sp.	/	Mexico Texas	USDA
	Epicoccum sorghi – (*Epicoccum sorghinum*)	*Coffea* sp.	fruit	India	USDA
	Peyronellaea coffea-arabicae (*Didymella coffeae-arabicae*)	*Coffea arabica*	/	Ethiopia	USDA
	Phoma coffeae	*Coffea arabica*	/	China	USDA
	Phoma coffeae-arabicae – (*Didymella coffeae-arabicae*)	*Coffea arabica*	/	Ethiopia	USDA
	Phoma coffeicola	*Coffea abeokuta*	/	Cote d'Ivoire	USDA
	Phoma coffeicola	*Coffea canephora*	/	Cote d'Ivoire	USDA
	Phoma coffeicola	*Coffea liberica*	/	Cote d'Ivoire	USDA
	Phoma costaricensis	*Coffea arabica*	plant	Costa Rica, Guatemala, Papua New Guinea, Venezuela	USDA
	Phoma costaricensis	*Coffea robusta*	plant	Brunei Darussalam	USDA
	Phoma costarricensis (*Phoma costaricensis*)	*Coffea arabica*	plant	Brazil, Costa Rica, India, Mexico, New Guinea, Nicaragua	USDA
	Phoma costarricensis (*Phoma costaricensis*)	*Coffea* sp.	/	Nicaragua	USDA
	Phoma leveillei	*Coffea arabica*	/	Papua New Guinea	USDA
	Phoma pereupyrena (*Ectophoma pomi*)	*Coffea arabica*	leaf	India	USDA
	Phoma sorghina (*Epicoccum sorghinum*)	*Coffea* sp.	fruit	India	USDA
	Phoma sp.	*Coffea arabica*	plant	Brazil, Cuba, India	USDA
	Phoma sp.	*Coffea canephora*	plant	Brazil	USDA
	Phoma eupyrena	*Coffea arabica*	Mature and healthy leaves	Brazil	[21]
	Phoma tarda – (*Boeremia exigua var. coffeae*)	*Coffea arabica*	coffee berry	Brazil, Cameroon, Ethiopia, Kenya	USDA
	Phoma herbarum	*Coffea arabica*	leaves	/	[25]
	Phoma exigua var. exigua	*Coffea arabica*	leaves	/	[25]
Didymosphaeriaceae (4)	*Munkovalsaria donacina*	*Coffea arabica*	/	Paraguay	USDA
	Munkovalsaria donacina	*Coffea robusta*	/	Central African Republic	USDA

	Phaeodothis winteri	*Coffea arabica*	leaves	Brazil, India	USDA
	Phaeodothis winteri	*Coffea robusta*	leaves	Malaysia	USDA
Dipodascaceae (1)	*Geotrichum candidum*	*Coffea* sp.	seed	Papua New Guinea	USDA
Dothideaceae (3)	*Auerswaldia excoriata*	*Coffea abeokutae*	/	Cote d'Ivoire	USDA
	Auerswaldia excoriata	*Coffea canephora*	/	Cote d'Ivoire	USDA
	Auerswaldia excoriata	*Coffea liberica*	/	Cote d'Ivoire	USDA
Drepanopezizaceae (6)	*Gloeosporium coffeanum*	*Coffea arabica*	/	Kenya	USDA
	Gloeosporium coffeicola	*Coffea arabica*	plant	Taiwan	USDA
	Gloeosporium coffeicola	*Coffea* sp.	/	Samoa	USDA
	Gloeosporium coffeicola var. ramulicola	*Coffea arabica*	/	China	USDA
	Gloeosporium sp.	*Coffea arabica*	plant	Mexico	USDA
	Gloeosporium sp.	*Coffea* sp.	/	Hawaii	USDA
Entomophthoraceae (1)	*Entomophthora fresenii*	*Coffea arabica*	/	India	USDA
Eremotheciaceae (3)	*Nematospora coryli* – (*Eremothecium coryli*)	*Coffea arabica*	plant	Kenya, Tanzania	USDA
	Nematospora coryli – (*Eremothecium coryli*)	*Coffea* sp.	plant	Malawi	USDA
	Nematospora gossypii – (*Eremothecium gossypii*)	*Coffea arabica*	/	Kenya	USDA
Erysiphaceae (1)	*Sphaerotheca coffeaeicola*	*Coffea* sp.	/	Paraguay	USDA
Gigasporaceae (1)	*Gigaspora margarita*	*Coffea arabica*	plant	Brazil	USDA
Glomerellaceae (77)	*Colletotrichum* sp.	*Coffea arabica*	leaves	Brazil, Puerto Rico	[1,20]
	Colletotrichum acutatum	*Coffea arabica*	coffee berries	Thailand Chiang Mai, Kenya, Papua New Guinea, Viet Nam	[26]
	Colletotrichum acutatum	Cape Australia Coffee Estate	coffee berries	Papua New Guinea	[26]
	Colletotrichum boninense	*Coffea arabica*	leaves and berries	Brazil, Viet Nam	[27]
	Colletotrichum boninense	*Coffea canephora*	leaves and berries	Brazil	USDA
	Colletotrichum brevisporum	*Coffea canephora var. robusta*	leaves and berries	China	USDA
	Colletotrichum capsici – (*Colletotrichum truncatum*)	*Coffea arabica*	green coffee fruits	Florida, Venezuela, Viet Nam	USDA, [27]
	Colletotrichum capsici – (*Colletotrichum truncatum*)	*Coffea liberica*	plant	Brunei Darussalam	USDA

Colletotrichum cereale	*Coffea arabica*	coffee berries	Eritrea	USDA
Colletotrichum coffeanum	*Coffea abeokutae*	/	Cote d'Ivoire	USDA
Colletotrichum coffeanum	*Coffea arabica*	leaves, green coffee fruits	Brazil, Cameroon, China, Congo, Democratic Republic, Costa Rica, Cuba, Dominican Republic, East Africa, El Salvador, Eritrea, Ethiopia, Florida, Guatemala, Honduras, Jamaica, Japan, Kenya, Malay Peninsula, Mexico, Myanmar, Nicaragua, Panama, Range of host, Tanzania	USDA, [27]
Colletotrichum coffeanum	*Coffea canephora*	/	Cote d'Ivoire	USDA
Colletotrichum coffeanum	*Coffea liberica*	/	China, Cote d'Ivoire	USDA
Colletotrichum coffeanum	*Coffea robusta*	/	China	USDA
Colletotrichum coffeanum	*Coffea* sp.	plant	Ghana, Malay Peninsula, Mauritius, Nigeria, Southern Africa	USDA
Colletotrichum coffeophilum	*Coffea arabica*	leaves	China, Costa Rica, Taiwan	USDA
Colletotrichum costaricense	*Coffea arabica*	coffee berries	Costa Rica	USDA
Colletotrichum costaricense	*Coffea* sp.	coffee berries	Costa Rica	USDA
Colletotrichum dematium	*Coffea arabica*	/	Kenya	USDA
Colletotrichum endophyticum	*Coffea canephora var. robusta*	leaves and berries	China	USDA
Colletotrichum fragariae – (*Colletotrichum theobromicola*)	*Coffea* sp.	/	Angola, Brazil	USDA
Colletotrichum fructicola	*Coffea arabica*	leaves and berries	China, Puerto Rico, Thailand	USDA, [26,27]
Colletotrichum fructicola	*Coffea canephora var. robusta*	leaves and berries	China	USDA
Colletotrichum fructicola	*Coffea* sp.	/	Angola	USDA
Colletotrichum gigasporum	*Coffea arabica*	unripe fruit	Mexico	USDA
Colletotrichum gigasporum	*Coffea canephora var. robusta*	leaves and berries	China	USDA
Colletotrichum gigasporum	*Coffea* sp.	/	Viet Nam	USDA
Colletotrichum gloeosporioides	*Coffea arabica*	ripe fruit	Brazil, China, Cuba, El Salvador, Guatemala, Indonesia, Jamaica, Kenya, Madagascar,	USDA, [27]

			Malawi, Mexico, Papua New Guinea, Peru, Puerto Rico, Sri Lanka, Venezuela, Viet Nam, Virgin Islands	
Colletotrichum gloeosporioides	*Coffea canephora var. robusta*	plant	Sri Lanka	USDA
Colletotrichum gloeosporioides	*Coffea liberica*	plant	Indonesia	USDA
Colletotrichum gloeosporioides	*Coffea* sp.	berries	Brazil, South Africa, Sri Lanka	USDA
Colletotrichum gloeosporioides	*Cape Australia Coffee Estate*	berries	Papua New Guinea	[28]
Colletotrichum kahawae	*Coffea arabica*	berrirs	Angola, Burundi, Cameroon, Ethiopia, Kenya, Malawi, Rwanda, Tanzania, Thailand, Uganda, Zambia, Zimbabwe	USDA
Colletotrichum kahawae	*Coffea* sp.	berrirs	Kenya, Malawi	USDA
Colletotrichum kahawae subsp. *kahawae* – (*Colletotrichum kahawae*)	*Coffea arabica*	berrirs	Angola, Cameroon, Kenya, Malawi	USDA
Colletotrichum kahawae subsp. *kahawae* – (*Colletotrichum kahawae*)	*Coffea* sp.	plant	Africa, Kenya	USDA
Colletotrichum kahawae subsp. *ciggaro*	*Coffea* sp.	fruit	Puerto Rico	[27]
Colletotrichum karstii – (*Colletotrichum karsti*)	*Coffea arabica*	leaf	Mexico	USDA
Colletotrichum karstii – (*Colletotrichum karsti*)	*Coffea canephora var. robusta*	leaves and berries	China	USDA
Colletotrichum karstii – (*Colletotrichum karsti*)	*Coffea* sp.	leaves	Viet Nam	USDA
Colletotrichum ledongense	*Coffea canephora var. robusta*	leaves and berries	China	USDA
Colletotrichum magnisporum	*Coffea* sp.	plant	Hawaii	USDA
Colletotrichum plurivorum	*Coffea* sp.	leaf	Viet Nam	USDA
Colletotrichum queenslandicum	*Coffea* sp.	/	Fiji	USDA
Colletotrichum siamense	*Coffea arabica*	leaves and berries	Australia, China, Mexico, Puerto Rico,	USDA,

			Thailand	[27]
Colletotrichum siamense	*Coffea canephora*	leaves	Australia	USDA
Colletotrichum siamense	*Coffea canephora var. robusta*	leaves and berries	China	USDA
Colletotrichum siamense	*Coffea* sp.	/	Brazil, China, Colombia, Kenya, Malawi	USDA
Colletotrichum siamense	*Coffea arabica*	coffee berries	Thailand Chiang Mai	[26]
Colletotrichum sp.	*Coffea arabica*	leaves	Australia, Brazil, China, India, Mexico, Papua New Guinea, Venezuela	USDA
Colletotrichum sp.	*Coffea canephora*	plant	Brazil	USDA
Colletotrichum sp.	*Coffea robusta*	berries	India	USDA
Colletotrichum sp.	*Coffea* sp.	plant	Malay Peninsula	USDA
Colletotrichum theobromicola	*Coffea arabica*	coffee berries	Australia, Mexico, Puerto Rico	USDA, [27]
Colletotrichum tropicale	*Coffea arabica*	green coffee fruits	Puerto Rico	[27]
Colletotrichum tropicale	*Coffea canephora var. robusta*	leaves and berries	China	USDA
Colletotrichum vietnamense	*Coffea* sp.	leaf	Viet Nam	USDA
Colletotrichum walleri	*Coffea arabica*	leaf tissue	Viet Nam	USDA
Colletotrichum walleri	*Coffea* sp.	leaf tissue	Viet Nam	USDA
Colletotrichum falcatum	*Coffea arabica*	coffee berries	Thailand Chiang Mai	[26]
Colletotrichum gloeosporioides	*Coffea arabica*	coffee berries	Thailand Chiang Mai	[26]
Colletotrichum asianum	*Coffea arabica*	coffee berries	Thailand Chiang Mai	[26]
Colletotrichum crassipes	*Coffea arabica*	leaves	/	[25]
Colletotrichum musae	*Coffea arabica*	leaves	Mexico	[22]
Colletotrichum aff brassicicola	*Coffea arabica*	leaves	Mexico	[22]
Glomerella cingulata – (*Colletotrichum gloeosporioides*)	*Coffea arabica*	plant	Congo, Cuba, Fiji, Florida, India, Japan, Kenya, Madagascar, New Caledonia, Papua New Guinea, Range of host, Samoa, Tanzania, Uganda, Venezuela, Zimbabwe	USDA
Glomerella cingulata – (*Colletotrichum gloeosporioides*)	*Coffea bukobensis*	all-aged plants	Tanzania	USDA
Glomerella cingulata – (*Colletotrichum gloeosporioides*)	*Coffea canephora*	plant	Fiji, Samoa	USDA
Glomerella cingulata –	*Coffea canephora var. robusta*	all-aged plants	Indonesia, Tanzania, Vanuatu	USDA

	(Colletotrichum gloeosporioides)				
	Glomerella cingulata – *(Colletotrichum gloeosporioides)*	*Coffea excelsa*	all-aged plants	Tanzania	USDA
	Glomerella cingulata – *(Colletotrichum gloeosporioides)*	*Coffea liberica*	plant	Brunei Darussalam	USDA
	Glomerella cingulata – *(Colletotrichum gloeosporioides)*	*Coffea robusta*	plant	Brunei Darussalam, New Caledonia, Tanzania	USDA
	Glomerella cingulata – *(Colletotrichum gloeosporioides)*	*Coffea* sp.	plant	Fiji, Kenya, Malawi, Mauritius, Samoa, South Africa	USDA
	Glomerella coffeicola	*Coffea arabica*	leaves, shoots	Brazil	USDA
	Glomerella phomoides – *(Colletotrichum gloeosporioides)*	*Coffea arabica*	plant	Fiji	USDA
	Glomerella phomoides – *(Colletotrichum gloeosporioides)*	*Coffea canephora*	plant	Fiji	USDA
	Vermicularia sp.	*Coffea* sp.	plant	Fiji	USDA
Gnomoniaceae (2)	*Ophiognomonia* sp.	*Coffea arabica*	leaves	Brazil	[20]
	Laestadia coffeicola	*Coffea arabica*	leaves	Costa Rico	USDA
Graphidaceae (1)	*Graphis* sp.	*Coffea arabica*	plant	Venezuela	USDA
Graphostromataceae (1)	*Biscogniauxia* sp.	*Coffea arabica*	/	Hawaii	USDA
Helicobasidiaceae (2)	*Helicobasidium compactum*	*Coffea arabica*	plant	East Indies, El Salvador, Guatemala	USDA
	Helicobasidium longisporum	*Coffea* sp.	/	United States	USDA
Hermatomycetaceae (1)	*Hermatomyces tucumanensis*	*Coffea liberica*	/	Sierra Leone	USDA
Hymenochaetaceae (11)	*Hymenochaete coffeana*	*Coffea robusta*	/	Central African Republic	USDA
	Hymenochaete noxia	*Coffea arabica*	/	Kenya	USDA
	Phellinus lamaensis	*Coffea robusta*	/	New Caledonia	USDA
	Phellinus lamaensis	*Coffea* sp.	plant	Fiji, Papua New Guinea, Samoa	USDA

	Phellinus noxius – (*Phellinidium noxium*)	*Coffea arabica*	fruit	Papua New Guinea, Taiwan	USDA
	Phellinus noxius – (*Phellinidium noxium*)	*Coffea canephora*	/	Papua New Guinea	USDA
	Phellinus noxius – (*Phellinidium noxium*)	*Coffea* sp.	/	Fiji	USDA
	Polystictus occidentalis	*Coffea arabica*	/	Kenya, Tanzania	USDA
	Polystictus phaeus	*Coffea arabica*	plant	Taiwan	USDA
	Pyrrhoderma noxium – (*Phellinidium noxium*)	*Coffea arabica*	/	Sri Lanka	USDA
Hypocreaceae (26)	*Acrostalagmus aphidum* – (*Lecanicillium longisporum*)	*Coffea arabica*	/	Puerto Rico, Virgin Islands	USDA
	Gliocladium catenulatum – (*Clonostachys rosea f. catenulata*)	*Coffea arabica*	/	Cuba	USDA
	Hypocrea lixii	*Coffea* sp.	Coffee residue compost	Hiroshima prefecture	[13]
	Hypocrea virens	*Coffea* sp.	Coffee residue compost	Hiroshima prefecture	[13]
	Hypomyces haematococcus – (*Neocosmospora haematococca*)	*Coffea abeokutae*	/	Cote d'Ivoire	USDA
	Hypomyces haematococcus – (*Neocosmospora haematococca*)	*Coffea canephora*	/	Cote d'Ivoire	USDA
	Hypomyces haematococcus – (*Neocosmospora haematococca*)	*Coffea liberica*	/	Cote d'Ivoire	USDA
	Hypomyces haematococcus var. breviconus – (*Neocosmospora brevicona*)	*Coffea abeokutae*	/	Cote d'Ivoire	USDA
	Hypomyces haematococcus var.	*Coffea canephora*	/	Cote d'Ivoire	USDA

	breviconus – (*Neocosmospora brevicona*)				
	Hypomyces haematococcus var. breviconus – (*Neocosmospora brevicona*)	*Coffea liberica*	/	Cote d'Ivoire	USDA
	Trichoderma sp.	*Coffea arabica*	leaves	Brazil	[20]
	Trichoderma flagellatum	*Coffea arabica*	roots	Ethiopia	USDA
	Trichoderma atroviride	*Coffea* sp.	Leaf	Kenya	[29]
	Trichoderma botryosum sp. *nov.*	*Coffea canephora*	Stem	Cameroon	[29]
	Trichoderma botryosum sp. *nov.*	*Coffea arabica*	Stem, berries, leaf	Ethiopia, Cameroon	[29]
	Trichoderma breve	*Coffea canephora*	Stem	Cameroon	[29]
	Trichoderma breve	*Coffea arabica*	Berry	Ethiopia	[29]
	Trichoderma caeruloviride sp. *nov.*	*Coffea arabica*	Stem	Ethiopia	[29]
	Trichoderma guizhouense	*Coffea* sp.	Stem	Kenya	[29]
	Trichoderma hamatum	*Coffea arabica*	Stem, berries	Ethiopia	[29]
	Trichoderma koningiopsis	*Coffea canephora*	Leaf, Stem	Cameroon	[29]
	Trichoderma lentissimum sp. *nov*	*Coffea cf. arabica*	Stem	Kenya	[29]
	Trichoderma parareesei	*Coffea arabica*	Stem	Ethiopia	[29]
	Trichoderma pseudopyramidale sp. *nov.*	*Coffea arabica*	Leaf, Stem	Ethiopia	[29]
	Trichoderma spirale	*Coffea canephora*	Stem	Cameroon	[29]
	Trichoderma theobromicola	*Coffea canephora*	Stem	Cameroon	[29]
	Trichoderma virens	*Coffea brevipes*	Stem	Cameroon	[29]
Hyponectriaceae (1)	*Physalospora* sp.	*Coffea robusta*	plant	Malaysia	USDA
Hypoxylaceae (4)	*Hypoxylon deustum* – (*Kretzschmaria deusta*)	*Coffea arabica*	/	Fiji	USDA
	Hypoxylon deustum – (*Kretzschmaria deusta*)	*Coffea* sp.	/	Fiji	USDA
	Hypoxylon stygium – (*Annulohypoxylon stygium*)	*Coffea arabica*	/	Puerto Rico, Virgin Islands	USDA

	Nodulisporium gregarium	*Coffea arabica*	Mature and healthy leaves	Brazil	[21]
Incertae sedis (93)	*Acremonium* sp.	*Coffea arabica*	green coffee seeds, root	Puerto Rico, USA, Hawaii, Kona, Kona Experimental Station	[5]
	Acremonium sp.	*Coffea canephora*	/	Papua New Guinea	USDA
	Acremonium alternatum	*Coffea arabica*	Epicarp	Colombia, Caldas, Chinchina	[5]
	Aecidium nobile	*Coffea arabica*	/	India	USDA
	Aecidium travancoricum	*Coffea travancorensis*	leaves	India	USDA
	Annellophora sp.	*Coffea liberica*	plant	Malaysia	USDA
	Arxiella terrestris	*Coffea arabica*	/	Australia	USDA
	Bahusutrabeeja dwaya	*Coffea arabica*	/	India	USDA
	Botryodiplodia sp.	*Coffea* sp.	/	Papua New Guinea	USDA
	Botryodiplodia theobromae – (*Lasiodiplodia theobromae*)	*Coffea abeokutae*	/	Cote d'Ivoire	USDA
	Botryodiplodia theobromae – (*Lasiodiplodia theobromae*)	*Coffea arabica*	plant	Cuba, Fiji, Ghana, Tanzania	USDA
	Botryodiplodia theobromae – (*Lasiodiplodia theobromae*)	*Coffea canephora*	plant	Cote d'Ivoire, Fiji, Papua New Guinea	USDA
	Botryodiplodia theobromae – (*Lasiodiplodia theobromae*)	*Coffea liberica*	plant	Brunei Darussalam, Cote d'Ivoire	USDA
	Botryodiplodia theobromae – (*Lasiodiplodia theobromae*)	*Coffea robusta*	plant	Brunei Darussalam	USDA
	Botryodiplodia theobromae – (*Lasiodiplodia theobromae*)	*Coffea* sp.	plant	Ghana, Malay Peninsula	USDA
	Camposporium antennatum	*Coffea arabica*	/	Australia	USDA
	Candida albicans	Café Najjar	coffee powder	Abuja	[12]
	Cephalosporium deformans	*Coffea arabica*	plant	El Salvador	USDA
	Cephalosporium lecanii – (*Lecanicillium lecanii*)	*Coffea arabica*	/	Puerto Rico, Venezuela, Virgin Islands	USDA

Cephalosporium omnivorum	*Coffea arabica*	plant	El Salvador	USDA
Cephalosporium zonatum – (cremonium zonatum)	*Coffea arabica*	plant	Costa Rica, Puerto Rico, Virgin Islands	USDA
Cephalosporium sp.	*Coffea arabica*	/	Puerto Rico	USDA
Chaetostroma sp.	*Coffea arabica*	plant	Mexico	USDA
Cladosporiella deightonii	*Coffea arabica*	leaves	Cuba	USDA
Clitocybe tabescens – (Desarmillaria tabescens)	*Coffea canephora*	roots	Madagascar	USDA
Clitocybe tabescens – (Desarmillaria tabescens)	*Coffea canephora var. robusta*	roots	Madagascar	USDA
Clypeolum megalosporium	*Coffea arabica*	/	Costa Rica	USDA
Diacanthodes novoguineensis	*Coffea arabica*	/	Papua New Guinea	USDA
Diachea leucopodia	*Coffea arabica*	/	India	USDA
Dinemasporium coffeanum	*Coffea arabica*	/	Brazil	USDA
Diplocladiella scalaroides	*Coffea arabica*	/	China, Japan, Taiwan	USDA
Ectophoma pomi	*Coffea arabica*	leaf	India	USDA
Eriomycopsis sp.	*Coffea arabica*	plant	Sudan	USDA
Fumago vagans	*Coffea arabica*	/	China	USDA
Hemileia coffeicola	*Coffea arabica*	leaves	Cameroon, Gabon	USDA
Hemileia coffeicola	*Coffea laurina*	/	Cameroon	USDA
Hemileia coffeicola	*Coffea* sp.	leaves	Cameroon, India	USDA
Hemileia vastatrix	*Coffea arabica*	leaves	Africa, Angola, Asia, Brazil, Cambodia, Central America, China, Colombia, Congo, Cook Islands, Costa Rica, East Africa, Ethiopia, Fiji, India, Indonesia, Japan, Kenya, Madagascar, Malawi, Malay Peninsula, Mexico, New Caledonia, Nigeria, Pacific Islands, Pakistan, Panama, Papua New Guinea, Philippines, Puerto Rico Rwanda, Samoa, South Africa, South America, Southern Africa, Sri Lanka, Sudan, Taiwan, Tanzania, Thailand, Uganda Venezuela, Viet Nam, Zimbabwe	USDA
Hemileia vastatrix	*Coffea arabica var. mokka*	leaves	South Africa	USDA
Hemileia vastatrix	*Coffea arabica var. myrtifolia*	leaves	Congo	USDA

Hemileia vastatrix	*Coffea bengalensis*	/	India	USDA
Hemileia vastatrix	*Coffea canephora*	leaves	Asia, Brazil, Cambodia, Cameroon, India, Indonesia, Philippines, South America, Sri Lanka, Tanzania, Uganda, West Africa	USDA
Hemileia vastatrix	*Coffea canephora var. robusta*	leaves	India, Indonesia, Madagascar, Philippines, Sudan, Tanzania, Uganda	USDA
Hemileia vastatrix	*Coffea congensis*	leaves	India, Madagascar	USDA
Hemileia vastatrix	*Coffea congensis var. chalotii*	leaves	Uganda	USDA
Hemileia vastatrix	*Coffea eugenioides*	leaves	India, Kenya	USDA
Hemileia vastatrix	*Coffea excelsa*	leaves	China, Ethiopia, India, Philippines, Tanzania, Uganda	USDA
Hemileia vastatrix	*Coffea laurina*	leaves	Indonesia	USDA
Hemileia vastatrix	*Coffea liberica*	leaves	Africa, Asia, Brunei Darussalam, Cambodia, Central America, China, Fiji, India, Indonesia, Japan, Madagascar, Malay Peninsula, Malaysia, Samoa, Tanzania, Uganda, West Africa	USDA
Hemileia vastatrix	*Coffea quillon*	leaves	Indonesia	USDA
Hemileia vastatrix	*Coffea robusta*	leaves	Africa, China, India, Japan, Malay Peninsula, Malaysia, Philippines, Sudan, Taiwan, Tanzania, Thailand	USDA
Hemileia vastatrix	*Coffea* sp.	leaves	Africa, Asia, Australia, Brazil, China, Central America, China, Cook Islands, Costa Rica, Ethiopia, Fiji, Guinea, Malawi, Malaysia, Mauritius, New Guinea, Philippines, Samoa, South Africa, South America, Thailand, Vanuatu	USDA
Hemileia vastatrix	*Coffea stenophylla*	leaves	Sri Lanka	USDA
Hemileia vastatrix	*Coffea arabica–canephora*	leaves	Brazil, Colombia, Indonesia	USDA
Hemileia vastatrix	*Coffea arabica–liberica*	leaves	Uganda	USDA
Hyphopichia burtonii A	*Coffea* sp.	coffee waste	Ethiopia (Sidama and Gedio)	USDA
Imimyces densus	*Coffea excelsa*	/	Sierra Leone	USDA
Leptothyrium discoideum	*Coffea arabica*	/	Venezuela	USDA
Myrothecium advena – (Paramyrothecium roridum)	*Coffea* sp.	plant	Malay Peninsula	USDA
Myrothecium roridum – (Paramyrothecium	*Coffea arabica*	stems, leaves	Colombia, Costa Rica, Guatemala, Puerto Rico, Virgin Islands	USDA

roridum)				
Myrothecium roridum – *(Paramyrothecium roridum)*	*Coffea canephora*	leaves	Brazil	USDA
Myrothecium roridum – *(Paramyrothecium roridum)*	*Coffea canephora var. robusta*	stems, leaves	Colombia	USDA
Myrothecium roridum – *(Paramyrothecium roridum)*	*Coffea liberica*	stems, leaves	Colombia	USDA
Myrothecium sp.	*Coffea arabica*	plant	Florida	USDA
Nigrospora oryzae	*Coffea arabica*	plant	Japan	USDA
Nigrospora sp.	*Coffea arabica*	plant	Brazil	USDA
Nigrospora sp.	*Coffea* sp.	plant	Malay Peninsula	USDA
Nigrospora sphaerica	*Coffea robusta*	plant	Brunei Darussalam	USDA
Omphalia flavida – *(Mycena citricolor)*	*Coffea arabica*	plant	El Salvador, Jamaica, Mexico, Trinidad and Tobago	USDA
Periconia sp.	*Coffea arabica*	leaves	/	[25]
Periconia byssoides	*Coffea arabica*	plant	Venezuela	USDA
Plenotrichopsis coffeae	*Coffea arabica*	plant	Brazil	USDA
Podosporium sp.	*Coffea* sp.	plant	Ghana	USDA
Podoxyphium sp.	*Coffea arabica*	plant	Myanmar,	USDA
Pseudomorfea coffeae	*Coffea arabica*	/	India	USDA
Sclerotium coffeicola	*Coffea arabica*	leaves, fruits	Brazil, Costa Rica, Guyana	USDA
Sclerotium coffeicola	*Coffea dewevrei*	leaves, fruits	Guyana	USDA
Sclerotium coffeicola	*Coffea liberica*	leaves, fruits	Guyana, Trinidad and Tobago	USDA
Sclerotium coffeicola	*Coffea robusta*	/	Trinidad and Tobago	USDA
Sclerotium coffeicola	*Coffea* sp.	plant	Costa Rica, Suriname	USDA
Sclerotium rolfsii – *(Athelia rolfsii)*	*Coffea arabica*	plant	Kenya, Thailand	USDA
Sclerotium rolfsii – *(Athelia rolfsii)*	*Coffea* sp.	plant	Malay Peninsula	USDA
Sclerotium sp.	*Coffea* sp.	plant	Philippines	USDA
Sporidesmium coffeicola	*Coffea* sp.	/	New Guinea	USDA
Stachylidium bicolor	*Coffea arabica*	/	Papua New Guinea	USDA
Stilbella flavidum –	*Coffea arabica*	leaves	Colombia, Dominican Republic, Mexico,	USDA

	(*Mycena citricolor*)			Venezuela	
	Stilbella sp.	*Coffea arabica*	/	Papua New Guinea	USDA
	Staninwardia sp.	*Coffea arabica*	leaves	/	[25]
	Trichothecium roseum	*Coffea arabica*	plant	Florida	USDA
	Triposporium sp.	*Coffea canephora*	/	Papua New Guinea	USDA
	Khuskia oryzae	*Coffea arabica*	Mature and healthy leaves	Brazil	[21]
	Sarocladium bacillisporum	*Coffea arabica*	Mature and healthy leaves	Brazil	[21]
	Ascospora coffeae	*Coffea robusta*	/	Java	USDA
Irpicaceae (4)	*Irpex flavus* – (*Flavodon flavus*)	*Coffea robusta*	plant	Malaysia	USDA
	Leptoporus lignosus	*Coffea abeokutae*	/	Cote d'Ivoire	USDA
	Leptoporus lignosus	*Coffea canephora*	/	Cote d'Ivoire	USDA
	Leptoporus lignosus	*Coffea liberica*	/	Cote d'Ivoire	USDA
Leptosphaeriaceae (21)	*Leptosphaeria africana*	*Coffea robusta*	/	South Africa	USDA
	Leptosphaeria canephorae	*Coffea canephora*	/	Congo, Democratic Republic	USDA
	Leptosphaeria centrafricana	*Coffea excelsa*	/	Central African Republic	USDA
	Leptosphaeria coffaeicida	*Coffea* sp.	/	Costa Rica	USDA
	Leptosphaeria coffeicola	*Coffea arabica*	plant	Malawi, Tanzania	USDA
	Leptosphaeria coffeicola	*Coffea robusta*	plant	Malaysia	USDA
	Leptosphaeria coffeigena	*Coffea arabica*	plant	Cuba, Mexico, Venezuela	USDA
	Leptosphaeria coffeigena var. longirostrata	*Coffea canephora*	/	Cote d'Ivoire	USDA
	Leptosphaeria cylindrospora	*Coffea robusta*	/	Central African Republic	USDA
	Leptosphaeria excelsa	*Coffea excelsa*	/	Central African Republic	USDA
	Leptosphaeria gigaspora – (*Wettsteinina niesslii*)	*Coffea robusta*	/	Central African Republic	USDA
	Leptosphaeria lobayensis	*Coffea excelsa*	/	Central African Republic	USDA
	Leptosphaeria longispora	*Coffea excelsa*	/	Central African Republic	USDA
	Leptosphaeria macrorostra	*Coffea robusta*	/	Central African Republic	USDA
	Leptosphaeria oubanguiensis	*Coffea robusta*	/	Central African Republic	USDA
	Leptosphaeria pusilla	*Coffea* sp.	/	Costa Rica	USDA
	Leptosphaeria sp.	*Coffea arabica*	Plant, leaves	Tanzania	USDA

	Leptosphaeria sp.	*Coffea robusta*	plant	Brunei Darussalam	USDA
	Leptosphaeria tetraspora	*Coffea robusta*	/	Central African Republic	USDA
	Leptosphaeria tonduzi – (*Leptosphaeria tonduzii*)	*Coffea* sp.	/	Costa Rica	USDA
	Leptosphaeria sp.	*Coffea arabica*	leaves	/	[25]
Lichtheimiaceae (1)	*Lichtheimia ramosa*	*Coffea arabica*	beans	/	[2]
Marasmiaceae (4)	*Marasmius equicrinis* – (*Marasmius crinis-equi*)	*Coffea abeokutae*	/	Cote d'Ivoire	USDA
	Marasmius equicrinis – (*Marasmius crinis-equi*)	*Coffea arabica*	plant	Trinidad and Tobago	USDA
	Marasmius equicrinis – (*Marasmius crinis-equi*)	*Coffea liberica*	/	Cote d'Ivoire	USDA
	Marasmius sp.	*Coffea* sp.	Plant	Malay Peninsula	USDA
Massarinaceae (8)	*Helminthosporium canephorae*	*Coffea canephora*	/	Congo, Democratic Republic	USDA
	Helminthosporium coffeae	*Coffea* sp.	plant	Ghana	USDA
	Helminthosporium coffeae	*Coffea arabica*	plant	Ghana	USDA
	Helminthosporium glabroides	*Coffea arabica*	/	Ethiopia	USDA
	Helminthosporium sp.	*Coffea arabica*	plant	Nicaragua	USDA
	Helminthosporium sp.	*Coffea* sp.	/	Papua New Guinea	USDA
	Helminthosporium ubangiense	*Coffea* sp.	leaves	Tropical Africa	USDA
	Oraniella coffeicola – (*Massarina coffeicola*)	*Coffea* sp.	/	Hawaii	USDA
Meliolaceae (11)	*Asteridiella naucleae var. libericae*	*Coffea liberica*	/	Sierra Leone	USDA
	Irenina coffeae	*Coffea abeokutae*	/	Cote d'Ivoire	USDA
	Irenina glabra	*Coffea liberica*	Plant	Sierra Leone	USDA
	Irenina glabra	*Coffea* sp.	/	Uganda	USDA
	Meliola coffeae	*Coffea arabica*	/	Ghana	USDA
	Meliola coffeae	*Coffea robusta*	leaves	Ghana, Uganda	USDA
	Meliola psychotriae	*Coffea arabica*	/	Ghana	USDA
	Meliola psychotriae var. coffeae	*Coffea stenophylla*	/	Sierra Leone	USDA
	Meliola rogeri	*Coffea arabica*	/	Ethiopia	USDA
	Meliola sp.	*Coffea arabica*	plant	Ghana	USDA

	Meliola sp.	*Coffea liberica*	plant	Ghana	USDA
Meripilaceae (3)	*Rigidoporus lignosus* – (*Rigidoporus microporus*)	*Coffea liberica*	plant	Brunei Darussalam	USDA
	Rigidoporus lignosus – (*Rigidoporus microporus*)	*Coffea robusta*	plant	Brunei Darussalam	USDA
	Rigidoporus microporus	*Coffea* sp.	/	Papua New Guinea	USDA
Meruliaceae (1)	*Phlebia subserialis*	*Coffea arabica*	Coffee soil	Colombian andisols	[17]
Metacapnodiaceae (1)	*Antennaria setosa*	*Coffea arabica*	/	Belgium	USDA
Micropeltidaceae (12)	*Dictyothyriella mucosa* – (*Micropeltis mucosa*)	*Coffea excelsa*	/	Dominican Republic	USDA
	Dictyothyriella mucosa – (*Micropeltis mucosa*)	*Coffea* sp.	plant	Philippines	USDA
	Micropeltella mulleri	*Coffea arabica*	leaves	Brazil	USDA
	Micropeltidium tonduzii	*Coffea arabica*	/	Costa Rica	USDA
	Micropeltis applanata	*Coffea arabica*	leaves	El Salvador	USDA
	Micropeltis mucosa	*Coffea arabica*	/	Philippines	USDA
	Micropeltis mucosa	*Coffea excelsa*	/	Philippines	USDA
	Micropeltis mucosa	*Coffea* sp.	plant	Philippines	USDA
	Parapeltella coffeicola	*Coffea arabica*	/	Guatemala	USDA
	Scolecopeltella longispora	*Coffea arabica*	/	Puerto Rico	USDA
	Scolecopeltis aeruginea	*Coffea liberica*	/	Indonesia	USDA
	Scolecopeltis longispora	*Coffea arabica*	/	Puerto Rico, Virgin Islands	USDA
Microthyriaceae (1)	*Scolecopeltidium coffeae*	*Coffea arabica*	plant	Brazil	USDA
Microascaceae (1)	*Microascus* sp.	*Coffea arabica*	leaves	/	[25]
Microsphaeropsidaceae (1)	*Microsphaeropsis* sp.	*Coffea arabica*	leaves	/	[25]
Mollisiaceae (1)	*Phialocephala mexicana*	*Coffea arabica*	leaves	Cote d'Ivoire	USDA
Mycenaceae (7)	*Mycena citricolor*	*Coffea abeokutae*	leaves, twigs	Puerto Rico	USDA
	Mycena citricolor	*Coffea arabica*	leaves, twigs	Brazil, Colombia, Cuba, Haiti, Mexico, Panama, Puerto Rico, Venezuela, West Indies	USDA
	Mycena citricolor	*Coffea canephora*	leaves, twigs	Puerto Rico	USDA
	Mycena citricolor	*Coffea canephora var. laurentii*	leaves, twigs	Puerto Rico	USDA
	Mycena citricolor	*Coffea canephora var. robusta*	leaves, twigs	Trinidad, Tobago	USDA
	Mycena citricolor	*Coffea liberica*	leaves, twigs	Puerto Rico	USDA
	Mycena citricolor	*Coffea* sp.	/	Puerto Rico	USDA
Mycosphaerellaceae	*Cercospora* sp.	*Coffea arabica*	leaves	Brazil	[20]

(33)

Cercospora coffeae – (*Cercospora coffeicola*)	*Coffea stenophylla*	/	Africa	USDA
Cercospora coffeae-olivaceae	*Coffea olivacea*	/	India	USDA
Cercospora coffeicola	*Coffea abeokutae*	/	Cote d'Ivoire	USDA
Cercospora coffeicola	*Coffea arabica*	leaves, berries	Australia, Brazil, Cambodia, China, Colombia, Congo, Democratic Republic, Cook Islands, Costa Rica, Cote d'Ivoire, Cuba, Dominican Republic, El Salvador, Fiji, Florida, Guatemala, Guinea, Haiti, Hawaii, Honduras, India, Indonesia, Jamaica, Japan, Kenya, Madagascar, Malawi, Mexico, Myanmar, New Caledonia, Nicaragua, Panama, Papua New Guinea, Peru, Philippines, Puerto Rico, Samoa, Sierra Leone, Sudan, Taiwan, Tanzania, Thailand, Trinidad and Tobago, Uganda, Venezuela, Virgin Islands, Zambia, Zimbabwe	USDA, [30]
Cercospora coffeicola	*Coffea bukobensis*	leaves	Philippines	USDA
Cercospora coffeicola	*Coffea canephora*	plant	Cambodia, Fiji, Papua New Guinea, Samoa, Sierra Leone	USDA
Cercospora coffeicola	*Coffea canephora var. robusta*	leaves	Guinea, Sudan	USDA
Cercospora coffeicola	*Coffea excelsa*	leaves, plant	China, Philippines, Sudan	USDA
Cercospora coffeicola	*Coffea liberica*	plant	Brunei Darussalam, China, Cote d'Ivoire, Cuba, Fiji, Sierra Leone	USDA
Cercospora coffeicola	*Coffea* sp.	leaves	Fiji, Ghana, Haiti, Jamaica, Malawi, Mauritius, Nicaragua, Nigeria, Papua New Guinea, Samoa, South Africa, Southern Africa, Thailand	USDA
Cercospora coffeicola	*Coffea robusta*	plant	Brunei Darussalam, China, Hawaii, Malawi, New Caledonia, Sierra Leone, Sudan, Taiwan, Trinidad and Tobago	USDA
Cercospora coffeicola	*Coffea stenophylla*	leaves	Sierra Leone, Tanzania	USDA
Cercospora herrerana – (*Cercospora coffeicola*)	*Coffea arabica*	/	Mexico	USDA
Cercospora sp.	*Coffea arabica*	leaves	Brazil, Japan	USDA
Isariopsis acanthacearum	*Coffea liberica*	plant	Malaysia	USDA
Mycosphaerella coffeae	*Coffea arabica*	leaves	Brazil, Cuba, Samoa, Taiwan	USDA
Mycosphaerella coffeae	*Coffea arabica var. myrtifolia*	leaves	Congo, Democratic Republic, Congo Republic,	USDA

				Samoa	
	Mycosphaerella coffeae	*Coffea robusta*	plant	Taiwan	USDA
	Mycosphaerella coffeae	*Coffea* sp.	/	Samoa	USDA
	Mycosphaerella coffeicola – (*Cercospora coffeicola*)	*Coffea arabica*	leaves	Africa, Cambodia, El Salvador, India, Jamaica, Mexico, Puerto Rico, Virgin Islands	USDA
	Mycosphaerella coffeicola – (*Cercospora coffeicola*)	*Coffea* sp.	plant	Mauritius, South Africa	USDA
	Mycosphaerella sp.	*Coffea arabica*	leaves	Bolivia, Brazil	USDA, [20]
	Pseudocercospora coffeigena	*Coffea arabica*	plant	China	USDA
	Septoria berkeleyi	*Coffea arabica*	plant	Brazil	USDA
	Septoria berkeleyi	*Coffea arabica*	plant	South America	USDA
	Septoria coffeicola	*Coffea liberica*	leaves	Cameroon	USDA
	Septoria sp.	*Coffea arabica*	plant	Brazil	USDA
	Sphaerella coffeicola – (*Cercospora coffeicola*)	*Coffea* sp.	/	Venezuela	USDA
	Sphaerella sp.	*Coffea arabica*	plant	Mexico	USDA
	Sphaerulina coffeicola	*Coffea arabica*	/	Dominican Republic, Eritrea, Haiti	USDA
	Septoria coffeae	*Coffea arabica*	/	Uganda	USDA
	Zasmidium coffeae	*Coffea bengalensis*	/	India	USDA
Teratosphaeriaceae (1)	*Stenella coffeae*	*Coffea bengalensis*	leaves	India	USDA
Mucoraceae (1)	*Mucor* sp.	*Coffea arabica/robusta*	beans	Philippines	[31]
Nectriaceae (77)	*Calonectria pyrochroa*	*Coffea* sp.	/	India	USDA)
	Calonectria sp.	*Coffea arabica*	/	Uganda	USDA
	Cylindrocarpon sp.	*Coffea arabica*	/	Venezuela	USDA
	Cylindrocarpon tenue – (*Gliocladiopsis tenuis*)	*Coffea arabica*	/	India	USDA
	Cylindrocladiella parva	*Coffea* sp.	/	India	USDA)
	Cylindrocladium scoparium – (*Calonectria cylindrospora*)	*Coffea arabica*	/	Taiwan	USDA
	Fusarium acuminatum	*Coffea* sp.	Coffee residue compost	Hiroshima prefecture	[13]
	Fusarium camptoceras	*Coffea* sp.	Root of wilting *Coffea*	Angola	USDA
	Fusarium coffeicola	*Coffea arabica*	/	Brazil, El Salvador	USDA
	Fusarium concolor	*Coffea arabica*	plant	Brazil	USDA

Fusarium decemcellulare – (*Albonectria rigidiuscula*)	*Coffea canephora*	plant	Brazil	USDA
Fusarium equiseti	*Coffea arabica*	seed	Brazil	USDA
Fusarium equiseti	*Coffea arabica*	beans	Lavras /Minas Gerais.	[14]
Fusarium lateritium	*Coffea arabica*	berries, twig, bark	Brazil, El Salvador, Ethiopia, Malawi, New Caledonia, New Guinea, Papua New Guinea, Tanzania, Zimbabwe	USDA
Fusarium lateritium	*Coffea canephora*	plant	Brazil, Guinea	USDA
Fusarium lateritium	*Coffea* sp.	plant	Malawi	USDA
Fusarium lateritium subsp. *lateritium*	*Coffea arabica*	plant	Brazil	USDA
Fusarium lateritium var. longum – (*Fusarium stilboides*)	*Coffea arabica*	plant	Costa Rica, Panama, Tanzania	USDA
Fusarium moniliforme	*Coffea arabica*	plant	Brazil	USDA
Fusarium oxysporum	*Coffea arabica*	plant	Brazil, Costa Rica, El Salvador, Florida, Guatemala, Kenya, Mexico, Puerto Rico, Virgin Islands	USDA
Fusarium oxysporum	*Coffea canephora*	plant	Puerto Rico, Virgin Islands	USDA
Fusarium oxysporum	*Coffea canephora var. robusta*	plant	Congo	USDA
Fusarium oxysporum	*Coffea excelsa*	/	Puerto Rico, Virgin Islands	USDA
Fusarium oxysporum	*Coffea* sp.	seed	Puerto Rico	USDA
Fusarium oxysporum	Catuai variety	corticous roots tissues	Piumhi, Brazil	[6]
Fusarium oxysporum f. conglutinans	*Coffea stenophylla*	/	Puerto Rico, Virgin Islands	USDA
Fusarium oxysporum f. sp. *coffeae*	*Coffea arabica*	plant	Brazil	USDA
Fusarium pallidoroseum – (*Fusarium incarnatum*)	*Coffea arabica*	plant	Brazil	USDA
Fusarium roseum	*Coffea arabica:*	/	Brazil	USDA
Fusarium semitectum	*Coffea arabica*	beans	Lavras, Minas Gerais.	[14]
Fusarium semitectum – (*Fusarium incarnatum*)	*Coffea arabica*	plant	Venezuela	USDA
Fusarium semitectum – (*Fusarium incarnatum*)	*Coffea liberica*	plant	Malaysia	USDA
Fusarium solani	Catuai variety	corticous roots tissues	Piumhi, Brazil	[6]
Fusarium solani –	*Coffea arabica*	stem	Brazil, Haiti, Malaysia, Papua New Guinea,	USDA

(*Neocosmospora solani*)			Viet Nam	
Fusarium solani – (*Neocosmospora solani*)	*Coffea canephora*	plant	Brazil	USDA
Fusarium solani – (*Neocosmospora solani*)	*Coffea* sp.	seed	India, Papua New Guinea	USDA
Fusarium solani var. eumartii	*Coffea arabica*	plant	Tanzania	USDA
Fusarium sp.	*Coffea arabica*	plant	Brazil, Costa Rica, Cuba, Fiji, Florida, Malawi, Mexico, Panama, Papua New Guinea, Thailand, Venezuela	USDA
Fusarium sp.	*Coffea canephora*	plant	Fiji	USDA
Fusarium sp.	*Coffea liberica*	plant	Brunei Darussalam	USDA
Fusarium sp.	*Coffea robusta*	plant	Brunei Darussalam, Malawi	USDA
Fusarium sp.	*Coffea* sp.	beans, plant	New Guinea, Zimbabwe	USDA
Fusarium stilboides	*Coffea arabica*	bark	Kenya, Malawi, Zimbabwe	USDA
Fusarium stilboides	*Coffea* sp.	beans	Minas Gerais, Brazil (Cerrado and Sul de Minas)	[3]
Fusarium xylarioides	*Coffea abeokutae*	/	Cote d'Ivoire	USDA
Fusarium xylarioides	*Coffea arabica*	plant	Ethiopia, Uganda	USDA
Fusarium xylarioides	*Coffea canephora*	plant	Cote d'Ivoire, Uganda, Guinea	USDA
Fusarium xylarioides	*Coffea liberica*	/	Cote d'Ivoire	USDA
Fusarium xylarioides	*Coffea robusta*	plant	Uganda	USDA
Fusarium xylarioides	*Coffea* sp.	trunk	Cote d'Ivoire, Ivory Coast	USDA
Gibberella sp.	*Coffea arabica*	beans, plant	Colombia, Mexico	USDA, [5]
Gibberella stilboides – (*Fusarium stilboides*)	*Coffea arabica*	/	Papua New Guinea	USDA
Gibberella xylarioides – (*Fusarium xylarioides*)	*Coffea abeokutae*	/	Cote d'Ivoire	USDA
Gibberella xylarioides – (*Fusarium xylarioides*)	*Coffea canephora*	/	Cote d'Ivoire	USDA
Gibberella xylarioides – (*Fusarium xylarioides*)	*Coffea canephora var. robusta*	stems	Congo, Cote d'Ivoire, Guinea	USDA
Gibberella xylarioides – (*Fusarium xylarioides*)	*Coffea canephora var. typica*	/	Guinea	USDA
Gibberella xylarioides – (*Fusarium xylarioides*)	*Coffea dewevrei*	stems	Congo	USDA

	Gibberella xylarioides – (*Fusarium xylarioides*)	*Coffea excelsa*	stems	Congo, Cote d'Ivoire	USDA
	Gibberella xylarioides – (*Fusarium xylarioides*)	*Coffea liberica*	/	Cote d'Ivoire	USDA
	Gibberella xylarioides – (*Fusarium xylarioides*)	*Coffea neoarnoldiana*	stems	Congo	USDA
	Gliocladiopsis tenuis	*Coffea* sp.	/	Viet Nam	USDA
	Lanatonectria flavolanata – (*Sarcopodium flavolanatum*)	*Coffea arabica*	/	India	USDA
	Nectria bainii – (*Clonostachys rosea f. rosea*)	*Coffea arabica*	/	Cuba	USDA
	Nectria cinnabarina	*Coffea* sp.	plant	Malay Peninsula	USDA
	Nectria coffeigena	*Coffea abeokutae*	/	Cote d'Ivoire	USDA
	Nectria coffeigena	*Coffea canephora*	/	Cote d'Ivoire	USDA
	Nectria coffeigena	*Coffea liberica*	/	Cote d'Ivoire	USDA
	Nectria dodgei	*Coffea arabica*	plant	El Salvador	USDA
	Nectria dodgei	*Coffea arabica var. maragogipe*	plant	Guatemala	USDA
	Nectria flavo-lanata – (*Sarcopodium flavolanatum*)	*Coffea arabica*	/	India	USDA
	Nectria sp.	*Coffea arabica*	plant	Costa Rica, El Salvador, Guatemala, Mexico	USDA
	Nectria tropica	*Coffea* sp.	/	Colombia	USDA
	Nectria veuillotiana – (*Thelonectria veuillotiana*)	*Coffea arabica*	crown	Dominican Republic	USDA
	Neocosmospora acutispora	*Coffea arabica*	/	Guatemala	USDA
	Neocosmospora ampla	*Coffea* sp.	/	East Africa	USDA
	Ophionectria foliicola	*Coffea liberica*	leaves	Java	USDA
	Volutella sp.	*Coffea arabica*	/	Mexico, Nicaragua	USDA
Omphalotaceae (6)	*Marasmiellus scandens*	*Coffea arabica*	plant	Fiji, Malaysia	USDA
	Marasmiellus scandens	*Coffea liberica*	plant	Malaysia	USDA
	Marasmiellus scandens	*Coffea robusta*	plant	Brunei Darussalam, Malaysia	USDA
	Marasmius scandens – (*Marasmiellus scandens*)	*Coffea arabica*	plant	Fiji	USDA
	Marasmius scandens –	*Coffea canephora var. typica*	/	Guinea	USDA

	(Marasmiellus scandens)				
	Marasmius scandens – *(Marasmiellus scandens)*	*Coffea liberica*	Plant	Sierra Leone	USDA
Ophiocordycipitaceae (1)	*Purpureocillium lilacinum*	Catuai variety	corticous roots tissues	Piumhi, Brazil	[6]
Orbiliaceae (1)	*Trinacrium indica*	*Coffea arabica*	leaves	India	USDA
Parodiopsidaceae (1)	*Dimerosporina* sp.	*Coffea* sp.	plant	Malaysia	USDA
Patellariaceae (4)	*Tryblidiella rufula* – *(Rhytidhysteron rufulum)*	*Coffea abeokutae*	/	Cote d'Ivoire	USDA
	Tryblidiella rufula – *(Rhytidhysteron rufulum)*	*Coffea canephora*	/	Cote d'Ivoire	USDA
	Tryblidiella rufula – *(Rhytidhysteron rufulum)*	*Coffea liberica*	/	Cote d'Ivoire	USDA
	Tryblidiella rufula var. microspora – *(Triblidium rufulum var. microsporum)*	*Coffea canephora var. typica*	/	Guinea	USDA
Peronosporaceae (2)	*Phytophthora palmivora*	*Coffea arabica*	/	China	USDA
	Phytophthora sp.	*Coffea arabica*	/	Panama	USDA
Pestalotiopsidaceae (13)	*Pestalotiopsis batatas*	*Coffea robusta*	/	China	USDA
	Pestalotiopsis coffeae	*Coffea excelsa*	/	China	USDA
	Pestalotiopsis coffeae-arabicae	*Coffea arabica*	leaves	China	USDA
	Pestalotiopsis disseminata	*Coffea arabica*	/	Ghana	USDA
	Pestalotiopsis elasticae	*Coffea arabica*	/	China	USDA
	Pestalotiopsis funerea	*Coffea arabica*	/	Kenya	USDA
	Pestalotiopsis kenyana	*Coffea* sp.	branches	Kenya	USDA
	Pestalotiopsis maculans	*Coffea arabica*	plant	Brazil	USDA
	Pestalotiopsis royenae	*Coffea* sp.	plant	Mauritius	USDA
	Pestalotiopsis sp.	*Coffea arabica*	plant	Mexico	USDA
	Pestalotiopsis theae – *(Pseudopestalotiopsis theae)*	*Coffea robusta*	plant	Brunei Darussalam	USDA
	Pestalotiopsis versicolor	*Coffea arabica*	plant	Venezuela	USDA
	Pestalotiopsis versicolor	*Coffea liberica*	plant	Brunei Darussalam	USDA
Phacidiaceae (1)	*Ceuthospora coffeicola*	*Coffea arabica*	/	Reunion	USDA

Phaeosphaeriaceae (2)	*Hendersonia coffeae*	*Coffea arabica*	plant	Mexico	USDA
	Hendersonia sp.	*Coffea arabica*	/	Eritrea	USDA
Phanerochaetaceae (3)	*Phanerochaete salmonicolor* – (*Erythricium salmonicolor*)	*Coffea arabica*	/	Papua New Guinea	USDA
	Phanerochaete salmonicolor – (*Erythricium salmonicolor*)	*Coffea canephora*	/	Papua New Guinea	USDA
	Phanerochaete salmonicolor – (*Erythricium salmonicolor*)	*Coffea* sp.	plant	Fiji, Papua New Guinea, Samoa	USDA
Phyllostictaceae (15)	*Guignardia* sp.	*Coffea arabica*	leaves	Puerto Rico	[1]
	Guignardia coffeana	*Coffea arabica*	plant	China, Taiwan	USDA
	Phyllosticta bokensis	*Coffea liberica*	/	Congo, Democratic Republic of the	USDA
	Phyllosticta camoensis	*Coffea camoensis*	leaves	Africa	USDA
	Phyllosticta coffeae-arabicae – (*Phyllosticta bokensis*)	*Coffea arabica*	plant	Taiwan	USDA
	Phyllosticta coffeae-libericae	*Coffea robusta*	/	India	USDA
	Phyllosticta coffeicola	*Coffea abeokutae*	/	Cote d'Ivoire	USDA
	Phyllosticta coffeicola	*Coffea arabica*	/	Brazil, China, Congo, Costa Rica, India, Panama	USDA
	Phyllosticta coffeicola	*Coffea canephora*	plant	Cambodia	USDA
	Phyllosticta coffeicola	*Coffea liberica*	/	Cote d'Ivoire	USDA
	Phyllosticta coffeicola	*Coffea* sp.	plant	Fiji	USDA
	Phyllosticta sp.	*Coffea arabica*	plant	Brazil, Costa Rica, El Salvador, Eritrea, Florida, Guatemala, Mexico, Panama	USDA
	Phyllosticta sp.	*Coffea* sp.	plant	Ghana, Malay Peninsula	USDA
	Phyllosticta capitalensis (*Guignardia mangiferae*)	*Coffea arabica*	Mature and healthy leaves	Brazil	[21]
	Phyllosticta usteri	*Coffea arabica*	/	Brazil, Czechoslovakia, Venezuela	USDA
Physalacriaceae (5)	*Armillaria mellea*	*Coffea arabica*	plant	Congo, Guatemala, Kenya, Mexico, Peru, Tanzania, Uganda, Zimbabwe	USDA

	Armillaria mellea	*Coffea canephora var. robusta*	plant	Congo, West Africa	USDA
	Armillaria mellea	*Coffea* sp.	plant	Ghana, Papua New Guinea	USDA
	Armillaria sp.	*Coffea arabica*	/	Kenya	USDA
	Armillariella mellea – (*Armillaria mellea*)	*Coffea arabica*	plant	Malawi	USDA
Physaridae (3)	*Physarum cinereum*	*Coffea arabica*	/	Kenya	USDA
	Physarum leucopus	*Coffea arabica*	/	India	USDA
	Physarum luteolum	*Coffea arabica*	/	India	USDA
Physciaceae (1)	*Physcia* sp.	*Coffea arabica*	plant	Venezuela	USDA
Plectosphaerellaceae (4)	*Verticillium hemileiae* – (*Lecanicillium muscarium*)	*Coffea arabica*	plant	Brazil	USDA
	Verticillium lecanii – (*Lecanicillium lecanii*)	*Coffea arabica*	/	Papua New Guinea	USDA
	Verticillium lecanii – (*Lecanicillium lecanii*)	*Coffea canephora*	/	Papua New Guinea	USDA.
	Verticillium lecanii – (*Lecanicillium lecanii*)	*Coffea* sp.	/	India	USDA
Pleosporaceae (12)	*Alternaria* sp.	*Coffea arabica*	plant	Mexico	USDA
	Alternaria tenuis – (*Alternaria alternata*)	*Coffea* sp.	/	Southern Africa	USDA
	Alternaria citri	*Coffea arabica*	leaves	Mexico	[22]
	Alternaria alternata	*Coffea arabica*	leaves	/	[25]
	Bipolaris coffeana	*Coffea arabica*	leaves	Kenya	USDA
	Bipolaris tropicalis – (*Curvularia tropicalis*)	*Coffea arabica*	leaves	India	USDA
	Bipolaris zeicola	*Coffea arabica*	plant	Brazil	USDA
	Curvularia prasadii	*Coffea arabica*	/	Kenya	USDA
	Curvularia tropicalis	*Coffea arabica*	leaves	India	USDA
	Drechslera sp.	*Coffea arabica*	plant	Brazil	USDA
	Drechslera sp.	*Catuai variety*	corticous roots tissues	Piumhi, Brazil	[6]
	Drechslera biseptata	*Coffea arabica*	Mature and healthy leaves	Brazil	[21]
Pleurotaceae (1)	*Pleurotus* sp.	*Coffea* sp.	/	Philippines	USDA
Polyporaceae (28)	*Amauroderma subrugosum*	*Coffea* sp.	/	Papua New Guinea	USDA
	Fomes lamaoensis – (*Fomes*	*Coffea abeokutae*	/	Cote d'Ivoire	USDA

lamaensis)				
Fomes lamaoensis – (*Fomes lamaensis)*	*Coffea arabica*	roots	Ethiopia, Indonesia, Kenya, Uganda	USDA
Fomes lamaoensis – (*Fomes lamaensis)*	*Coffea canephora var. robusta*	roots	Indonesia, West Africa	USDA.
Fomes lamaoensis – (*Fomes lamaensis)*	*Coffea liberica*	roots	Cote d'Ivoire, West Africa	USDA
Fomes lignosus	*Coffea arabica*	roots	Brazil, Congo, Ethiopia	USDA
Fomes lignosus	*Coffea canephora var. robusta*	roots	Congo, West Africa	USDA
Fomes lignosus	*Coffea liberica*	roots	Cote d'Ivoire	USDA
Fomes lignosus	*Coffea* sp.	plant	Malay Peninsula, Nigeria	USDA
Fomes noxius – (*Phellinidium noxium)*	*Coffea arabica*	plant	China, Taiwan	USDA
Fomes noxius – (*Phellinidium noxium)*	*Coffea* sp.	plant	Malay Peninsula, Nigeria	USDA
Fomes sp.	*Coffea canephora*	/	Papua New Guinea	USDA
Fomes sp.	*Coffea* sp.	/	Papua New Guinea	USDA
Ganoderma lucidum	*Coffea arabica*	/	India	USDA
Ganoderma philippii	*Coffea arabica*	plant	Brazil	USDA
Ganoderma pseudoferreum – (*Ganoderma philippii)*	*Coffea arabica*	plant	East Indies	USDA
Ganoderma pseudoferreum – (*Ganoderma philippii)*	*Coffea liberica*	plant	Malay Peninsula	USDA
Ganoderma pseudoferreum – (*Ganoderma philippii)*	*Coffea robusta*	plant	Malay Peninsula	USDA
Ganoderma sp.	*Coffea arabica*	/	Papua New Guinea	USDA
Hexagonia discopoda	*Coffea canephora var. typica*	/	Guinea	USDA
Polyporus coffeae	*Coffea abeokutae*	/	Cote d'Ivoire	USDA
Polyporus coffeae	*Coffea liberica*	/	Cote d'Ivoire	USDA
Polyporus coffeae	*Coffea* sp.	/	Papua New Guinea	USDA
Polyporus depauperatus	*Coffea arabica*	/	Puerto Rico, Virgin Islands	USDA
Polyporus rubidus	*Coffea arabica*	roots	Indonesia	USDA
Polyporus rubidus	*Coffea canephora var. robusta*	roots	Indonesia	USDA

	Polyporus zonalis – (*Rigidoporus lineatus*)	*Coffea arabica*	plant	Brazil	USDA
	Trametes cinnabarina – (*Pycnoporus cinnabarinus*)	*Coffea dybowskii*	/	Philippines	USDA
Pythiaceae (7)	*Globisporangium splendens*	*Coffea arabica*	/	Zambia	USDA
	Pythium periilum	*Coffea arabica*	soil	South Africa	USDA
	Pythium sp.	*Coffea arabica*	roots	Florida, Guatemala, Mexico	USDA
	Pythium splendens – (*Globisporangium splendens*)	*Coffea arabica*	soil	South Africa	USDA
	Trachysphaera fructigena	*Coffea abeokutae*	/	Cote d'Ivoire	USDA
	Trachysphaera fructigena	*Coffea canephora*	/	Cote d'Ivoire	USDA
	Trachysphaera fructigena	*Coffea liberica*	fruits	Congo, Democratic Republic of the, Congo, Ghana	USDA
Pseudohalonectriaceae (1)	*Pseudohalonectria lutea*	*Coffea arabica*	leaves	/	[25]
Rhizopodaceae (2)	*Rhizopus oryzae*	*Coffea arabica*	beans	/	[2]
	Rhizopus sp.	Arabica/robusta	beans	Philippines	[31]
Roussoellaceae (1)	*Roussoella mexicana*	*Coffea arabica*	leaves	Mexico	USDA
Saccharomycetaceae (3)	*Saccharomyces ellipsoideus*	*Coffea arabica*	/	Puerto Rico, Virgin Islands	USDA
	Hanseiaspora valbyensiskloecker	*Coffea* sp.	coffee waste	Ethiopia (Sidama and Gedio)	[32]
	Pichia amenthionina var. menthionina	*Coffea* sp.	coffee waste	Ethiopia (Sidama and Gedio)	[32]
Saccotheciaceae (2)	*Metasphaeria* sp.	*Coffea arabica*	/	Eritrea	USDA
	Aureobasidium pullulans	*Coffea arabica*	Mature and healthy leaves	Brazil	[21]
Schizophyllaceae (4)	*Schizophyllum* sp.	*Coffea arabica*	leaves	Brazil	[20]
	Schizophyllum alneum – (*Schizophyllum commune*)	*Coffea arabica*	plant	Brazil	USDA
	Eremotheca coffeana	*Coffea arabica*	plant	Brazil	USDA
	Microthyriella guianensis – (*Schizothyrium longisporum*)	*Coffea liberica*	/	French Guiana	USDA
Sclerotiniaceae (3)	*Botrytis cinerea*	*Coffea arabica*	seedlings, leaves	China, Kenya, Tanzania	USDA

	Botrytis cinerea f. sp. *coffeae*	*Coffea arabica*	/	Papua New Guinea	USDA
	Botryotinia fuckeliana – (*Botrytis cinerea*)	*Coffea arabica*	plant	Hawaii	USDA
Sebacinaceae (1)	*Chaetospermum camelliae*	*Coffea canephora*	/	India	USDA
Seuratiaceae (1)	*Seuratia millardetii* – (*Atichia glomerulosa*)	*Coffea* sp.	leaves	Hawaii	USDA
Sporidiobolaceae (3)	*Rhodotorula hylophila*	*Coffea* sp.	coffee waste	Ethiopia (Sidama and Gedio)	[32]
	Rhodotrula aurantiaca A	*Coffea* sp.	coffee waste	Ethiopia (Sidama and Gedio)	[32]
	Rhodotorula aurantiaca	*Coffea arabica*	Mature and healthy leaves	Brazil	[21]
Stachybotryaceae (4)	*Cymostachys coffeicola*	*Coffea arabica*	/	Cuba	USDA
	Paramyrothecium roridum	*Coffea arabica*	leaves	Mexico	USDA
	Paramyrothecium roridum	*Coffea* sp.	twigs, leaves	Colombia	USDA
	Stachybotrys parvispora	*Coffea liberica*	plant	Malaysia	USDA
Stereaceae (1)	*Aleurodiscus moquiniarum*	*Coffea arabica*	plant	Brazil	USDA
Stictidaceae (2)	*Fitzroyomyces yunnanensis*	*Coffea* sp.	Dead wood	China	[33]
	Ostropomyces pruinosella	*Coffea arabica*	Dead wood	China	[33]
Thelebolaceae (1)	*Zukalina* sp.	*Coffea stenophylla*	plant	Ghana	USDA
Trentepohliaceae (4)	*Cephaleuros mycoidea*	*Coffea liberica*	plant	Sierra Leone, Tanzania	USDA
	Cephaleuros virescens	*Coffea arabica*	/	Papua New Guinea	USDA
	Cephaleuros virescens	*Coffea canephora*	/	Papua New Guinea	USDA
	Cephaleuros virescens	*Coffea* sp.	/	Papua New Guinea	USDA
Trichomeriaceae (8)	*Chaetoscorias vulgare*	*Coffea arabica*	plant	China, Taiwan	USDA
	Neocapnodium tanakae – (*Capnodium tanakae*)	*Coffea arabica*	plant	China, Taiwan	USDA
	Neocapnodium tanakae – (*Capnodium tanakae*)	*Coffea liberica*	plant	China, Taiwan	USDA
	Phragmocapnias asiaticus	*Coffea arabica*	/	Thailand	USDA
	Trichomerium chiangmaiensis	*Coffea arabica*	leaves	Chiang Mai Thailand.	USDA
	Triposporiopsis spinigera	*Coffea arabica*	plant	China, Japan, Taiwan	USDA
	Triposporiopsis spinigera	*Coffea liberica*	plant	China, Taiwan	USDA

	Trichopeltis pulchella	*Coffea robusta*	plant	Malaysia	USDA
Tubeufiaceae (2)	*Helicoma olivaceum*	*Coffea arabica*	/	Mexico	USDA
	Titaea hemileiae	*Coffea arabica*	/	Nigeria	USDA
Ustilaginaceae (1)	*Pseudozyma* sp.	*Coffea arabica*	green coffee seeds	Puerto Rico	[5]
Venturiaceae (1)	*Venturia coffeicola*	*Coffea arabica*	plant	Brazil	USDA
Vizellaceae (1)	*Vizella bingervilliana*	*Coffea liberica*	/	Africa	USDA
Induratiaceae (3)	*Muscodor coffeanum*	*Coffea arabica*	leaf	Brazil, Minas Gerais, Viçosa municipality	[23,23]
	Muscodor vitigenus	*Coffea arabica*	stem	Brazil, Minas Gerais, Viçosa municipality	[23]
	Muscodor yucatanensis	*Coffea arabica*	leaf	Brazil, Minas Gerais, Viçosa municipality	[23,23]
Xylariaceae (29)	*Anthostomella coffeae*	*Coffea liberica*	/	Cote d'Ivoire	USDA
	Dematophora necatrix – (*Rosellinia necatrix*)	*Coffea arabica*	plant	Brazil	USDA
	Rosellinia aquila	*Coffea arabica*	roots	Guatemala	USDA
	Rosellinia arcuata	*Coffea excelsa*	/	Central African Republic	USDA
	Rosellinia arcuata	*Coffea robusta*	/	Central African Republic	USDA
	Rosellinia asperata	*Coffea excelsa*	/	Central African Republic	USDA
	Rosellinia asperata	*Coffea robusta*	/	Central African Republic	USDA
	Rosellinia boukokoae	*Coffea excelsa*	/	Central African Republic	USDA
	Rosellinia boukokoae	*Coffea robusta*	/	Central African Republic	USDA
	Rosellinia bunodes	*Coffea arabica*	roots	Brazil, Colombia, Indonesia, Jamaica, Mexico, Peru, Puerto Rico, Virgin Islands	USDA
	Rosellinia bunodes	*Coffea robusta*	/	Central African Republic	USDA
	Rosellinia bunodes	*Coffea* sp.	plant	Colombia, Malay Peninsula, Trinidad and Tobago	USDA
	Rosellinia coffeae	*Coffea excelsa*	/	Central African Republic	USDA
	Rosellinia coffeae	*Coffea robusta*	/	Central African Republic	USDA
	Rosellinia emergens	*Coffea robusta*	/	Central African Republic	USDA
	Rosellinia lobayensis	*Coffea excelsa*	/	Central African Republic	USDA
	Rosellinia megalospora	*Coffea excelsa*	/	Central African Republic	USDA
	Rosellinia megalospora	*Coffea robusta*	/	Central African Republic	USDA
	Rosellinia necatrix	*Coffea arabica*	/	China, Eritrea	USDA
	Rosellinia pepo	*Coffea arabica*	roots	Brazil, Jamaica	USDA
	Rosellinia pepo	*Coffea* sp.	/	Papua New Guinea	USDA
	Rosellinia saccasii	*Coffea robusta*	/	Central African Republic	USDA
	Rosellinia sp.	*Coffea arabica*	dead hardwood	Brazil, Dominican Republic, Haiti, Mexico	USDA
	Stilbohypoxylon coffeicola	*Coffea arabica*	/	Guadeloupe	USDA
	Stilbohypoxylon	*Coffea robusta*	/	Central Africa	USDA

quisquiliarum				
Subramaniomyces fusisaprophyticus	*Coffea* sp.	dead, fallen leaves	Ethiopia	USDA
Ustulina deusta – (*Kretzschmaria deusta*)	*Coffea arabica*	plant	Fiji	USDA
Xylaria sp.	*Coffea arabica*	leaves	Puerto Rico	[1,25]
Xylaria rhizocola	*Coffea arabica*	/	Colombia	USDA

USDA Indicates the species collectesd from the website: https://www.ars.usda.gov/ for the list of coffee fungi.

Table S2. Frequently found fungal families and genera on coffee.

Family	Genera
Aspergillaceae (116)	*Aspergillus*
Incertae sedis (93)	*Hemileia*
Glomerellaceae (77)	*Colletotrichum*
Nectriaceae (77)	*Fusarium*
Ceratobasidiaceae (39)	*Ceratocystis*
Didymellaceae (34)	*Phoma*
Mycosphaerellaceae (33)	*Cercospora*
Xylariaceae (29)	*Rosellinia*

Table S3. Different life modes of coffee fungi.

**Endophytes（138）**	
Acremonium alternatum	*Muscodor vitigenus*
Acremonium sp.	*Muscodor yucatanensis*
Allophoma nicaraguensis	*Microascus* sp.
Aspergillus tamarii	*Mycosphaerella* sp.
Aspergillus tubingensis	*Nodulisporium gregarium*
Aspergillus aculeatinu	*Ophiognomonia* sp.
Aspergillus alliaceus	*Paecilomyces cf. fumosoroseus*
Aspergillus brasiliensis	*Paecilomyces cf. javanicus*
Aspergillus caespitosus	*Paecilomyces* sp.
Aspergillus clavatus	*Penicillium digitatum*
Aspergillus foetidus	*Penicillium ubiquetum*
Aspergillus fumigatus	*Penicillium aurantiogriseum*
Aspergillus granulosus	*Penicillium brevecompactum*
Aspergillus lanosus	*Penicillium brevicompactum*
Aspergillus niger aggregate	*Penicillium chrysogenum*
Aspergillus niger agregado	*Penicillium citrinum*
Aspergillus niger var. awamor	*Penicillium coffeae*
Aspergillus niger var. niger	*Penicillium commune*
Aspergillus oryzae	*Penicillium corylophilum*
Aspergillus pseudodeflectus	*Penicillium corylophylum*
Aspergillus sclerotiicarbonarius	*Penicillium crustosum*
Aspergillus sumatrense	*Penicillium decumbens*
Aspergillus sydowii	*Penicillium expansum*
Aspergillus parasiticus	*Penicillium glabrum*
Aspergillus terreus	*Penicillium implicatum*
Aureobasidium pullulans	*Penicillium italicum*
Alternaria alternata	*Penicillium jantinelum*
Alternaria citri	*Penicillium montanense*
Botryosphaeria sp.	*Penicillium olsonii*
Beauveria brongniartii	*Penicillium oxalicum*
Beauveria bassiana	*Penicillium pelutatum*
Beauveria globulifera	*Penicillium solitum*
Cephalosporium lecanii - (*Lecanicillium lecanii*)	*Penicillium waksmanii*
Chaetomium globosum	*Pestalotia* sp.
Cladosporium cf.cladosporioides	*Pestalotiopsis maculans*
Cladosporium cf.sphaerospermum	*Periconia* sp.
Cladosporium cladosporioides complex	*Phlebia subserialis*
Cladosporium pseudocladosporioides	*Phoma eupyrena*
Cladosporium tenuissimum	*Phoma herbarum*
Clavicipitaceae sp.	*Phoma exigua var. exigua*
Clonostachys rosea	*Phomopsis arnoldiae*
Colletotrichum sp.	Phyllosticta capitalensis - (*Guignardia mangiferae*)
Colletotrichum falcatum	*Microsphaeropsis* sp.
Colletotrichum gloeosporioides	*Phomopsis stipata*
Colletotrichum kahawae	*Pseudozyma* sp.
Colletotrichum asianum	*Pseudohalonectria lutea*

Colletotrichum crassipes	*Purpureocillium lilacinum*
Colletotrichum aff brassicicola	*Rhizopus oryzae*
Colletotrichum musae	*Rhodotorula aurantiaca*
Coniosporium sp.	*Sarocladium bacillisporum*
Cladosporium cladosporioides	*Schizophyllum* sp.
Diaporthe liquidambaris	*Simplicilium* sp.
Diaporthe phaseoli	*Staninwardia* sp.
Diaporthe yunnanensis	*Trichoderma* sp.
Drechslera biseptata	*Trichoderma atroviride*
Drechslera sp.	*Trichoderma botryosum* sp.
Diplodia sp.	*Trichoderma breve*
Eurotium amstelodami	*Trichoderma caeruloviride* sp.
Eurotium chevalieri	*Trichoderma flagellatum*
Eurotium ruber	*Trichoderma guizhouense*
Eutypella coffeicola	*Trichoderma hamatum*
Fusarium solani	*Trichoderma koningiopsis*
Gibberella sp.	*Trichoderma lentissimum* sp.
Guignardia sp.	*Trichoderma parareesei*
Khuskia oryzae	*Trichoderma pseudopyramidale* sp.
Lichtheimia ramosa	*Trichoderma spirale*
Libertella sp.	*Trichoderma theobromicola*
Leptosphaeria sp.	*Trichoderma virens*
Muscodor coffeanum	*Xylaria* sp.
**Pathogens（295）**	
Acrostalagmus aphidum - (*Lecanicillium longisporum*)	*Hemileia coffeicola*
Aecidium travancoricum	*Hemileia vastatrix*
Aithaloderma longisetum	*Hendersonia coffeae*
Aithaloderma setosum	*Hymenochaete noxia*
Alternaria sp.	*Hypoxylon deustum* - (*Kretzschmaria deusta*)
Alternaria tenuis - (*Alternaria alternata*)	*Hypoxylon stygium* - (*Annulohypoxylon stygium*)
Ambrosiella xylebori	*Isariopsis acanthacearum*
Annellophora sp.	*Irenina coffeae*
Antennaria setosa	*Irenina glabra*
Anthostoma sp.	*Irpex flavus* - (*Flavodon flavus*)
Anthostomella coffeae	*Koleroga noxia* - (*Rhizoctonia noxia*)
Armillaria mellea	*Laestadia coffeicola*
Armillaria sp.	*Lasiodiplodia pseudotheobromae*
Armillariella mellea - (*Armillaria mellea*)	*Lasiodiplodia theobromae*
Aschersonia sp.	*Leptoporus lignosus*
Ascochyta coffeae - (*Boeremia exigua* var. *coffeae*)	*Leptosphaeria canephorae*
Ascochyta sp.	*Leptosphaeria coffaeicida*
Ascochyta tarda - (*Boeremia exigua coffeae*)	*Leptosphaeria coffeicola*
Athelia rolfsii	*Leptosphaeria coffeigena*
Auerswaldia excoriata	*Leptosphaeria coffeigena var. longirostrata*
Bipolaris coffeana	*Leptosphaeria pusilla*
Biscogniauxia sp.	*Leptosphaeria tonduzi*
Boeremia exigua var. *coffeae*	*Limacinia* sp.
Boeremia exigua var. *heteromorpha*	*Macrophomina phaseoli*
Botryodiplodia theobromae - (*Lasiodiplodia theobromae*)	*Macrophomina phaseolina*

Botryotinia fuckeliana - (*Botrytis cinerea*)
Botrytis cinerea
Beltrania rhombica
Candida albicans
Capnodium brasiliense
Capnodium coffeae
Capnodium coffeicola
Capnodium sp.
Cephaleuros mycoidea
Cephalosporium deformans
Cephalosporium omnivorum
Cephalosporium sp.
Cephalosporium zonatum - (*cremonium zonatum*)
Cercospora sp.
Ceratobasidium noxium
Ceratobasidium sp.
Ceratocystis colombiana
Ceratocystis fimbriata
Ceratocystis papillata
Ceratocystis paradoxa - (*Thielaviopsis paradoxa*)
Ceratostomella fimbriata
Cercospora coffeae
Cercospora coffeicola
Cercospora herrerana
Chaetoscorias vulgare
Chaetostroma sp.
Chalara neocaledoniae - (*Davidsoniella neocaledoniae*)
Choanephora conjuncta - (*Choanephora infundibulifera*)
Cladosporium hemileiae
Cladosporium herbarum
Cladosporium oxysporum
Cladosporium sp.
Cladosporiella deightonii
Clitocybe tabescens - (*Desarmillaria tabescens*)
Colletotrichum acutatum
Colletotrichum boninense
Colletotrichum brevisporum
Colletotrichum capsici - (*Colletotrichum truncatum*)
Colletotrichum cereale
Colletotrichum coffeanum
Colletotrichum coffeophilum
Colletotrichum costaricense
Colletotrichum dematium
Colletotrichum endophyticum
Colletotrichum fragariae - (*Colletotrichum theobromicola*)
Colletotrichum fructicola
Colletotrichum gigasporum
Colletotrichum gloeosporioides
Colletotrichum kahawae

Macrophoma corchori - (*Macrophomina phaseolina*)
Marasmiellus scandens
Marasmius equicrinis
Marasmius scandens
Marasmius sp.
Microdiplodia sp.
Micropeltella mulleri
Micropeltis applanata
Micropeltis mucosa
Microxyphium sp.
Mycena citricolor
Mycosphaerella coffeae
Mycosphaerella coffeicola
Myrothecium advena - (*Paramyrothecium roridum*)
Myrothecium roridum - (*Paramyrothecium roridum*)
Myrothecium sp.
Nectria cinnabarina
Nectria coffeigena
Nectria dodgei
Nectria sp.
Nematospora coryli - (*Eremothecium coryli*)
Nematospora gossypii - (*Eremothecium gossypii*)
Neocapnodium tanakae - (*Capnodium tanakae*)
Neofusicoccum parvum
Nigrospora oryzae
Nigrospora sp.
Nigrospora sphaerica
Omphalia flavida - (*Mycena citricolor*)
Ophionectria foliicola
Oraniella coffeicola - (*Massarina coffeicola*)
Paracapnodium brasilense
Paramyrothecium roridum
Pellicularia filamentosa - (*Rhizoctonia solani*)
Pellicularia koleroga - (*Rhizoctonia noxia*)
Pellicularia sp.
Pestalotia coffeae
Pestalotia coffeicola
Pestalotia sp.
Pestalotiopsis coffeae-arabicae
Pestalotiopsis elasticae
Pestalotiopsis kenyana
Pestalotiopsis royenae
Pestalotiopsis sp.
Pestalotiopsis theae
Pestalotiopsis versicolor
Pestalozzia coffeae - (*Pestalotia coffeae*)
Peyronellaea coffeae-arabicae
Phaeosaccardinula javanica - (*Limacinula javanica*)
Phanerochaete salmonicolor - (*Erythricium salmonicolor*)

*Colletotrichum kahawae sub*sp. *kahawae* - (*Colletotrichum kahawae*)
Colletotrichum karstii - (*Colletotrichum karsti*)
Colletotrichum ledongense
Colletotrichum magnisporum
Colletotrichum plurivorum
Colletotrichum queenslandicum
Colletotrichum siamense
Colletotrichum theobromicola
Colletotrichum tropicale
Colletotrichum vietnamense
Colletotrichum walleri
Coniothyrium coffeae
Coniothyrium fuckelii - (*Paraconiothyrium fuckelii*)
Coniothyrium sp.
Corticium koleroga - (*Rhizoctonia noxia*)
Corticium salmonicolor - (*Erythricium salmonicolor*)
Corticium solani - (*Rhizoctonia solani*)
Corynespora cassiicola
Curvularia prasadii
Curvularia tropicalis
Cylindrocarpon sp.
Cylindrocarpon tenue
Cylindrocladiella parva
Davidsoniella neocaledoniae
Diaporthe acutispora
Diaporthe sp.
Dictyothyriella mucosa - (*Micropeltis mucosa*)
Didymella coffeae-arabicae
Didymosphaeria sp.
Dimerosporina sp.
Diplodia coffeicola
Ectophoma pomi
Epicoccum sorghi
Eriomycopsis sp.
Erythricium salmonicolor
Fomes lamaoensis - (*Fomes lamaensis*)
Fomes lignosus
Fomes noxius - (*Phellinidium noxium*)
Fusarium camptoceras
Fusarium coffeicola
Fusarium decemcellulare
Fusarium equiseti
Fusarium lateritium
Fusarium lateritium var. longum
Fusarium oxysporum
Fusarium oxysporum f. conglutinans
Fusarium semitectum – (*Fusarium incarnatum*)
Fusarium solani – (*Neocosmospora solani*)
Phellinus lamaensis
Phellinus noxius - (*Phellinidium noxium*)
Phoma coffeae-arabicae - (*Didymella coffeae-arabicae*)
Phoma coffeicola
Phoma costarricensis - (*Phoma costaricensis*)
Phoma costarricensis
Phoma leveillei
Phoma pereupyrena
Phoma sorghina
Phoma sp.
Phoma tarda - (*Boeremia exigua var. coffeae*)
Phomopsis coffeae
Phomopsis heveicola
Phomopsis sp.
Phyllosticta bokensis
Phyllosticta camoensis
Phyllosticta coffeae-arabicae - (*Phyllosticta bokensis*)
Phyllosticta coffeae-libericae
Phyllosticta coffeicola
Phyllosticta sp.
Phyllosticta usteri
Physalospora sp.
Phytophthora palmivora
Polystictus phaeus
Podosporium sp.
Polyporus coffeae
Polyporus depauperatus
Polyporus rubidus
Polyporus zonalis - (*Rigidoporus lineatus*)
Polystictus occidentalis
Pyrenochaetopsis microspora
Pyrrhoderma noxium - (*Phellinidium noxium*)
Pythium periilum
Pythium sp.
Pythium splendens
Rhizoctonia bataticola - (*Macrophomina phaseolina*)
Rhizoctonia choussii
Rhizoctonia lamellifera - (*Macrophomina phaseolina*)
Rhizoctonia solani
Rhizoctonia sp.
Rigidoporus lignosus - (*Rigidoporus microporus*)
Rigidoporus microporus
Rosellinia aquila
Rosellinia bunodes
Rosellinia lobayensis
Rosellinia necatrix
Rosellinia pepo
Rosellinia sp.

Fusarium solani var. eumartii	*Roussoella mexicana*
Fusarium sp.	*Sclerotium coffeicola*
Fusarium stilboides	*Sclerotium rolfsii* - (*Athelia rolfsii*)
Fusarium xylarioides	*Sclerotium* sp.
Fusicoccum sp.	*Scolecopeltis longispora*
Ganoderma lucidum	*Scorias communis*
Ganoderma philippii	*Septoria berkeleyi*
Ganoderma pseudoferreum - (*Ganoderma philippii*)	*Septoria coffeae*
Geotrichum candidum	*Septoria coffeicola*
Gibberella stilboides	*Sphaerella coffeicola* - (*Cercospora coffeicola*)
Gibberella xylarioides - (*Fusarium xylarioides*)	*Sphaerella* sp.
Gliocladiopsis tenuis	*Stilbella flavidum*
Globisporangium splendens	*Stilbum flavidum* - (*Mycena citricolor*)
Gloeosporium coffeanum	*Stenella coffeae*
Gloeosporium coffeicola	*Thanatephorus cucumeris* - (*Rhizoctonia solani*)
Gloeosporium sp.	*Thielaviopsis neocaledoniae* - (*Davidsoniella neocaledoniae*)
Glomerella cingulata - (*Colletotrichum gloeosporioides*)	*Thielaviopsis paradoxa*
Glomerella coffeicola	*Trachysphaera fructigena*
Glomerella phomoides	*Trichopeltis pulchella*
Guignardia coffeana	*Trichothecium roseum*
Helicobasidium compactum	*Tripospermum gardneri*
Helicobasidium longisporum	*Triposporiopsis spinigera*
Helminthosporium coffeae	*Ustulina deusta* - (*Kretzschmaria deusta*)
Hirneola polytricha - (*Auricularia cornea*)	*Venturia coffeicola*
Helminthosporium sp.	*Vermicularia* sp.
Helminthosporium ubangiense	
**Post-harvest disease fungi （26）**	
Aspergillus auricomus	*Aspergillus petrakii*
Aspergillus awamori	*Aspergillus sclerotiorum*
Aspergillus candidus	*Aspergillus* sp.*seção Circumdati*
Aspergillus carbonarius	*Aspergillus* sp.
Aspergillus elegans	*Aspergillus steynii*
Aspergillus flavus	*Aspergillus sulphureus*
Aspergillus insulicola	*Aspergillus westerdijkiae*
Aspergillus japonicus	*Fusarium* sp.
Aspergillus melleus	*Mucor* sp.
Aspergillus niger	*Penicillium* sp.
Aspergillus ochraceus	*Penicillium verruculosum*
Aspergillus ostianus	*Rhizopus* sp.
Aspergillus versicolor	*Byssochlamys Spectabilis*
**Sarobes(30)**	
Acaulospora flava	*Leptosphaeria macrorostra*
Akanthomyces johnsonii	*Leptosphaeria oubanguiensis*
Cylindrocladium scoparium - (*Calonectria cylindrospora*)	*Phaeosaccardinula coffeicola*
Dacryopinax spathularia	*Phialocephala Mexicana*
Hanseiaspora valbyensiskloecker	*Physcia* sp.
Hyphopichia burtonii A	*Phaeodothis winteri*
Fitzroyomyces yunnanensis	*Pichia amenthionina var. menthionina*
Leptosphaeria africana	*Phragmocapnias asiaticus*

Leptosphaeria centrafricana	*Phytophthora* sp.
Leptosphaeria cylindrospora	*Rhodotorula hylophila*
Leptosphaeria excelsa	*Rhodotrula aurantiaca*
Leptosphaeria gigaspora - (*Wettsteinina niesslii*)	*Subramaniomyces fusisaprophyticus*
Leptosphaeria lobayensis	*Ostropomyces pruinosella*
Leptosphaeria tetraspora	*Pseudocercospora coffeigena*
Leptosphaeria longispora - (*Ophiobolus feltgenii*)	*Trichomerium chiangmaiensis*
**Unknown（159）**	
Aaosphaeria arxii	*Macrophoma coffeae var. macrospora*
Aecidium nobile	*Melanochaeta hemipsila*-(*Sporoschisma hemipsilum*)
Aleurodiscus moquiniarum	*Meliola coffeae*
Amauroderma subrugosum	*Meliola psychotriae*
Arthrinium arundinis	*Meliola psychotriae var. coffeae*
Arthrinium phaeospermum	*Meliola rogeri*
Arxiella terrestris	*Meliola* sp.
Aschersonia coffeae - (*Moelleriella javanica*)	*Metasphaeria* sp.
Aspergillus glaucus	*Micropeltidium tonduzii*
Asteridiella naucleae var. libericae	*Microthyriella guianensis* - (*Schizothyrium longisporum*)
Asterina coffeicola	*Microxiphium coffeanum*
Auricularia polytricha - (*Auricularia cornea*)	*Munkovalsaria donacina*
Ascospora coffeae	*Nectria bainii* - (*Clonostachys rosea f. rosea*)
Auricularia delicata - (*Laschia delicata*)	*Nectria flavo-lanata* - (*Sarcopodium flavolanatum*)
Bahusutrabeeja dwaya	*Nectria tropica*
Bipolaris tropicalis - (*Curvularia tropicalis*)	*Nectria veuillotiana* - (*Thelonectria veuillotiana*)
Bipolaris zeicola	*Neocosmospora acutispora*
Botryodiplodia sp.	*Neocosmospora ampla*
Botrytis cinerea f. sp. *coffeae*	*Paraconiothyrium brasiliense*
Calonectria sp.	*Parapeltella coffeicola*
Calonectria pyrochroa	*Pellicularia isabellina* - (*Botryohypochnus isabellinus*)
Camposporium antennatum	*Penicillium Funiculosum*
Capnodium citri - (*Aithaloderma citri*)	*Penicillium Variable*
Caryospora coffeae	*Penicillium Rugulosum*
Cephaleuros virescens	*Periconia byssoides*
Ceramothyrium coffeanum	*Peroneutypa multistromata*
Cercospora coffeae-olivaceae	*Pestalotia albomaculans*
Ceuthospora coffeicola	*Pestalotia elasticae*
Chaetospermum camelliae	*Pestalotiopsis batatas*
Chaetothyrium boedijnii	*Pestalotiopsis coffeae*
Chaetothyrium setosum	*Pestalotiopsis disseminata*
Clypeolum megalosporium	*Pestalotiopsis funerea*
Corticium sp.	*Phaeosaccardinula* sp.
Coniochaeta ligniaria	*Phoma coffeae*
Coniochaeta velutina	*Physarum cinereum*
Cymostachys coffeicola	*Physarum leucopus*
Dactylaria haptospora	*Physarum luteolum*
Dematophora necatrix - (*Rosellinia necatrix*)	*Plenotrichopsis coffeae*
Deslandesia ficina	*Pleurotus* sp.
Deslandesia javanica - (*Limacinula javanica*)	*Podoxyphium* sp.
Diacanthodes novoguineensis	*Pseudomorfea coffeae*

Diachea leucopodia	*Pyrenochaetopsis setosissima*
Didymostilbe coffeae	*Rosellinia arcuata*
Dimerosporium coronatum	*Rosellinia asperata*
Dinemasporium coffeanum	*Rosellinia boukokoae*
Diplocladiella scalaroides	*Rosellinia coffeae*
Diplodina sp.	*Rosellinia emergens*
Endocalyx melanoxanthus	*Rosellinia megalospora*
Entomophthora fresenii	*Rosellinia saccasii*
Eremotheca coffeana	*Saccharomyces ellipsoideus*
Fomes sp.	*Schizophyllum alneum* - (*Schizophyllum commune*)
Fumago vagans	*Scolecopeltella longispora*
Fusarium acuminatum	*Scolecopeltidium coffeae*
Fusarium concolor	*Scolecopeltis aeruginea*
*Fusarium lateritium sub*sp. *lateritium*	*Scorias* sp.
Fusarium moniliforme	*Septoria* sp.
Fusarium oxysporum f. sp. *coffeae*	*Seuratia millardetii* - (*Atichia glomerulosa*)
Fusarium pallidoroseum - (*Fusarium incarnatum*)	*Spegazzinia meliolae*
Fusarium roseum	*Sphaerotheca coffeaeicola*
Ganoderma sp.	*Sphaerulina coffeicola*
Gigaspora margarita	*Sporidesmium coffeicola*
Gliocladium catenulatum - (*Clonostachys rosea f. catenulata*)	*Stachybotrys parvispora*
Gloeosporium coffeicola var. ramulicola	*Stachylidium bicolor*
Graphis sp.	*Stilbella* sp.
Helicoma olivaceum	*Stilbohypoxylon coffeicola*
Helminthosporium canephorae	*Stilbohypoxylon quisquiliarum*
Helminthosporium glabroides	*Titaea hemileiae*
Hendersonia sp.	*Trametes cinnabarina* - (*Pycnoporus cinnabarinus*)
Hermatomyces tucumanensis	*Trinacrium indica*
Hexagonia discopoda	*Triposporium* sp.
Hymenochaete coffeana	*Tryblidiella rufula* - (*Rhytidhysteron rufulum*)
Hypocrella scutata – (*Hypocrella olivacea*)	*Tryblidiella rufula var. microspora* -(*Triblidium rufulum var. microsporum*)
Hypocrella sp.	*Verticillium hemileiae* - (*Lecanicillium muscarium*)
Hypomyces haematococcus - (*Neocosmospora haematococca*)	*Verticillium lecanii* - (*Lecanicillium lecanii*)
Hypomyces haematococcus var. breviconus -(*Neocosmospora brevicona*)	*Vizella bingervilliana*
Hypocrea lixii	*Volutella* sp.
Hypocrea virens	*Xylaria rhizocola*
Imimyces densus	*Zasmidium coffeae*
Lanatonectria flavolanata - (*Sarcopodium flavolanatum*)	*Zukalina* sp.
Leptothyrium discoideum	

Table S4. True pathogens, postharvest pathogens and other fungi reported on coffee.

Pathogen	Current name	Family	Disease
Acrostalagmus aphidum - (*Lecanicillium longisporum*) UNK	*Acrostalagmus aphidum* - (*Lecanicillium longisporum*)	Hypocreaceae	This fungus is usually parasitic on aphids and can cause damage to many plants (including coffee).
Aecidium travancoricum TP	*Aecidium travancoricum*	Incertae sedis	Coffee leaf rust;
Aithaloderma longisetum UNK	*Aithaloderma longisetum*	Capnodiaceae	Duke (1929)mentioned it attacks coffee, but does not mention its symptoms.
Aithaloderma setosum UNK	*Chaetothyrium setosum*	Capnodiaceae	Sooty mold;
Alternaria sp. TP	/	Pleosporaceae	Coffee leaf spot, corky-root disease;
Alternaria tenuis TP	*Alternaria alternata*	Pleosporaceae	Necrotic spots on coffee leaves;
Ambrosiella xylebori TP	*Ambrosiella xylebori*	Ceratocystidaceae	This fungus associated with ambrosia beetles, it can attack coffee plants and cause death;
Annellophora sp. UNK	/	Incertae sedis	Leaf spots;
Antennaria setosa TP	*Chaetothyrium setosum*	Metacapnodiaceae	Coffee leaf diseases;
Anthostoma sp. UNK	/	Diatrypaceae	Cortical cankers;
Anthostomella coffeae TP	/	Xylariaceae	Coffee die-back of twigs;
Armillaria mellea TP	*Armillaria mellea*/*Armillaria novae-zelandiae*/*Armillaria limonea*	Physalacriaceae	Coffee shoes string root rot;
Armillaria sp. UNK	/	Physalacriaceae	Root rot;
Armillariella mellea TP	*Armillaria mellea*/*Armillaria novae-zelandiae*/*Armillaria limonea*	Physalacriaceae	Coffee shoes string root rot;
Aschersonia sp. TP	/	Clavicipitaceae	This pathogen attacks coffee;
Ascochyta coffeae - (*Boeremia exigua var. coffeae*) TP	*Boeremia coffeae*	Didymellaceae	Coffee canker, brown leaf spots, stem rot, and shoot dieback;
Ascochyta sp. TP	/	Didymellaceae	Coffee leaf blight and stem die-back;
Ascochyta tarda - (*Boeremia exigua var. coffeae*) TP	*Ascochyta tarda*	Didymellaceae	Coffee leaf blight and stem die-back;
Athelia rolfsii TP	*Athelia rolfsii*	Atheliaceae	Coffee leaf blight;
Auerswaldia excoriata TP	*Auerswaldia excoriata*	Dothideaceae	Coffee root rots;
Bipolaris coffeana TP	*Bipolaris coffeana*	Pleosporaceae	Coffee leaf spot;
Biscogniauxia sp. UNK	/	Graphostromataceae	Charcoal canker;
Boeremia exigua var. coffeae TP	*Boeremia coffeae*	Didymellaceae	Coffee canker, leaf spots, stem rot, shoot dieback;
Boeremia exigua var. heteromorpha TP	*Boeremia heteromorpha*	Didymellaceae	Necroses, blights and rots;
Botryodiplodia theobromae UNK	*Lasiodiplodia theobromae*	Incertae sedis	This is a pathogen attacking many plant;
Botryotinia fuckeliana - (*Botrytis cinerea*) TP	*Botryotinia fuckeliana*	Sclerotiniaceae	Coffee gray mold;

Beltrania rhombica UNK	*Beltrania rhombica*	Beltraniaceae	Leaf spot;
Botrytis cinerea TP	*Botrytis cinerea*	Sclerotiniaceae	Coffee gray mold;
Candida albicans PP	*Candida albicans*	Incertae sedis	This grows on coffee powder, it can cause infections of the mucosa (oral/vaginal thrush) in healthy individuals and (rarely) infections of the skin or nails.
Capnodium brasiliense TP	*Scorias brasiliensis*	Capnodiaceae	Coffee Sooty mold;
Capnodium coffeae TP	*Capnodium coffeae*	Capnodiaceae	Coffee Sooty mold;
Capnodium coffeicola TP	*Capnodium coffeicola*	Capnodiaceae	Coffee Sooty mold;
Capnodium sp. TP	/	Capnodiaceae	Coffee Sooty mold;
Cephaleuros mycoidea TP	/	Trentepohliaceae	Coffee leaves and stem white thread;
Cephalosporium deformans UNK	*Cephalosporium deformans*	Incertae sedis	Leaves wilt, canker;
Cephalosporium omnivorum UNK	/	Incertae sedis	Sunflower wilt;
Cephalosporium sp. TP	/	Incertae sedis	Coffee brown leaf spot;
Cephalosporium zonatum TP	*Acremonium zonatum*	Incertae sedis	Coffee pale-brown leaf spots with dark-brown rings, canker;
Ceratobasidium noxium TP	*Rhizoctonia noxia*	Ceratobasidiaceae	Coffee white-thread blight and black rot;
Ceratobasidium sp. TP	/	Chaetothyriaceae	Coffee white thread blight;
Ceratocystis colombiana TP	*Ceratocystis colombiana*	Chaetothyriaceae	Coffee canker;
Ceratocystis fimbriata TP	*Ceratocystis fimbriata*	Chaetothyriaceae	Coffee root rot, canker and vascular wilts;
Ceratocystis papillata TP	*Ceratocystis papillata*	Chaetothyriaceae	Coffee canker;
Ceratocystis paradoxa - (*Thielaviopsis paradoxa*) TP	*Ceratocystis paradoxa*	Chaetothyriaceae	Coffee blight and black rot disease;
Ceratostomella fimbriata TP	*Ceratocystis fimbriata*	Boliniaceae	Coffee canker/black rot;
Cercospora coffeae TP	*Cercospora coffeicola*	Mycosphaerellaceae	Coffee brown-eye leaf spot/berry blotch;
Cercospora coffeicola TP	*Cercospora coffeicola*	Mycosphaerellaceae	Coffee brown-eye leaf spot/berry blotch;
Cercospora herrerana TP	*Cercospora coffeicola*	Mycosphaerellaceae	Coffee brown leaf spots;
Cercospora sp. TP	/	Mycosphaerellaceae	Coffee brown-eye leaf spot/berry blotch;
Chaetoscorias vulgare UNK	/	Trichomeriaceae	Sooty mold;
Chaetostroma sp. TP	/	Incertae sedis	Coffee leaves disease;
Chalara neocaledoniae UNK	*Davidsoniella neocaledoniae*	Choanephoraceae	Vascular wilt disease;
Choanephora conjuncta UNK	*Choanephora infundibulifera*	Choanephoraceae	Blossom blight /leaf blight/twig blight;
Cladosporium hemileiae TP	*Digitopodium hemileiae*	Cladosporiaceae	Coffee leaf rust;
Cladosporium herbarum PP	*Cladosporium herbarum*	Cladosporiaceae	This grows on dead plant, normally it's a saprobes fungus, but it can cause black mold to living leaves, shoots and young seedlings on coffee.
Cladosporium oxysporum TP	*Cladosporium oxysporum*	Cladosporiaceae	Coffee leaf blight;
Cladosporium sp. UNK	/	Cladosporiaceae	This fungi commonly found on dead plant, but it

			produces dark olive colonies and is even black on some substrates
Cladosporiella deightonii TP	*Cladosporiella deightonii*	Incertae sedis	Coffee leaf spot;
Clitocybe tabescens UNK	*Desarmillaria tabescens*	Physalacriaceae	Root rot;
Colletotrichum acutatum TP	*Colletotrichum acutatum*	Glomerellaceae	Coffee anthracnose;
Colletotrichum boninense TP	*Colletotrichum boninense*	Glomerellaceae	Coffee anthracnose;
Colletotrichum brevisporum TP	*Colletotrichum brevisporum*	Glomerellaceae	Coffee anthracnose;
Colletotrichum capsici TP	*Colletotrichum truncatum*	Glomerellaceae	Coffee anthracnose;
Colletotrichum cereale TP	*Colletotrichum cereale*	Glomerellaceae	Coffee rot anthracnose;
Colletotrichum coffeanum TP	*Colletotrichum coffeanum*	Glomerellaceae	Coffee leaf blight/berry rot;
Colletotrichum coffeophilum TP	*Colletotrichum gloeosporioides*	Glomerellaceae	Coffee anthracnose;
Colletotrichum costaricense TP	*Colletotrichum costaricense*	Glomerellaceae	Coffee anthracnose;
Colletotrichum dematium TP	*Colletotrichum dematium*	Glomerellaceae	Coffee anthracnose;
Colletotrichum endophyticum TP	*Colletotrichum endophyticum*	Glomerellaceae	Coffee anthracnose;
Colletotrichum fragariae - (*Colletotrichum theobromicola*) TP	*Colletotrichum fragariae*	Glomerellaceae	Coffee anthracnose;
Colletotrichum fructicola TP	*Colletotrichum fructicola*	Glomerellaceae	Coffee anthracnose;
Colletotrichum gigasporum TP	*Colletotrichum gigasporum*	Glomerellaceae	Coffee anthracnose;
Colletotrichum gloeosporioides TP	*Colletotrichum gloeosporioides*	Glomerellaceae	Coffee anthracnose;
Colletotrichum kahawae TP	*Colletotrichum kahawae*	Glomerellaceae	Coffee berry disease;
Colletotrichum kahawae subsp. *kahawae* - (*Colletotrichum kahawae*) TP	/	Glomerellaceae	Coffee berry disease;
Colletotrichum karstii - (*Colletotrichum karsti*) TP	/	Glomerellaceae	Coffee anthracnose;
Colletotrichum ledongense TP	/	Glomerellaceae	Coffee anthracnose;
Colletotrichum magnisporum TP	*Colletotrichum magnisporum*	Glomerellaceae	Coffee anthracnose;
Colletotrichum plurivorum TP	*Colletotrichum plurivorum*	Glomerellaceae	Coffee anthracnose;
Colletotrichum queenslandicum TP	*Colletotrichum queenslandicum*	Glomerellaceae	Coffee anthracnose;
Colletotrichum siamense TP	*Colletotrichum siamense*	Glomerellaceae	Coffee berry disease;

Colletotrichum theobromicola TP	/	Glomerellaceae	Coffee anthracnose;
Colletotrichum tropicale TP	*Colletotrichum tropicale*	Glomerellaceae	Coffee fruit rots;
Colletotrichum vietnamense TP	*Colletotrichum vietnamense*	Glomerellaceae	Coffee anthracnose;
Colletotrichum walleri TP	*Colletotrichum walleri*	Glomerellaceae	Coffee anthracnose;
Coniothyrium coffeae TP	*Coniothyrium coffeae*	Coniothyriaceae	This is a pathogen attacking the coffee berry during ripening and healthy leaves;
Coniothyrium fuckelii TP	*Paraconiothyrium fuckelii*	Coniothyriaceae	Coffee blight/canker/root rot;
Coniothyrium sp. TP	/	Coniothyriaceae	Coffee blight/canker/root rot;
Corticium koleroga - (*Rhizoctonia noxia*) TP	*Corticium koleroga*	Corticiaceae	Coffee thread blight;
Corticium salmonicolor TP	*Erythricium salmonicolor*	Corticiaceae	Coffee pink disease;
Corticium solani TP	*Rhizoctonia solani*	Corticiaceae	Coffee wilting/brown necrosis of the lower taproot and death of the seedlings disease;
Corynespora cassiicola TP	*Corynespora cassiicola*	Corynesporascaceae	Coffee leaf and berry spot;
Curvularia prasadii UNK	*Curvularia prasadii*	Pleosporaceae	The article just mentions it attacks arabica coffee plants, but does not mention its symptoms; James A. Duke 1897
Curvularia tropicalis UNK	*Curvularia tropicalis*	Pleosporaceae	Leaf blight;
Cylindrocarpon sp. UNK	/	Nectriaceae	Black root disease;
Cylindrocarpon tenue UNK	*Gliocladiopsis tenuis*	Nectriaceae	Root rot disease;
Cylindrocladiella parva UNK	*Cylindrocladiella parva*	Nectriaceae	Dieback, black root;
Davidsoniella neocaledoniae UNK	*Davidsoniella neocaledoniae*	Ceratocystidaceae	Black wood disease;
Diaporthe acutispora TP	*Diaporthe acutispora*	Diaporthaceae	Root and fruit rots, dieback, stem cankers, leaf spots, leaf and pod blights and seed decay on coffee;
Diaporthe sp. TP	/	Diaporthaceae	Root and fruit rots, dieback, stem cankers, leaf spots, leaf and pod blights and seed decay on coffee;
Dictyothyriella mucosa - (*Micropeltis mucosa*) TP	*Dictyothyriella mucosa*	Micropeltidaceae	Plant disease to form small, black, superficial fruiting bodies on coffee;
Didymella coffeae-arabicae TP	*Didymella coffeae arabicae*	Didymellaceae	Coffee canker and dieback diseases;
Didymosphaeria sp. UNK	/	Didymellaceae	Canker and wood rot;
Dimerosporina sp. UNK	/	Parodiopsidaceae	Leaves disease;
Diplodia coffeicola TP	*Diplodia coffeicola*	Botryosphaeriaceae	Coffee cryptogamic disease;
Ectophoma pomi TP	*Ectophoma pomi*	Incertae sedis	Coffee leaf spot;
Epicoccum sorghi UNK	*Epicoccum sorghinum*	Didymellaceae	Leaf spot, Stem necrosis, fruit rotting;
Eriomycopsis sp. UNK	/	Incertae sedis	Leaves disease;
Erythricium salmonicolor TP	*Erythricium salmonicolor*	Corticiaceae	Coffee pink disease;

Fomes lamaoensis - (*Fomes lamaensis*) TP	*Pyrrhoderma lamaoense*	Polyporaceae	Coffee brown root disease;
Fomes lignosus TP	*Rigidoporus microporus*	Polyporaceae/Meripilaceae	Coffee white root rot;
Fomes noxius - (*Phellinidium noxium*) TP	*Pyrrhoderma noxium*	Polyporaceae	Coffee brown root disease;
Fusarium camptoceras TP	*Fusarium camptoceras*	Nectriaceae	Coffee fruit rot, root wilt;
Fusarium coffeicola TP	/	Nectriaceae	Coffee leaves and petioles disease;
Fusarium decemcellulare UNK	*Albonectria rigidiuscula*	Nectriaceae	Many host fruit disease;
Fusarium equiseti TP	*Fusarium equiseti*	Nectriaceae	Coffee wilt disease;
Fusarium lateritium TP	*Fusarium lateritium*	Nectriaceae	Coffee *Fusarium* bark disease;
Fusarium lateritium var. longum TP	*Fusarium stilboides*	Nectriaceae	Coffee bark disease;
Fusarium oxysporum TP	*Fusarium oxysporum*	Nectriaceae	This is a pathogen associated with coffee wilt and corky- roots disease;
Fusarium oxysporum f. conglutinans UNK	/	Nectriaceae	Plant wilt;
Fusarium semitectum UNK	*Fusarium incarnatum*	Nectriaceae	Leaf spot, root rot;
Fusarium solani - (*Neocosmospora solani*) TP	*Fusarium solani*	Nectriaceae	Coffee wilt disease;
Fusarium solani var. eumartii TP	*Fusarium solani*	Nectriaceae	Coffee wilt disease;
Fusarium sp. TP	/	Nectriaceae	This is a pathogen associated with coffee canker, wilt, root disease;
Fusarium stilboides TP	*Fusarium stilboides*	Nectriaceae	Coffee bark disease;
Fusarium xylarioides TP	*Fusarium xylarioides*	Nectriaceae	This is a pathogen associated with coffee wilt disease;
Fusicoccum sp. UNK	/	Nectriaceae	This is a pathogen associated with ash dieback cankers disease;
Ganoderma lucidum UNK	/	Polyporaceae	Coot rot;
Ganoderma philippii TP	*Ganoderma philippii*	Polyporaceae	Coffee red root rot;
Ganoderma pseudoferreum TP	*Ganoderma philippii*	Polyporaceae	Coffee root rot;
Geotrichum candidum UNK	*Dipodascus geotrichum*	Dipodascaceae	Postharvest sour rot;
Gibberella stilboides TP	*Fusarium stilboides*	Nectriaceae	Coffee bark disease;
Gibberella xylarioides TP	*Fusarium xylarioides*	Nectriaceae	Coffee vascular wilt disease;
Gliocladiopsis tenuis UNK	*Gliocladiopsis tenuis*	Nectriaceae	Root rot disease;
Globisporangium splendens UNK	*Globisporangium splendens*	Pythiaceae	Stems and roots rot;
Gloeosporium coffeanum TP	*Colletotrichum coffeanum*	Glomerellaceae	Coffee leaf blight/Berry rot;
Gloeosporium coffeicola TP	*Gloeosporium coffeicola*	Drepanopezizaceae	Coffee fruit rot, pink berry mold;
Gloeosporium sp. TP	/	Drepanopezizaceae	Coffee fruit rot, pink berry mold;
Glomerella cingulata TP	*Colletotrichum*	Glomerellaceae	Coffee leaf spot. dieback and anthracnose;

Glomerella coffeicola TP	*gloeosporioides* /	Glomerellaceae	Coffee anthracnose lesions on leaves and shoots;
Glomerella phomoides TP	*Colletotrichum gloeosporioides*	Glomerellaceae	Coffee leaf spot, dieback;
Guignardia coffeana TP	*Colletotrichum coffeanum*	Phyllostictaceae	Coffee leaf blight/berry rot;
Helicobasidium compactum UNK	*Helicobasidium longisporum*	Helicobasidiaceae	Root rot;
Helicobasidium longisporum UNK	*Helicobasidium longisporum*	Helicobasidiaceae	Root rot;
Helminthosporium coffeae TP	*Helminthosporium coffeae*	Massarinaceae	Coffee leaves disease;
Helminthosporium sp. UNK	/	Massarinaceae	Leaf spots;
Helminthosporium ubangiense TP	*Helminthosporium ubangiense*	Massarinaceae	Coffee leaves disease;
Hemileia coffeicola TP	*Hemileia coffeicola*	Incertae sedis	Coffee rust;
Hemileia vastatrix TP	*Hemileia vastatrix*	Incertae sedis	Coffee rust;
Hendersonia coffeae TP	/	Phaeosphaeriaceae	Coffee leaf spots;
Hymenochaete noxia TP	*Hymenochaete noxia*	Hymenochaetaceae	Coffee brown root disease;
Hypoxylon deustum - (*Kretzschmaria deusta*) TP	/	Hypoxylaceae	Coffee soft rot;
Hypoxylon stygium UNK	*Annulohypoxylon stygium*	Hypoxylaceae	Rot disease;
Hirneola polytricha UNK	*Auricularia cornea*	Auriculariaceae	Rot disease;
Irenina coffeae TP	*Asteridiella glabra*	Meliolaceae	Coffee leaves disease;
Irenina glabra TP	*Asteridiella glabra*	Meliolaceae	Coffee leaves disease;
Isariopsis acanthacearum UNK	*Dactylaria acanthacearum*	Mycosphaerellaceae	Leaf spots;
Irpex flavus UNK	*Flavodon flavus*	Irpicaceae	Root disease;
Koleroga noxia TP	*Rhizoctonia noxia*	Ceratobasidiaceae	Coffee white-thread blight and black rot;
Laestadia coffeicola TP	/	Gnomoniaceae	Coffee circular whitish leaf spot;
Lasiodiplodia pseudotheobromae TP	*Lasiodiplodia pseudotheobromae*	Botryosphaeriaceae	Coffee dieback/fruit rot;
Lasiodiplodia theobromae TP	*Lasiodiplodia theobromae*	Botryosphaeriaceae	coffee dieback/fruit rot;
Leptoporus lignosus TP	*Rigidoporus microporus*	Irpicaceae/Meripilaceae	Coffee white root rot disease;
Leptosphaeria canephorae UNK	*Leptosphaeria canephorae*	Leptosphaeriaceae	Leaf spots;
Leptosphaeria coffaeicida TP	/	Leptosphaeriaceae	Coffee leaves disease;
Leptosphaeria coffeicola TP	/	Leptosphaeriaceae	Coffee leaves disease;
Leptosphaeria coffeigena TP	*Leptosphaeria coffeigena*	Leptosphaeriaceae	Coffee leaves disease;
Leptosphaeria coffeigena var. longirostrata TP	*Leptosphaeria coffeigena*	Leptosphaeriaceae	Coffee leaves disease;
Leptosphaeria pusilla TP	*Leptosphaeria pusilla*	Leptosphaeriaceae	Coffee leaves disease;
Leptosphaeria tonduzi TP	*Leptosphaeria tonduzii*	Leptosphaeriaceae	Coffee leaves disease;
Limacinia sp. UNK	/	Capnodiaceae	Sooty mold;

Macrophoma corchori TP	*Macrophomina phaseolina*	Botryosphaeriaceae	Coffee stem rot disease;
Macrophomina phaseoli TP	*Macrophomina phaseolina*	Botryosphaeriaceae	Coffee seedling blight, root and stem rot disease;
Macrophomina phaseolina TP	*Macrophomina phaseolina*	Botryosphaeriaceae	Coffee root disease;
Marasmiellus scandens TP	*Marasmiellus scandens*	Omphalotaceae	Coffee thread blight disease;
Marasmius scandens TP	*Marasmiellus scandens*	Omphalotaceae	Coffee thread blight disease;
Marasmius equicrinis UNK	*Marasmius crinis-equi*	Marasmiaceae	Thread blight;
Marasmius sp. TP	/	Marasmiaceae	Coffee white thread blight;
Microdiplodia sp. UNK	/	Botryosphaeriaceae	Leaf spot;
Micropeltella mulleri TP	*Micropeltella mulleri*	Micropeltidaceae	This is a virus pathogen causing coffee mosaic;
Micropeltis alanata TP PP	*Micropeltis applanata*	Micropeltidaceae	Coffee flyspeck;
Micropeltis mucosa TP	*Dictyothyriella mucosa*	Micropeltidaceae	Coffee disease to form small, black, superficial fruiting bodies;
Microxyphium sp. UNK	/	Coccodiniaceae	Sooty mold;
Mycena citricolor TP	*Mycena citricolor*	Mycenaceae	Coffee leaf spots;
Mycosphaerella coffeae TP	*Mycosphaerella coffeae*	Mycosphaerellaceae	Coffee leaf spot;
Mycosphaerella coffeicola TP	*Cercospora coffeicola*	Mycosphaerellaceae	Coffee brown-eye leaf spot/Berry blotch;
Myrothecium advena TP	*Paramyrothecium roridum*	Incertae sedis	Coffee target spot;
Myrothecium roridum TP	*Paramyrothecium roridum*	Incertae sedis	Coffee plant and seedlings leaf spot and stem necrosis;
Myrothecium sp. TP	/	Incertae sedis	Coffee leaf spots;
Nectria cinnabarina UNK	*Nectria cinnabarina*	Nectriaceae	Coral spot;
Nectria coffeigena TP	*Nectria coffeigena*	Nectriaceae	Coffee canker;
Nectria dodgei TP	*Nectria dodgei*	Nectriaceae	Coffee canker and dieback;
Nectria sp. UNK	/	Nectriaceae	Canker;
Nematospora coryli UNK	*Eremothecium coryli*	Eremotheciaceae	Yeast spot, bean rot;
Nematospora gossypii TP	*Eremothecium gossypii*	Eremotheciaceae	This is a pathogen that is pathogenic to coffee spp.;
Neocapnodium tanakae - (*Capnodium tanakae*) UNK	*Neocapnodium tanakae*	Trichomeriaceae	Sooty mold;
Neofusicoccum parvum UNK	*Neofusicoccum parvum*	Botryosphaeriaceae	Cankers, dieback and blight;
Nigrospora oryzae UNK	*Nigrospora oryzae*	Incertae sedis	Seedling/leaf blight;
Nigrospora sp. UNK	/	Incertae sedis	Seed rots;
Nigrospora sphaerica UNK	*Nigrospora oryzae*	Incertae sedis	Seedling/leaf blight;
Omphalia flavida TP	*Mycena citricolor*	Incertae sedis	Coffee leaf spot;
Ophionectria foliicola TP	/	Nectriaceae	Coffee leaves disease;
Oraniella coffeicola TP	*Massarina coffeicola*	Massarinaceae	Coffee disease;
Paracapnodium brasilense TP	*Scorias brasiliensis*	Capnodiaceae	Coffee black spot;
Paramyrothecium roridum TP	*Paramyrothecium roridum*	Stachybotryaceae	Coffee leaf spot;

Pellicularia filamentosa TP	*Rhizoctonia solani*	Ceratobasidiaceae	Coffee wilting/ brown necrosis of the lower taproot and blight disease;
Pellicularia koleroga - (*Rhizoctonia noxia*) TP	*Corticium koleroga*	Ceratobasidiaceae	Coffee white-thread blight and black rot;
Pellicularia sp. TP	/	Ceratobasidiaceae	Coffee white-thread blight and black rot;
Pestalotia coffeae TP	*Pestalotiopsis coffeae*	Amphisphaeriaceae	Coffee Leaf spot / stem blight;
Pestalotia coffeicola TP	/	Amphisphaeriaceae	Coffee Leaf spot / stem blight;
Pestalotia sp. TP	/	Amphisphaeriaceae	Coffee Leaf spot / stem blight;
Pestalotiopsis coffeae-arabicae TP	*Pestalotiopsis coffeae-arabicae*	Pestalotiopsidaceae	Coffee red-brown leaf spots;
Pestalotiopsis elasticae UNK	*Pestalotia elasticae*	Pestalotiopsidaceae	Leaf spots;
Pestalotiopsis kenyana TP/PP	*Pestalotiopsis kenyana*	Pestalotiopsidaceae	Coffee blight disease, gray blight, severe chlorosis, and various postharvest diseases;
Pestalotiopsis royenae TP	*Pestalotiopsis royenae*	Pestalotiopsidaceae	Coffee leaf streak;
Pestalotiopsis sp. TP/PP	/	Pestalotiopsidaceae	Coffee leaf spots, gray blight, severe chlorosis, and various postharvest diseases;
Pestalotiopsis theae UNK	*Pseudopestalotiopsis theae*	Pestalotiopsidaceae	Leaf spots;
Pestalotiopsis versicolor TP	*Pestalotiopsis versicolor*	Pestalotiopsidaceae	Coffee anthracnose;
Pestalozzia coffeae - (*Pestalotia coffeae*) TP	*Pestalotiopsis coffeae*	Amphisphaeriaceae	Coffee leaves disease;
Peyronellaea coffeae-arabicae TP	*Didymella coffeae-arabicae*	Didymellaceae	Coffee canker and dieback diseases;
Phaeosaccardinula javanica - (*Limacinula javanica*) UNK	*Chaetothyrium javanicum*	Chaetothyriaceae	Sooty mold;
Phanerochaete salmonicolor TP	*Erythricium salmonicolor*	Phanerochaetaceae	Coffee pink disease;
Phellinus lamaensis UNK	/	Hymenochaetaceae	Brown root rot;
Phellinus noxius - (*Phellinidium noxium*) TP	*Pyrrhoderma noxium*	Hymenochaetaceae	Coffee brown root rot;
Phoma coffeae-arabicae TP	*Didymella coffeae-arabicae*	Didymellaceae	Coffee canker and dieback diseases;
Phoma coffeicola TP	/	Didymellaceae	Coffee leaf spot;
Phoma costaricensis TP	*Phoma costaricensis*	Didymellaceae	Coffee leaf spot;
Phoma costarricensis TP	*Phoma costaricensis*	Didymellaceae	Coffee leaves, stems and fruits disease;
Phoma leveillei TP	*Phoma leveillei*	Didymellaceae	Coffee necrotic lesions, leaf spot;
Phoma pereupyrena TP	*Ectophoma pomi*	Didymellaceae	Coffee blight and leaf spot;
Phoma sorghina UNK	*Epicoccum sorghinum*	Didymellaceae	Leaf spot, stem necrosis, fruit rotting;
Phoma sp. TP	/	Didymellaceae	Coffee leaf spot;
Phoma tarda - (*Boeremia exigua var. coffeae*) TP	*Ascochyta tarda*	Didymellaceae	Coffee leaf blight and stem die- back;
Phomopsis coffeae TP	*Phomopsis coffeae*	Diaporthaceae	Coffee canker;
Phomopsis heveicola TP	*Phomopsis heveicola*	Diaporthaceae	Coffee leaf blight;
Phomopsis sp. TP	/	Diaporthaceae	Coffee canker, dieback and leaf spot;
Phyllosticta bokensis TP	*Phyllosticta bokensis*	Phyllostictaceae	Coffee leaf spot;
Phyllosticta camoensis	/	Phyllostictaceae	Coffee leaf spot;

TP			
Phyllosticta coffeae-arabicae - (*Phyllosticta bokensis*) TP	/	Phyllostictaceae	Coffee leaf spot;
Phyllosticta coffeae-libericae TP	*Phyllosticta coffeae-libericae*	Phyllostictaceae	Coffee leaf spot;
Phyllosticta coffeicola TP	/	Phyllostictaceae	Coffee leaf spot;
Phyllosticta sp. TP	/	Phyllostictaceae	Coffee leaf spots;
Phyllosticta usteri TP	*Phyllosticta usteri*	Phyllostictaceae	Coffee leaf spot;
Physalospora sp. UNK	/	Hyponectriaceae	Leaf spot, canker;
Phytophthora palmivora TP	*Phytophthora palmivora*	Peronosporaceae	Coffee black pod disease;
Podosporium sp. UNK	/	Incertae sedis	Stem blight, canker;
Polyporus coffeae TP	*Diacanthodes novoguineensis*	Polyporaceae	Coffee root disease;
Polyporus depauperatus UNK	/	Polyporaceae	Wood-rotting;
Polyporus rubidus TP	*Fomitopsis rubida*	Polyporaceae	Coffee root rot;
Polyporus zonalis UNK	*Rigidoporus lineatus*	Polyporaceae	Heart rot and butt;
Polystictus phaeus UNK	/	Hymenochaetaceae	Heart rot;
Polystictus occidentalis UNK	*Coriolopsis occidentalis*	Hymenochaetaceae	Heart rot;
Pyrenochaetopsis microspora TP	*Pyrenochaetopsis microspora*	Cucurbitariaceae	Coffee leaves disease;
Pyrrhoderma noxium - (*Phellinidium noxium*) TP	*Pyrrhoderma noxium*	Hymenochaetaceae	Coffee brown root rot;
Pythium periilum UNK	*Pythium periilum*	Pythiaceae	Root disease;
Pythium sp. TP	/	Pythiaceae	Damping-off affects coffee-seedlings in nurseries and root rot;
Pythium splendens UNK	*Globisporangium splendens*	Pythiaceae	Stems and roots rot;
Rhizoctonia bataticola TP	*Macrophomina phaseolina*	Ceratobasidiaceae	Coffee damping-off disease;
Rhizoctonia choussii TP	*Rhizoctonia choussii*	Ceratobasidiaceae	Coffee root rot disease;
Rhizoctonia lamellifera TP	*Macrophomina phaseolina*	Botryosphaeriaceae	Coffee root rot disease;
Rhizoctonia solani TP	*Rhizoctonia solani*	Ceratobasidiaceae	Coffee leaf blight, root rot and stem canker disease;
Rhizoctonia sp. TP	/	Ceratobasidiaceae	Coffee black rot;
Rigidoporus lignosus TP	*Rigidoporus microporus*	Meripilaceae	Coffee white root rot disease;
Rigidoporus microporus TP	*Rigidoporus microporus*	Meripilaceae	Coffee white root disease;
Rosellinia aquila TP	*Rosellinia arcuata*	Xylariaceae	Coffee root rot;
Rosellinia bunodes TP	*Dematophora bunodes*	Xylariaceae	Coffee black root rot;
Rosellinia lobayensis UNK	*Rosellinia lobayensis*	Xylariaceae	Root rot;
Rosellinia necatrix TP	*Dematophora necatrix*	Xylariaceae	Coffee white root rot;
Rosellinia pepo TP	*Dematophora pepo*	Xylariaceae	Coffee white root rot;
Rosellinia sp. TP	/	Xylariaceae	Coffee black rot root;
Roussoella mexicana TP	*Roussoella mexicana*	Roussoellaceae	Coffee leaf spots;
Sclerotium coffeicola TP	/	Incertae sedis	Coffee target-spot;

Sclerotium rolfsii TP	*Athelia rolfsii*	Incertae sedis	Coffee leaf blight;
Sclerotium sp. TP	/	Incertae sedis	Damping-off coffee seedlings;
Scolecopeltis longispora TP	*Micropeltis longispora*	Micropeltidaceae	Coffee leaves disease;
Scorias communis TP	*Scorias communis*	Capnodiaceae	Coffee sooty mold;
Septoria berkeleyi UNK	/	Mycosphaerellaceae	Pale-brown leaf spots;
Septoria coffeae TP	/	Mycosphaerellaceae	Coffee leaves disease;
Septoria coffeicola TP	/	Mycosphaerellaceae	Coffee circular white leaf spot with dark-brown margins;
Sphaerella coffeicola TP	*Cercospora coffeicola*	Mycosphaerellaceae	Coffee brown-eye leaf spot/berry blotch;
Sphaerella sp. UNK	/	Mycosphaerellaceae	Leaf spots;
Stilbella flavidum TP	*Decapitatus flavidus*	Incertae sedis	Coffee leaf spots;
Stilbum flavidum TP	*Decapitatus flavidus*	Chionosphaeraceae	Coffee leaf spots;
Stenella coffeae TP	*Stenella coffeae*	Teratosphaeriaceae	Coffee leaves disease;
Thanatephorus cucumeris TP	*Rhizoctonia solani*	Ceratobasidiaceae	Web blight on coffee bean and soybean;
Thielaviopsis neocaledoniae UNK	*Davidsoniella neocaledoniae*	Ceratocystidaceae	Black wood disease;
Thielaviopsis paradoxa TP	*Ceratocystis paradoxa*	Ceratocystidaceae	Coffee blight and black rot disease;
Trachysphaera fructigena TP,PP	*Trachysphaera fructigena*	Pythiaceae	Mealy pod, attacking fruits of all ages;
Trichopeltis pulchella UNK	/	Trichothyriaceae	Sooty mold;
Trichothecium roseum UNK	*Trichothecium roseum*	Incertae sedis	Pink mold;
Tripospermum gardneri UNK	*Tripospermum gardneri*	Capnodiaceae	Coffee sooty mold;
Triposporiopsis spinigera UNK	*Chaetothyrium spinigerum*	Trichomeriaceae	Sooty mold;
Ustulina deusta - (*Kretzschmaria deusta*) TP	/	Xylariaceae	Coffee soft rot;
Venturia coffeicola TP	/	Venturiaceae	Coffee leaves disease;
Vermicularia sp. UNK	/	Glomerellaceae	Plant disease;

All the pathogens are superscripts with TP, postharvest pathogens are superscript with PP and other diseases that can't be confirmed on coffee are superscript with UNK.

Table S5. Post-harvest fungal diseases reported on coffee.

Species	Reference	Host	Specific part of coffee	Location	Toxin
Penicillium verruculosum	[10]	Arabica/robusta/liberica/excelsea	Green/dried coffee beans	Philippines	Ochratoxin-A
Penicillium sp.	[31]	Arabica/robusta	Coffee beans	Philippines	Ochratoxin-A, sterigmatocystin
Byssochlamys spectabilis	[15]	*Coffea arabica*	Roasted coffee	Local markets in Nayarit and Mexico	Ochratoxin-A
Aspergillus versicolor	[9,15]	*Coffea arabica*	Coffee beans/Roasted coffee	Local markets in Nayarit and Mexico, Brazil	Ochratoxin-A
Aspergillus westerdijkiae	[4,10]	Arabica/robusta/liberica/excelsea	Green/dried coffee bean	Thailand, Philippines	Ochratoxin-A, ochratoxin-B
Aspergillus sulphureus	[9]	*Coffea arabica*	Green coffee bean	Brazil	Ochratoxin-A
Aspergillus steynii	[4]	Arabica	Dried coffee bean	Thailand	Ochratoxin-A, ochratoxin-B
Aspergillus sp. *seção Circumdati*	[3]	/	Coffee beans	Minas Gerais, Brazil	Ochratoxin-A
Aspergillus sp.	[8,31]	Arabica/robusta	Arabica parchment coffee/green coffee/Coffee beans	Thailand, Philippines	Ochratoxin-A, sterigmatocystin
Aspergillus sclerotiorum	[4,8,9]	*Coffea arabica*	Green/dried coffee bean	Brazil, Thailand	Ochratoxin-A
Aspergillus petrakii	[9]	*Coffea arabica*	Green coffee bean	Brazil	Ochratoxin-A
Aspergillus ostianus	[3,9]	Arabica	Arabica parchmentcoffee/green coffee bean	Thailand, Brazil, Minas Gerais	Ochratoxin-A
Aspergillus ochraceus	[3,8–11]	Arabica/robusta/liberica/excelsea/*Coffea arabica var. bourbon*	Green coffee beans/Arabica parchment coffee /Coffee cherries	Saudi Arabia markets, Thailand, Brazil, Minas Gerais, Xalapa, Mexico, Philippines	Ochratoxin-A
Aspergillus niger	[3,4,10]	*Coffea arabica var. bourbon*/robusta/arabica/liberica/excelsea	Coffee cherries/dried coffee bean/ Green coffee beans	Xalapa, Mexico, Thailand, Minas Gerais, Brazil, Philippines	Ochratoxin-A, ochratoxin-B
Aspergillus melleus	[4]	Arabica	Dried coffee bean	Thailand	Ochratoxin-A
Aspergillus japonicus	[10]	Arabica/robusta/liberica/excelsea	Dried green coffee beans	Philippines	Ochratoxin-A
Aspergillus insulicola	[9]	*Coffea arabica*	Green coffee bean	Brazil	Ochratoxin-A
Aspergillus flavus	[9,11]	*Coffea arabica*	Green coffee bean	Brazil, Saudi Arabia markets	aflatoxin B1/B2
Aspergillus elegans	[9]	*Coffea arabica*	Green coffee bean	Brazil	Ochratoxin-A
Aspergillus	[4,7,10]	Arabica/robusta/liberi	Dried coffee bean	Thailand, Philippines	Ochratoxin-A

carbonarius		ca/excelsea			
Aspergillus candidus	[8]	Arabica	Arabica parchment coffee/green coffee	Thailand	Ochratoxin-A
Aspergillus awamori	[8]	Arabica	Arabica parchment coffee/green coffee	Thailand	Ochratoxin-A
Aspergillus auricomus	[9]	*Coffea arabica*	Green coffee bean	Brazil	Ochratoxin-A
Fusarium sp.	[31]	Arabica/robusta	Coffee beans	Philippines	Ochratoxin-A, sterigmatocystin
Mucor sp.	[31]	Arabica/robusta	Coffee beans	Philippines	Ochratoxin-A, sterigmatocystin
Rhizopus sp.	[31]	Arabica/robusta	Coffee beans	Philippines	Ochratoxin-A, sterigmatocystin

References

1. Santamaría, J.; Bayman, P. Fungal epiphytes and endophytes of coffee leaves (*Coffea arabica*). *Microbial ecology* **2005**, *50*, 1–8.
2. de Almeida, Â.B.; Corrêa, I.P.; Furuie, J.L.; de Farias Pires, T.; do Rocio Dalzoto, P.; Pimentel, I.C. Inhibition of growth and ochratoxin A production in *Aspergillus* species by fungi isolated from coffee beans. *Brazilian Journal of Microbiology* **2019**, *50*, 1091–1098.
3. da Silva, S.A.; Pereira, R.G.F.A.; de Azevedo Lira, N.; da Gloria, E.M.; Chalfoun, S.M.; Batista, L.R. Fungi associated to beans infested with coffee berry borer and the risk of ochratoxin A. *Food Control* **2020**, *113*, 107204.
4. Noonim, P.; Mahakarnchanakul, W.; Nielsen, K.F.; Frisvad, J.C.; Samson, R.A. Isolation, identification and toxigenic potential of ochratoxin A-producing *Aspergillus* species from coffee beans grown in two regions of Thailand. *International journal of food microbiology* **2008**, *128*, 197–202.
5. Vega, F.E.; Posada, F.; Aime, M.C.; Pava-Ripoll, M.; Infante, F.; Rehner, S.A. Entomopathogenic fungal endophytes. *Biological control* **2008**, *46*, 72–82.
6. Estupiñan-López, L.; Campos, V.P.; da Silva Júnior, J.C.; Pedroso, M.P.; Terra, W.C.; da Silva, J.C.P.; de Paula, L.L. Volatile compounds produced by *Fusarium* spp. isolated from *Meloidogyne paranaensis* egg masses and corticous root tissues from coffee crops are toxic to *Meloidogyne incognita*. *Tropical Plant Pathology* **2018**, *43*, 183–193.
7. Sousa, T.; Batista, L.; Passamani, F.; Lira, N.; Cardoso, M.; Santiago, W.; Chalfoun, S. Evaluation of the effects of temperature on processed coffee beans in the presence of fungi and ochratoxin A. *Journal of Food Safety* **2019**, *39*, e12584.
8. Kuntawee, S.; Akarapisan, A. Isolation and identification of *Aspergillus* species producing Ochratoxin a in Arabica coffee beans. *J Agric Technol* **2015**, *11*, 1235–1242.
9. Batista, L.R.; Chalfoun, S.M.; Prado, G.; Schwan, R.F.; Wheals, A.E. Toxigenic fungi associated with processed (green) coffee beans (*Coffea arabica* L.). *International Journal of Food Microbiology* **2003**, *85*, 293–300.
10. Alvindia, D.G.; de Guzman, M.F. Survey of Philippine coffee beans for the presence of ochratoxigenic fungi. *Mycotoxin research* **2016**, *32*, 61–67.
11. Bokhari, F.M. Mycotoxins and toxigenic fungi in arabic coffee beans in Saudi Arabia. *Adv Biol Res* **2007**, *1*, 56–66.
12. Djadjiti, N.; Aziz, M.A.; Esther, O.K.; Dane, S. Isolation and Identification of Fungal Pathogens Contaminating Some Coffee Powder Marketed in the City of Abuja. *Journal of Research in Medical and Dental Science* **2020**, *8*, 172–175.
13. Eida, M.F.; Nagaoka, T.; Asaki, J.; Kouno, K. Evaluation of cellulolytic and hemicellulolytic abilities of fungi isolated from coffee residue and sawdust composts. *Microbes and environments* **2009**, 1105090302–1105090302.
14. Pasin, L.A.A.P.; Almeida, J.R.d.; Abreu, M.S.d. Fungos associados a grãos de cinco cultivares de café (*Coffea arabica* L.). *Acta Botanica Brasilica* **2009**, *23*, 1129–1132.
15. Casas-Junco, P.P.; Ragazzo-Sánchez, J.A.; de Jesus Ascencio-Valle, F.; Calderón-Santoyo, M. Determination of potentially mycotoxigenic fungi in coffee (*Coffea arabica* L.) from Nayarit. *Food science and biotechnology* **2018**, *27*, 891–898.
16. Chaves, F.C.; Gianfagna, T.J.; Aneja, M.; Posada, F.; Peterson, S.W.; Vega, F.E. *Aspergillus oryzae* NRRL 35191 from coffee, a non-toxigenic endophyte with the ability to synthesize kojic acid. *Mycological progress* **2012**, *11*, 263–267.
17. González-Osorio, H.; Botero, C.E.G.; Rivera, R.D.M.; Vega, N.W.O. Screening for phosphate-solubilizing fungi from colombian andisols cultivated with coffee (*Coffea arabica* L.). *Coffee Science* **2020**, e151666.
18. Hongsanan, S.; Tian, Q.; Hyde, K.; Chomnunti, P. Two new species of sooty moulds, *Capnodium coffeicola* and *Conidiocarpus plumeriae* in Capnodiaceae. *Mycosp* **2015**, *6*, 814–824.
19. Maharachchikumbura, S.; Haituk, S.; Pakdeeniti, P.; Al-Sadi, A.; Hongsanan, S.; Chomnunti, P.; Cheewangkoon, R. *Phaeosaccardinula coffeicola* and *Trichomerium chiangmaiensis*, two new species of Chaetothyriales (Eurotiomycetes) from Thailand. *Mycosphere* **2018**, *9*, 769–778.

20. Bongiorno, V.A.; Rhoden, S.A.; Garcia, A.; Polonio, J.C.; Azevedo, J.L.; Pereira, J.O.; Pamphile, J.A. Genetic diversity of endophytic fungi from *Coffea arabica* cv. IAPAR-59 in organic crops. *Annals of Microbiology* **2016**, *66*, 855–865.
21. Oliveira, R.; Souza, R.; Lima, T.; Cavalcanti, M. Endophytic fungal diversity in coffee leaves (*Coffea arabica*) cultivated using organic and conventional crop management systems. *Mycosphere* **2014**, *5*, 523–530.
22. Saucedo-García, A.; Anaya, A.L.; Espinosa-García, F.J.; González, M.C. Diversity and communities of foliar endophytic fungi from different agroecosystems of *Coffea arabica* L. in two regions of Veracruz, Mexico. *PloS one* **2014**, *9*, e98454.
23. Monteiro, M.C.P.; Alves, N.M.; de Queiroz, M.V.; Pinho, D.B.; Pereira, O.L.; de Souza, S.M.C.; Cardoso, P.G. Antimicrobial activity of endophytic fungi from coffee plants. *Bioscience Journal* **2017**, *33*.
24. Souza, A.G.C.; Rodrigues, F.Á.; Maffia, L.A.; Mizubuti, E.S.G. Infection process of *Cercospora coffeicola* on coffee leaf. *Journal of phytopathology* **2011**, *159*, 6–11.
25. Fernandes, M.d.R.V.; Silva, T.A.C.; Pfenning, L.H.; Costa-Neto, C.M.d.; Heinrich, T.A.; Alencar, S.M.d.; Lima, M.A.d.; Ikegaki, M. Biological activities of the fermentation extract of the endophytic fungus *Alternaria alternata* isolated from *Coffea arabica* L. *Brazilian journal of pharmaceutical sciences* **2009**, *45*, 677–685.
26. Prihastuti, H.; Cai, L.; Chen, H.; McKenzie, E.; Hyde, K. Characterization of *Colletotrichum* species associated with coffee berries in northern Thailand. *Fungal Diversity* **2009**, *39*, 89–109.
27. Serrato-Diaz, L.M.; Mariño, Y.A.; Bayman, P. Pathogens causing Anthracnose and fruit rots of coffee associated with the coffee berry borer and the entomopathogenic fungus *Beauveria bassiana* in Puerto Rico. *Phytopathology* **2020**, *110*, 1541–1552.
28. Kenny, M.; Galea, V.; Price, T. Germination and growth of *Colletotrichum acutatum* and *Colletotrichum gloeosporioides* isolates from coffee in Papua New Guinea and their pathogenicity to coffee berries. *Australasian Plant Pathology* **2012**, *41*, 519–528.
29. María del Carmen, H.R.; Evans, H.C.; de Abreu, L.M.; de Macedo, D.M.; Ndacnou, M.K.; Bekele, K.B.; Barreto, R.W. New species and records of *Trichoderma* isolated as mycoparasites and endophytes from cultivated and wild coffee in Africa. *Scientific reports* **2021**, *11*, 1–30.
30. Vale, P.A.S.; de Resende, M.L.V.; dos Santos Botelho, D.M.; de Andrade, C.C.L.; Alves, E.; Ogoshi, C.; Guimarães, S.d.S.C.; Pfenning, L.H. Epitypification of *Cercospora coffeicola* and its involvement with two different symptoms on coffee leaves in Brazil. *European Journal of Plant Pathology* **2021**, *159*, 399–408.
31. Culliao, A.G.L.; Barcelo, J.M. Fungal and mycotoxin contamination of coffee beans in Benguet province, Philippines. *Food Additives & Contaminants: Part A* **2015**, *32*, 250–260.
32. Gizaw, B.; Tsegaye, Z.; Tefera, G. Isolation, identification and characterization of yeast species from coffee waste collected from Sidama and Gedio zone. *Journal of Yeast and Fungal Research* **2016**, *7*, 47–53.
33. Lu, L.; Tibpromma, S.; Karunarathna, S.; Thiyagaraja, V.; XU, J.C.; Jayawardena, R.S.; Lumyong, S.; Hyde, K.D. Taxonomic and phylogenic appraisal of a novel species and a new record of Stictidaceae from coffee in Yunnan Province, China. *Phytotaxa* **2021**, *528*, 111–124.
